# *In Vivo* PET Tracking of ^89^Zr-Labeled Vγ9Vδ2 T Cells to Mouse Xenograft Breast Tumors Activated with Liposomal Alendronate

**DOI:** 10.1016/j.ymthe.2018.10.006

**Published:** 2018-10-16

**Authors:** Francis Man, Lindsay Lim, Alessia Volpe, Alberto Gabizon, Hilary Shmeeda, Benjamin Draper, Ana C. Parente-Pereira, John Maher, Philip J. Blower, Gilbert O. Fruhwirth, Rafael T.M. de Rosales

**Affiliations:** 1 School of Biomedical Engineering & Imaging Sciences, King’s College London, St Thomas’ Hospital, London SE1 7EH, UK; 2 Oncology Institute, Shaare Zedek Medical Center and Hebrew University−School of Medicine, Jerusalem 9103102, Israel; 3 School of Cancer and Pharmaceutical Sciences, King’s College London, Guy’s Hospital, London SE1 9RT, UK

**Keywords:** cancer immunotherapy, cell immunotherapy, liposome, PET, cell tracking, bisphosphonate, zirconium-89, nanomedicine

## Abstract

Gammadelta T (γδ-T) cells are strong candidates for adoptive immunotherapy in oncology due to their cytotoxicity, ease of expansion, and favorable safety profile. The development of γδ-T cell therapies would benefit from non-invasive cell-tracking methods and increased targeting to tumor sites. Here we report the use of [^89^Zr]Zr(oxinate)_4_ to track Vγ9Vδ2 T cells *in vivo* by positron emission tomography (PET). *In vitro*, we showed that ^89^Zr-labeled Vγ9Vδ2 T cells retained their viability, proliferative capacity, and anti-cancer cytotoxicity with minimal DNA damage for amounts of ^89^Zr ≤20 mBq/cell. Using a mouse xenograft model of human breast cancer, ^89^Zr-labeled γδ-T cells were tracked by PET imaging over 1 week. To increase tumor antigen expression, the mice were pre-treated with PEGylated liposomal alendronate. Liposomal alendronate, but not placebo liposomes or non-liposomal alendronate, significantly increased the ^89^Zr signal in the tumors, suggesting increased homing of γδ-T cells to the tumors. γδ-T cell trafficking to tumors occurred within 48 hr of administration. The presence of γδ-T cells in tumors, liver, and spleen was confirmed by histology. Our results demonstrate the suitability of [^89^Zr]Zr(oxinate)_4_ as a cell-labeling agent for therapeutic T cells and the potential benefits of liposomal bisphosphonate treatment before γδ-T cell administration.

## Graphical Abstract

**Figure:**
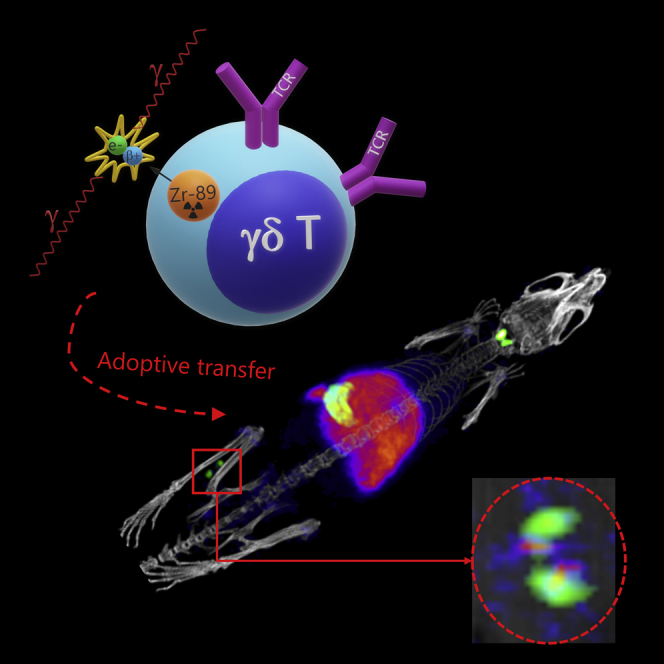

## Teaser

Man et al. demonstrate that [^89^Zr]Zr(oxinate)_4_ allows *in* vivo PET imaging of therapeutic γδ-T cells over 1 week. Furthermore, the trafficking of γδ-T cells to the tumor is increased by treatment with a liposomal aminobisphosphonate drug. This radiotracer is potentially translatable for clinical trials of therapeutic T cells.

## Introduction

Adoptive transfer of therapeutic T cells is a growing field in immuno-oncology, with spectacular clinical results against melanoma and hematological cancers.^1^, ^2^, ^3^ Gammadelta-T (γδ-T) cell therapy is one type of T cell therapy being explored, with recent data showing intra-tumoral γδ-T cells are the single most favorable prognostic immune cell infiltrate.^4^ γδ-T cells perform roles belonging to both adaptive and innate immunity, playing a significant role in anti-infectious and anti-tumor immune surveillance.^5^ Activated γδ-T cells are highly cytotoxic, enhance the function of other immune cells, and act as antigen-presenting cells.^6^ In humans, the Vγ9Vδ2 subtype of γδ-T cells represents 1%–5% of circulating CD3^+^ T cells.^6^ Their potent cytotoxicity and high proliferative capacity have made them candidates of choice for cancer immunotherapy.^7^

The unique activation of Vγ9Vδ2 cells by phosphoantigens such as isopentenyl pyrophosphate (IPP)^8^ allows them to discriminate between normal and metabolically disordered cells based on IPP expression levels.^9^ The activation and targeting of γδ-T cells to tumor tissue could, therefore, be improved by selectively increasing the presentation of phosphoantigens in cancer cells, for example, by using liposome- or nanocarrier-based formulations of aminobisphosphonate drugs (NBPs).^10^ NBPs (e.g., pamidronate, alendronate, and zoledronate),^11^ which increase the expression of IPP in target cells by inhibiting farnesyl diphosphate synthase, are hydrophilic molecules that accumulate in bone, but not in other tissues, and are rapidly cleared from the circulation. Encapsulating alendronate in liposomes has been shown to increase the therapeutic efficacy of γδ-T cells in preclinical models.^12^, ^13^

Clinical studies of γδ-T cell immunotherapy have shown a good safety profile and efficacy comparable to second-line anticancer therapies, but they have also highlighted the need for improvements.^14^, ^15^ Unknown aspects of adoptive γδ-T cell therapy include their *in vivo* distribution and kinetics of arrival at the tumor site. Whole-body imaging is highly useful in this context by enabling *in vivo* tracking of administered cells. Many techniques exist for non-invasive cell tracking;^16^, ^17^, ^18^ however, only nuclear imaging, and particularly positron emission tomography (PET), provides sensitive and quantitative, whole-body information with adequate spatiotemporal resolution. Hence, methods to radiolabel and track therapeutic cells using positron-emitting radionuclides are likely to become important tools for cell immunotherapy.^19^

PET tracking of T cells has been performed with radiolabeled antibodies, antibody fragments, or lipophilic small molecules^20^, ^21^ and by reporter-gene imaging.^22^ When genetic engineering is not required, e.g., for γδ-T cells, a clinically applicable alternative to reporter-gene imaging is direct cell labeling with PET radionuclides. Immune cells have long been imaged clinically by single-photon emission computed tomography (SPECT) in this manner, for example, using [^111^In]In(oxinate)_3_ and [^99m^Tc]Tc-exametazime.^19^ In this regard, the clinically approved 8-hydroxyquinoline (oxine) has been recently shown to be an excellent ionophore for cell labeling with ^89^Zr (*t*_*1/2*_ = 78.4 hr, β^+^ = 22.3%).^23^, ^24^, ^25^ However, to the best of our knowledge, no study has evaluated its use for tracking γδ-T cells.

Here we report the first use of [^89^Zr]Zr(oxinate)_4_ for *in vitro* radiolabeling and *in vivo* tracking of human γδ-T cells, including the effects of radiolabeling on γδ-T cell functionality, proliferation, and DNA integrity. We applied this strategy in a xenograft model of breast cancer with an engineered cancer cell line that allows multimodal imaging to track tumor cells. A liposomal aminobisphosphonate was administered to increase T cell trafficking to the tumor.

## Results

### Radiotracer Labeling Efficiency and Retention in γδ-T Cells

**Figure 1:**
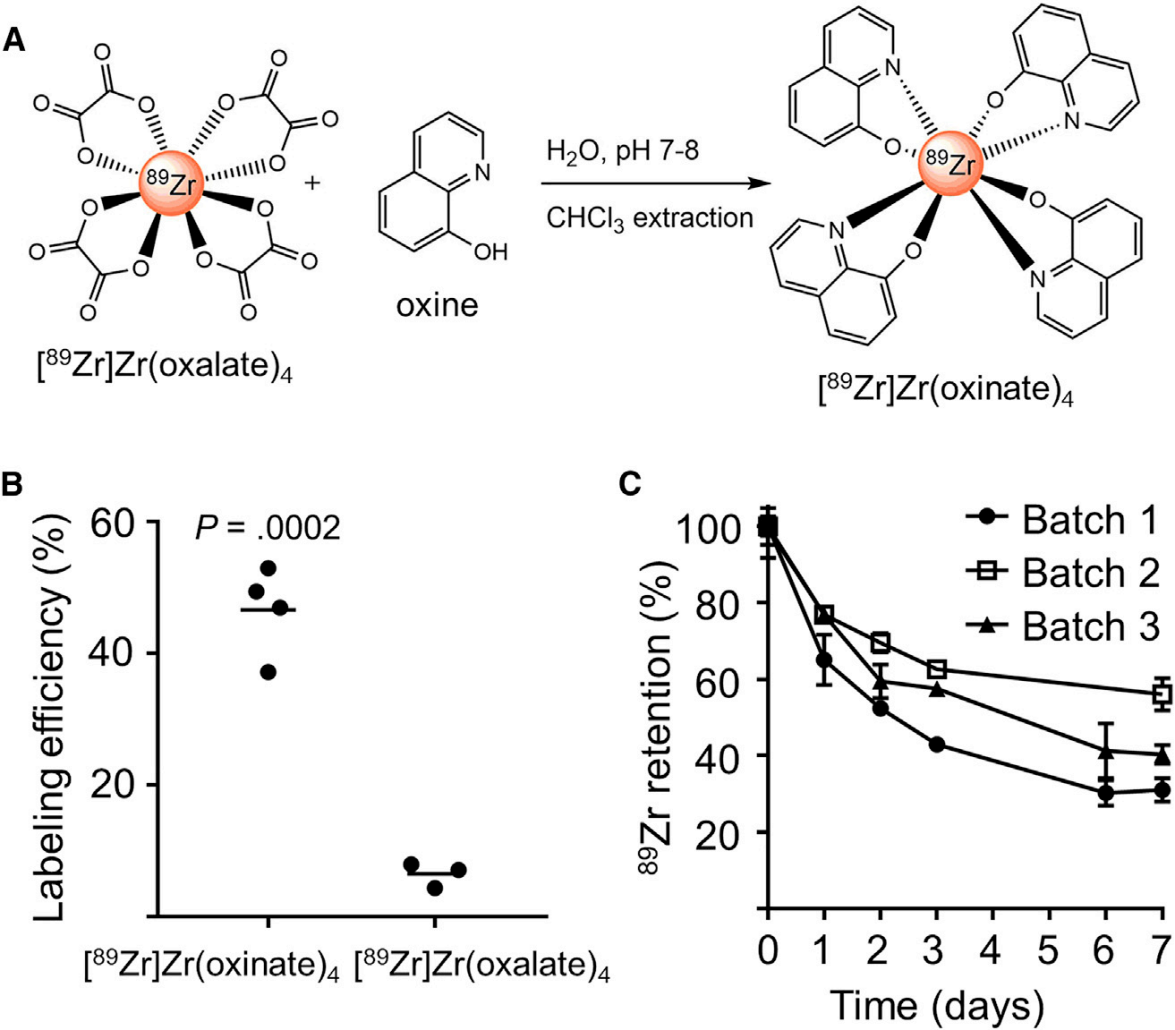
Radiotracer Synthesis and γδ-T Cell Radiolabeling (A) [^89^Zr]Zr(oxinate)_4_ synthesis. (B) Labeling efficiencies of γδ-T cells incubated with ^89^Zr-based tracers (63.2 ± 7.9 mBq/cell) 20 min at RT. Mean of N = 3–4 individual experiments (unpaired t test). (C) ^89^Zr retention by γδ-T cells over 7 days after labeling with [^89^Zr]Zr(oxinate)_4_ (average incorporated activity: 34.3 ± 6.0 mBq/cell). Mean ± SEM of triplicate measures for 3 cell batches.

[^89^Zr]Zr(oxinate)_4_ was obtained by mixing neutralized [^89^Zr]Zr(oxalate)_4_ with 8-hydroxyquinoline dissolved in chloroform (Figure 1A). The radiochemical yield was 77.6% ± 11.8% (mean ± SD, N = 21), and radiochemical purity established by thin-layer radiochromatography was >95% (Figure S1). γδ-T cell labeling efficiency with [^89^Zr]Zr(oxinate)_4_ (46.6% ± 3.4%, N = 4) was significantly higher than with [^89^Zr]Zr(oxalate)_4_ (6.5% ± 1.1%, N = 3; Figure 1B). To optimize radiolabeling conditions, cells were incubated with [^89^Zr]Zr(oxinate)_4_ (6−600 mBq/cell) for 10, 20, or 30 min at 4°C, room temperature (RT), or 37°C. We found no significant difference between incubation times and temperatures (Figure S2).

To study long-term tracer retention, radiolabeled γδ-T cells (25−40 mBq/cell) were cultured at 0.83 × 10^6^ cells/mL. After 24 hr, the percentage of cell-associated ^89^Zr was 72.9% ± 6.8% of the original activity, and 42.4% ± 12.6% after 1 week (N = 3; Figure 1C).

### *In Vitro* Assays of ^89^Zr-Radiolabeled γδ-T Cells

**Figure 2:**
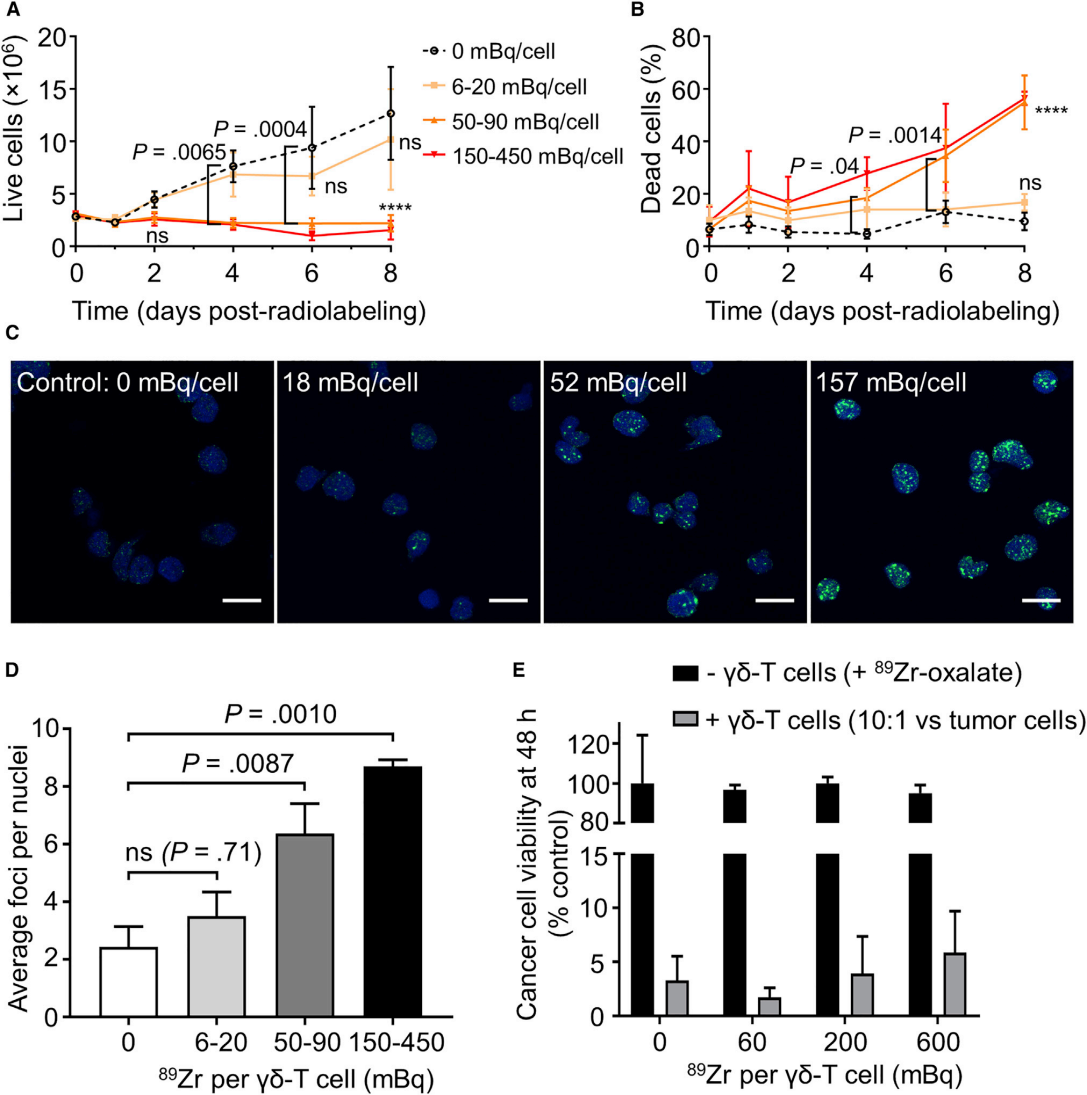
Assays of ^89^Zr-Radiolabeled γδ-T Cells (A and B) *In vitro* growth (A) and mortality (B) of radiolabeled γδ-T cells. Mean ± SEM of N = 4 independent experiments (except 150–450 mBq group, N = 2, not included in statistical analysis). ns: p > 0.05; ****p < 0.0001 versus unlabeled cells (2-way repeated-measures ANOVA, Dunnett’s correction for multiple comparisons). (C) Representative images of γ-H2AX foci (green) and nuclei (blue) in radiolabeled γδ-T cells (scale bars, 10 μm). (D) Average number of γ-H2AX foci per nuclei after radiolabeling. Mean ± SEM of N = 6, 5, 6, and 3 independent experiments (1-way ANOVA, Dunnett’s correction). (E) MDA-MB-231.hNIS-GFP tumor cell viability 48 hr after adding γδ-T cells or unchelated ^89^Zr, expressed as a percentage of control (tumor cells without γδ-T cells and ^89^Zr). Mean ± SEM of N = 3 independent experiments (2-way repeated-measures ANOVA, Dunnett’s correction).

The purity of *in vitro*-expanded γδ-T cells plateaued 13−15 days post-isolation (Figure S3), at which point they were radiolabeled. Cells labeled with 6−20 mBq/cell proliferated similarly to unlabeled cells (p ≥ 0.05; Figure 2A), while cells labeled with more than 50 mBq/cell ceased to proliferate *in vitro*, indicating a dose-dependent effect of ^89^Zr on γδ-T cell proliferation. A similar dose dependency was observed on γδ-T cell death (Figure 2B) and DNA damage, evaluated by the formation of γH2AX foci^26^ 1 hr after labeling (Figures 2C and 2D).

To evaluate the cytotoxic ability of radiolabeled γδ-T cells, we quantified the survival of MDA-MB-231.hNIS-GFP cancer cell monolayers. γδ-T cells labeled with up to 600 mBq/cell showed no significant difference in cancer cell killing compared to unlabeled γδ-T cells (Figure 2E). As a control, adding ^89^Zr up to 3 Bq/cancer cell in the medium was not toxic to cancer cells in the absence of γδ-T cells. Even in 30-fold excess, γδ-T cells showed no toxicity toward cancer cells in the absence of aminobisphosphonate (Figure S4).

### *In Vivo* PET Tracking of ^89^Zr-Radiolabeled γδ-T Cells

**Figure 3:**
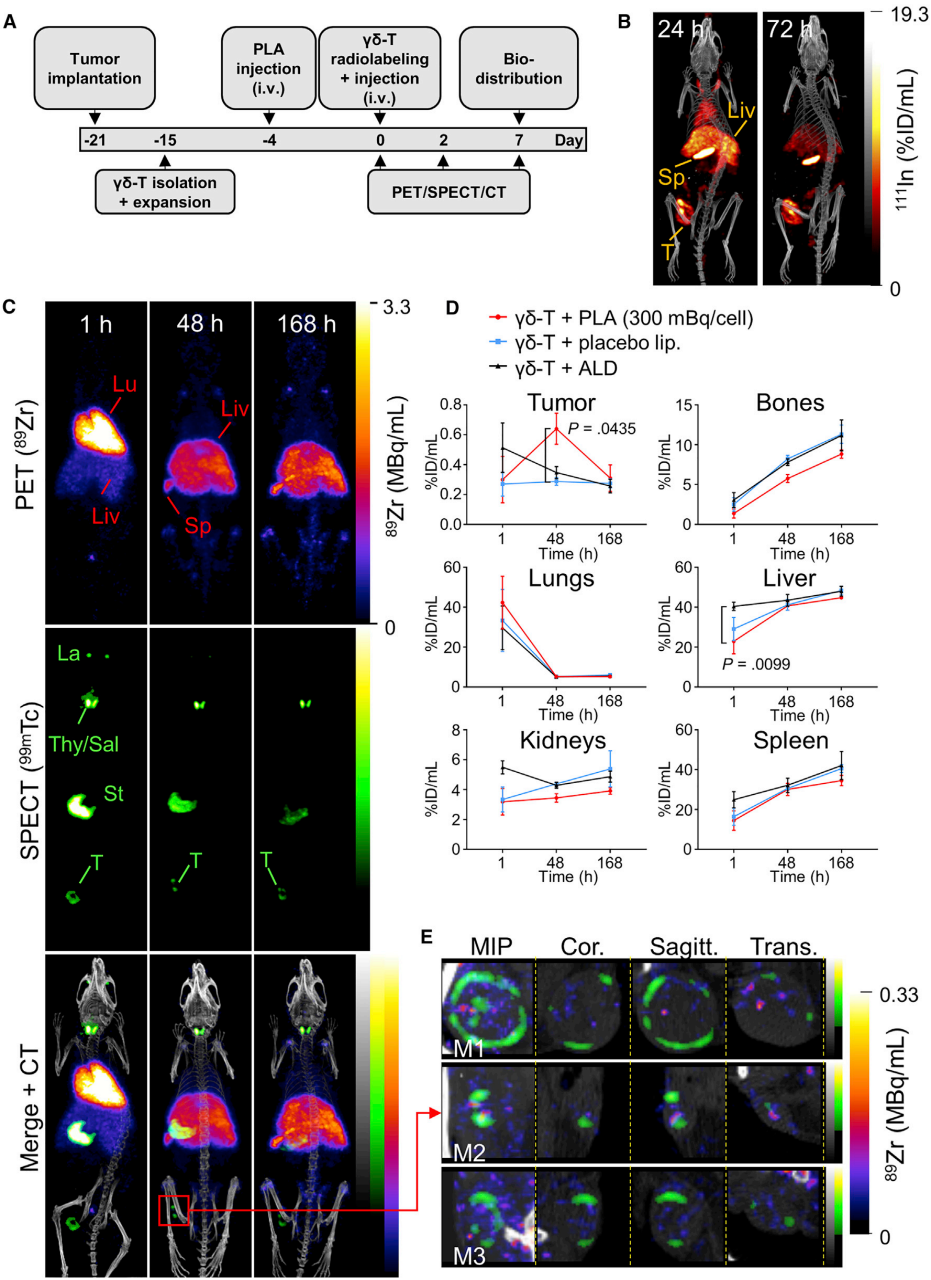
*In Vivo* Tracking of Radiolabeled γδ-T Cells (A) Experiment schedule. (B) Representative SPECT-CT images of MDA-MB-231.hNIS-GFP xenograft NSG mice 24 and 72 hr after ^111^In-labeled PLA administration. (C) Representative PET, SPECT, and CT (merged) scans of a PLA-treated SCID/beige mouse at 1, 48, and 168 hr post-injection of γδ-T cells. Liv, liver; Lu, lungs; Sp, spleen; T, tumor. Endogenous murine NIS expression also results in radiotracer uptake, giving rise to the following signals: La, lacrimal glands; St, stomach; and Thy/Sal, thyroid/salivary glands. (D) Time-activity curves from image-based quantification of ^89^Zr in selected organs. Mean ± SEM of N = 3–4 animals (repeated-measures MM analysis, Bonferroni correction for multiple comparisons). (E) Enlarged maximum intensity projection (MIP), coronal, sagittal, and transversal tumor views (merged PET- and SPECT-CT) in three PLA-treated mice (M1, M2, and M3), 48 hr after γδ-T cell injection.

^89^Zr-radiolabeled γδ-T cells were administered intravenously in a mouse xenograft model of breast cancer followed by PET imaging at 1 hr, 48 hr, and 7 days after injection. We imaged the hNIS-expressing cancer cells by SPECT using ^99m^TcO_4_^−^.^27^ We also evaluated the effect of PLA on γδ-T cell homing to tumor sites. The study schedule is provided in Figure 3A.

The PLA dosing schedule was established using ^111^In-labeled PLA, showing significant PLA tumor accumulation within 24–72 hr of administration (Figure 3B; Table S1). The experimental group (PLA treated) received radiolabeled γδ-T cells + PLA (5 mg/kg alendronate). Control groups (non-PLA treated) received radiolabeled γδ-T cells with placebo liposomes, non-liposomal alendronate, or saline. An additional control group received γδ-T cells killed by freeze-thawing to compare bio-distributions of viable and non-viable cells.

SPECT showed uptake of ^99m^TcO_4_^−^ in tumors and endogenous NIS-expressing organs (thyroid, salivary, and lacrimal glands and stomach; Figure 3C). At 1 hr after intravenous administration of ^89^Zr-radiolabeled γδ-T cells, PET revealed high amounts of radioactivity in the lungs in all groups, with signal also observed in the liver and spleen (Figures 3C and 3D). There was significantly higher uptake in the liver in the ALD group versus the PLA group. At tumor sites, the ^89^Zr signal was close to background (Figure S5). After 48 hr, ^89^Zr activity increased in the liver, spleen, and bones in all groups and decreased in the lungs. Uptake of ^89^Zr was observed at the tumor site only in the PLA group (Figure S5), suggesting the presence of radiolabeled γδ-T cells. Importantly, this was significantly higher in PLA-treated animals compared to control animals treated with non-liposomal alendronate (Figure 3D). Enlarged tumor views showed heterogeneity in tumor tissue, with live tissue, expressing a functional hNIS protein^18^, ^27^ and represented by a donut of ^99m^Tc signal surrounding a core of non-viable tumor cells (Figure 3E). ^89^Zr signal in tumors was heterogeneous, with some co-localizing with ^99m^Tc at the edges and foci of ^89^Zr signal inside the tumor. After 7 days, ^89^Zr activity remained high in the liver; increased in the spleen, bones, and kidneys; and was indistinguishable from background in tumors. Uptake values are provided in Table S2. Compared to other treatment groups, PET images of killed γδ-T cells showed a higher accumulation in the liver immediately after injection and increased uptake of ^89^Zr in the kidneys at later time points (Figure S6).

### *Ex Vivo* Bio-distribution of ^89^Zr-Radiolabeled γδ-T Cells

**Figure 4:**
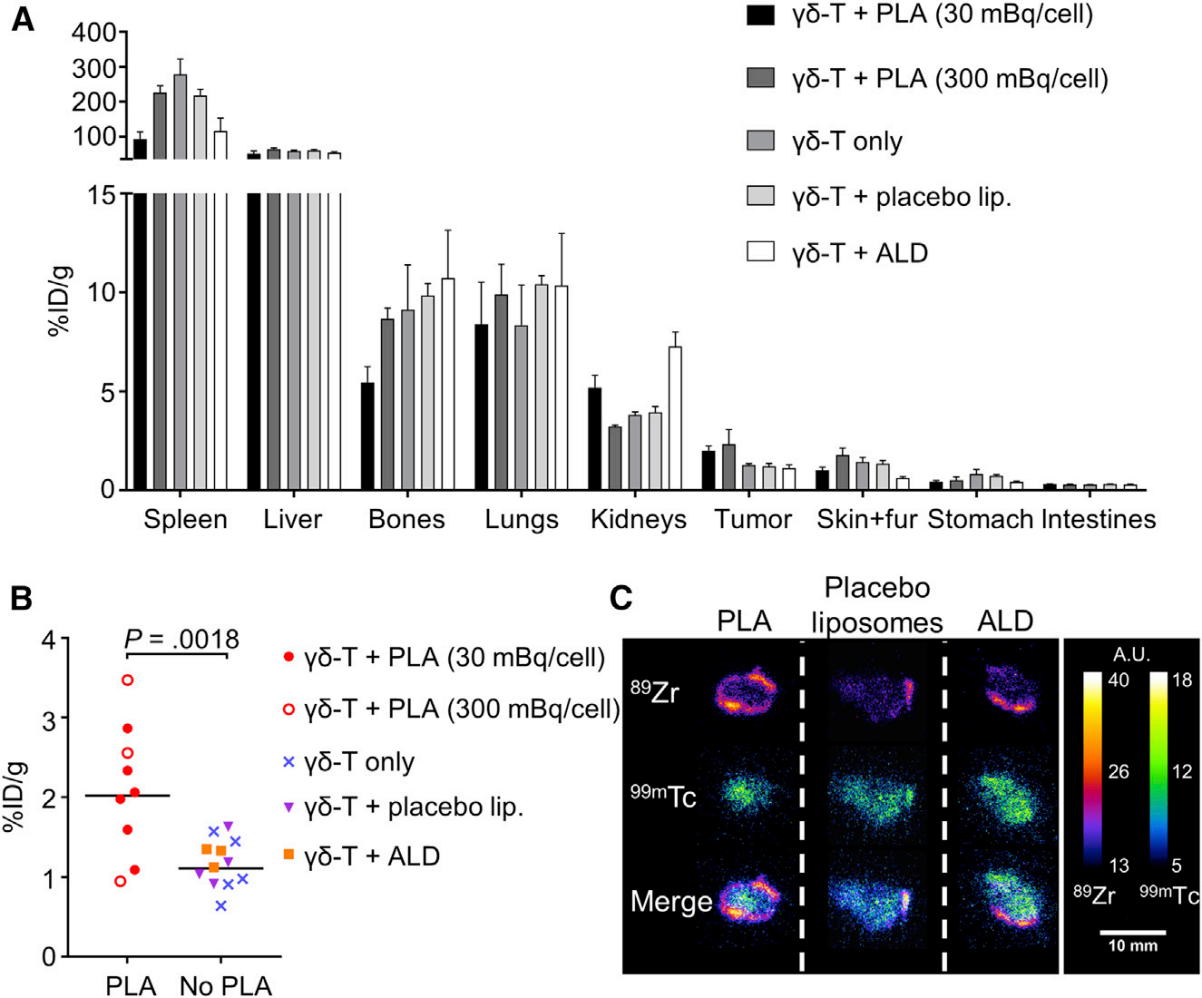
PLA Treatment Increases the Accumulation of γδ-T Cells in Tumors (A) *Ex vivo* bio-distribution of radiolabeled γδ-T cells, 7 days after γδ-T cell administration. Mean ± SEM of ^89^Zr uptake after PLA (N = 6 and 3, respectively), placebo liposomes (N = 4), non-liposomal alendronate (ALD; N = 3), or vehicle (N = 5) treatment. Data are from 3 pooled independent experiments (total N = 21). (B) Comparison of ^89^Zr accumulation in the tumor between PLA (N = 9) and non-PLA (N = 12) treatments (unpaired t test). (C) Artificially colored autoradiographs of tumor sections after PLA, placebo liposomes or non-liposomal alendronate (ALD) treatment. Images are representative of N = 3, 4, and 3 animals per group (scale bar, 10 mm).

*Ex vivo* γ-counting 7 days post-administration of radiolabeled cells revealed a high concentration of ^89^Zr in the spleen (153.5% ± 88.8% injected dose [ID]/g averaged across all groups, N = 24) and liver (58.1% ± 10.6% ID/g, N = 24) in all groups, followed by lung and bone tissue (Figure 4A). Uptake of ^89^Zr in tumors from PLA-treated groups (2.1% ± 0.8% ID/g) was significantly higher than in non-PLA groups (1.2% ± 0.3% ID/g; Figure 4B), suggesting higher γδ-T cell numbers in PLA-treated tumors. Bone uptake of ^89^Zr in PLA-treated groups (6.5% ± 0.8% ID/g, N = 9) was significantly lower than in other groups (10.0% ± 1.1% ID/g, N = 12; p = 0.0238). Uptake in kidneys was significantly higher with killed γδ-T cells than in other treatment groups (Table S3). Uptake in other organs showed no major differences between treatment groups.

**Figure 5:**
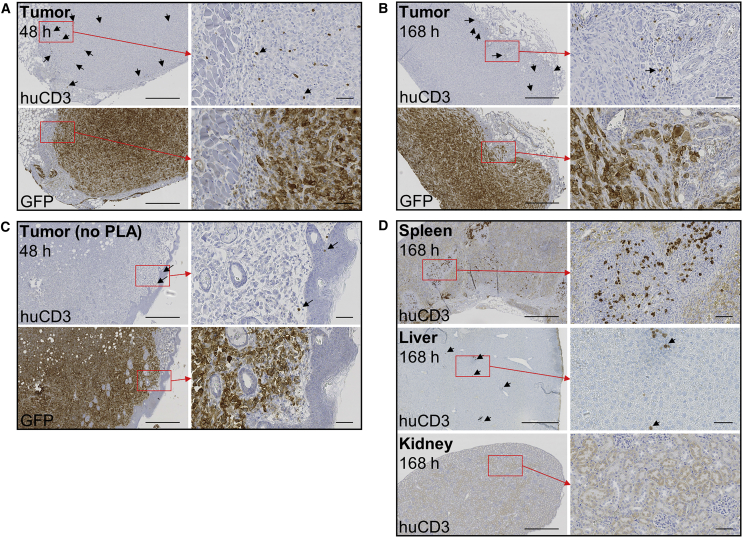
Histology of γδ-T Cells (A–C) Tumor sections 48 hr (A and C) or 7 days (B) after the injection of ^89^Zr-radiolabeled γδ-T cells into mice treated with PLA (A and B) or without PLA (C), stained for human CD3 (γδ-T cells) or GFP (tumor cells). Arrows indicate representative CD3^+^ cells. (D) Spleen, liver, and kidney sections 7 days after the administration of ^89^Zr-radiolabeled γδ-T cells. Sections are representative of N = 2–3 animals per time point. 6× (left) and 30× (right) magnification; scale bars, 500 μm (left) and 50 μm (right).

Tumor section autoradiographs showed a strong signal originating from hNIS-accumulated ^99m^TcO_4_^−^. Autoradiography was repeated after 4 days to allow for the decay of ^99m^Tc and the capture ^89^Zr signal. Sections from PLA-treated animals showed increased ^89^Zr signal compared to non-PLA-treated animals. The ^89^Zr signal was higher in the tumor periphery, whereas the ^99m^Tc signal was uniformly distributed (Figure 4C). γδ-T cell presence in tumors was demonstrated by immunohistochemistry. Human CD3-positive cells (>95% γδ-T cell receptor [TCR]^+^ at the time of administration; Figure S3) were visible in tumors 48 hr and 7 days after injection, both in the periphery and deeper regions (Figures 5A–5C; Figure S7). These cells were also visible in the spleen and liver after 7 days, but not in kidney sections (Figure 5D) or in control tissues of mice not administered γδ-T cells (Figure S8).

## Discussion

[^89^Zr]Zr(oxinate)_4_ synthesis has been reported previously by our group^23^ and others.^24^, ^25^ The temperature-independent labeling efficiency of γδ-T cells with [^89^Zr]Zr(oxinate)_4_ suggests this is a passive process, in line with results from Sato et al.^24^ Sufficient radiotracer retention within cells is important to ensure that the imaging signal reflects labeled cells rather than free radiotracer bio-distribution. We observed an efflux of approximately half of the incorporated ^89^Zr over 1 week *in vitro*, which we believe does not interfere with *in vivo* imaging within this time frame. Uptake of ^89^Zr in the bone can be used to estimate the amount of tracer that leaked from the cells.^25^, ^28^ Retention of [^89^Zr]Zr(oxinate)_4_ is dependent on cell type, and our results are comparable to those observed with dendritic, bone marrow, and chimeric antigen receptor (CAR)-T cells.^23^, ^24^, ^25^ Comparable levels of tracer efflux have been observed from lymphocytes labeled with [^111^In]In(oxinate)_3_,^29^, ^30^ the current gold standard for cell tracking by nuclear imaging.

A radiotracer for cell tracking must not significantly alter the phenotype, survival, proliferation capacity, and functionality of labeled cells. We demonstrated that the effects of [^89^Zr]Zr(oxinate)_4_ on γδ-T cell survival, proliferation capacity, and DNA damage were kept minimal for doses up to 20 mBq/cell but were significant at doses ≥50 mBq/cell. The cytotoxicity of radiolabeled γδ-T cells against the same tumor cells used for *in vivo* experiments was not affected by amounts of [^89^Zr]Zr(oxinate)_4_ of up to 600 mBq/cell, at least within 48 hr of radiolabeling. Cancer cell death was due to the combination of bisphosphonate treatment and γδ-T cells and not to the presence of ^89^Zr. Preserved cytotoxicity after radiolabeling, also recently observed in CAR-T cells by Weist et al.,^25^ is encouraging for the use of [^89^Zr]Zr(oxinate)_4_ as a T cell-tracking agent. However, the therapeutic efficacy of γδ-T cells presumably also relies on their *in vivo* proliferation ability; hence, we suggest that radiolabeling γδ-T cells with [^89^Zr]Zr(oxinate)_4_ should ideally not exceed 20 mBq/cell. This could lead to sensitivity issues on conventional PET scanners. Indeed, our experiments show that *ex vivo* gamma-counting tumors could reveal amounts of ^89^Zr indistinguishable from background in our PET imaging system at day 7. Assuming that a human cell-tracking study would require 37 MBq ^89^Zr^31^ and 10^9^ γδ-T cells,^14^ this would equate to an average of 37 mBq/cell, which we have shown not to be excessively damaging to γδ-T cells. Upcoming developments in PET technology, such as total-body PET,^32^ should reduce the required ^89^Zr activity per cell (by a factor of 40) and overcome these sensitivity issues.

For *in vivo* studies, a xenograft model of human breast cancer in immunocompromised mice^12^, ^33^ was chosen, as mice do not possess a subset of T cells functionally equivalent to human Vγ9Vδ2 T cells.^34^ We tracked γδ-T cells radiolabeled with [^89^Zr]Zr(oxinate)_4_ (30−300 mBq/cell) by PET 1 hr, 48 hr, and 7 days after intravenous injection. We simultaneously used ^99m^TcO_4_^−^ to visualize hNIS-expressing tumors by SPECT. The *in vivo* distribution of ^89^Zr-labeled γδ-T cells over time was similar to that observed in studies of adoptively transferred γδ-T^35^, ^36^ and other T cells.^25^, ^37^, ^38^ ^89^Zr uptake was significantly increased in PLA-treated tumors, suggesting that PLA increases homing of these cells to the tumor site. Accumulation of γδ-T cells at the tumor site 48 hr after administration was also observed by others.^35^ Uptake values for the spleen and tumor determined by image-based quantification are lower than those determined by *ex vivo* bio-distribution. This can be explained by the small size of this organ and significant partial volume effect (spleen) and the liquid or necrotic tumor core that leaked upon dissection.

For instrument sensitivity reasons, some imaging studies were performed with higher doses of ^89^Zr than recommended above. However, the distinctly different distribution pattern observed with killed γδ-T cells suggests that radiolabeling with up to 300 mBq/cell, which preserved cytotoxic functionality *in vitro* over 48 hr, did not impair γδ-T cell trafficking and allowed us to track live cells. Furthermore, previous studies have shown that [^89^Zr]Zr(oxalate)_4_,^28^, ^39^ [^89^Zr]Zr(oxinate)_4_, and lysates from [^89^Zr]Zr(oxinate)_4_-labeled cells^23^ have distinct distribution patterns from intact cells labeled with [^89^Zr]Zr(oxinate)_4_. Cell concentrations during labeling and *in vitro* assays were in the range of 1−5 × 10^6^/mL. In comparison, using *in vitro* ^89^Zr retention values, cell concentrations extrapolated from PET-computed tomography (CT) images in the organs showing the strongest ^89^Zr signal (spleen, liver, and lungs) would be in the range of 0.5−5 × 10^6^ cells/mL. We therefore expect the DNA damage sustained by γδ-T cells, due to both self-irradiation and crossfire, after *in vivo* administration to be comparable to that observed *in vitro*. Considering the strong affinity of the ^89^Zr^4+^ ion for bone,^28^ the relatively low bone accumulation of ^89^Zr indicates limited efflux of weakly chelated ^89^Zr, and it suggests that ^89^Zr is mostly retained by γδ-T cells after injection. The lower accumulation of ^89^Zr in the bones of PLA-treated animals compared to other groups also suggests reduced efflux of ^89^Zr from γδ-T cells after PLA treatment.

Histology confirmed the presence of γδ-T cells in the tumors, spleen, and liver, using the CD3 marker.^40^ Immunohistochemistry and autoradiography suggest that γδ-T cells accumulated mostly at the periphery of the tumor. The small number of cells observed by immunohistochemistry precludes statistical comparison. Furthermore, these techniques can only image the solid portion of the tumor. PET imaging not only allowed visualization of the whole, intact tumors but additionally revealed heterogeneous distributions of ^89^Zr in tumors, which would be challenging to observe by histology. Combined with the non-invasive nature of PET imaging, this further highlights the value of using PET tracers such as [^89^Zr]Zr(oxinate)_4_ for cell tracking. The high uptake of ^89^Zr in the liver and spleen was mirrored by the large numbers of human CD3^+^ cells observed in these tissues, consistent with the bio-distribution of radiolabeled γδ-T cells. In contrast, the apparent absence of CD3^+^ cells in the kidneys, despite higher ^89^Zr uptake than in the tumor, and the fact that the kidney uptake of ^89^Zr was significantly higher in animals administered killed γδ-T cells than in other groups both suggest that the radioactivity detected in the kidneys corresponds to ^89^Zr progressively released from γδ-T cells in other organs. A limitation of directly labeling cells is that the radionuclide can leak out over time and be taken up by adjacent tissue. Although immunohistochemistry demonstrates the presence of the administered γδ-T cells in the tumors, this technique cannot determine whether the ^89^Zr signal originates from the γδ-T cells or from *in situ*-labeled bystander cells.

A critical aspect of this type of cellular immunotherapy is that the therapeutic cells must be activated at the target site and reach the tumor in sufficient numbers. γδ-T cell toxicity toward cancer cells is greatly amplified by bisphosphonates, suggesting a role for γδ-T cells in the anti-cancer properties of bisphosphonates.^41^ Here we sought to increase phosphoantigen expression in tumors by administering PLA, which delivers alendronate to the tumors in an untargeted fashion by virtue of the enhanced permeability and retention (EPR) effect.^42^ Liposomal alendronate proved safer than other bisphosphonates and effective in potentiating γδ-T cell therapy.^12^, ^43^ We have previously shown that the tumor-to-background uptake ratio of PLA increases over time and is significant after 3 days.^44^ Here we observed that PLA administered 4 days in advance significantly increased the amount of ^89^Zr reaching the tumor within 48 hr of radiolabeled γδ-T cell administration. Our results suggest that γδ-T cells home to the tumor within 2 days and remain there for at least 5 days. This was not observed in any other treatment group, demonstrating the importance of encapsulating the aminobisphosphonate in a tumor-targeting vehicle.

Clinical imaging studies of therapeutic T cells with [^111^In]In(oxinate)_3_ have been performed by radiolabeling only a fraction of the total administered T cells,^45^, ^46^, ^47^ although evidence exists that distributing the total activity over a larger number of cells better preserves their proliferative abilities.^48^ Our results suggest that radiolabeling the entire batch of γδ-T cells with [^89^Zr]Zr(oxinate)_4_ might be the preferable option to avoid imaging excessively damaged cells. In two notable studies, γ-scintigraphy revealed T cell uptake in tumors using only 1−3 mBq ^111^In per cell.^46^, ^49^ Considering the increased sensitivity of PET over SPECT and expected improvements in PET technology, clinical imaging of T cell therapies using [^89^Zr]Zr(oxinate)_4_ is a credible prospect.

### Conclusions

This study demonstrates the suitability of [^89^Zr]Zr(oxinate)_4_ as a PET tracer to track γδ-T cells *in vivo*, while previous work has shown the therapeutic efficacy of γδ-T cells in combination with PLA.^12^, ^43^ These objectives achieved, [^89^Zr]Zr(oxinate)_4_ can now be applied to answer fundamental questions in the preclinical and clinical development of γδ-T cell therapies, e.g., whether the accumulation of γδ-T cells at the tumor site or their distribution within the tumor correlates with therapeutic efficacy. Due to numerous molecular and cellular differences, the distribution of human γδ-T cells in an immunocompromised mouse model cannot fully predict their behavior in a human host. However, the results of this proof-of-principle study can be used to design a clinical trial that will answer the question of the distribution of γδ-T cells in humans after adoptive transfer.

Our results have implications for clinical translation, and they suggest using liposomal aminobisphosphonates as adjuncts to γδ-T cell therapy. In the context of clinical protocols involving repeated infusions of γδ-T cells,^15^ one can envisage the use of ^89^Zr-labeled cells for the first infusion, followed by PET imaging 24–72 hr later. The number of cells trafficking to the tumor sites would then be used to decide whether to pursue with additional treatment cycles. Cell radiolabeling with [^89^Zr]Zr(oxinate)_4_ is clinically translatable without significant methodological modifications, and the high similarity of [^89^Zr]Zr(oxinate)_4_ to the well-established [^111^In]In(oxinate)_3_ should facilitate regulatory approval. Our results support that T cell labeling with [^89^Zr]Zr(oxinate)_4_ is a realistic option for human studies and will benefit the development of cellular immunotherapy.

## Materials and Methods

### Experiment Approval

Animals experiments were approved by the UK Home Office under The Animals (Scientific Procedures) Act (1986), PPL reference 7008879 (Protocol 6), with local approval from King’s College London Research Ethics Committee (KCL-REC). Experiments using human T cells received approval from KCL-REC (Study Reference HR-16/17-3746). All donors provided written, informed consent.

### Reagents, Animals, and Cells

Unless otherwise indicated, reagents were purchased from Sigma-Aldrich and Merck. Female SCID/beige (CB17.Cg-Prkdc^scid^Lyst^bg-J^/Crl) and Nod scid gamma (NSG) (NOD.Cg-Prkdc^scid^ Il2rg^tm1WjI^/SzJ) mice (18−25 g, 10−20 weeks old) were obtained from Charles River (UK). γδ-T cells were obtained as described previously,^12^ using zoledronate (Novartis) and interleukin-2 (IL-2) (Novartis). Full details are provided in the Supplemental Materials and Methods. Population purity was assessed by flow cytometry (BD FACSCalibur), using pan-γδ TCR (IMMU510, Beckman Coulter B49175) and anti-CD3 (OKT3, BioLegend 317307) monoclonal antibodies. Data were analyzed using Flowing version (v.)2.5.1 (http://flowingsoftware.btk.fi). Only batches with ≥80% γδ-positive CD3^+^ cells were used for further experiments (≥95% for *in vivo* experiments). MDA-MB-231.hNIS-GFP cells^27^ were grown in DMEM supplemented with 10% fetal bovine serum (FBS), penicillin, streptomycin, and l-glutamine (2 mM), and they were tested for mycoplasma contamination (e-Myco PCR detection kit, Bulldog Bio).

### PET Tracer Synthesis

[^89^Zr]Zr(oxinate)_4_ was synthesized as previously described.^23^ Full details are provided in the Supplemental Materials and Methods.

### Cell Labeling

γδ-T cells expanded *in vitro*^12^ were washed with PBS (Ca^2+^/Mg^2+^ free) and re-suspended at 5 × 10^6^/mL in PBS at RT. [^89^Zr]Zr(oxinate)_4_ (6−600 mBq/cell) in aqueous DMSO was added to the cell suspension, keeping DMSO concentrations ≤0.7%. Neutralized [^89^Zr]Zr(oxalate)_4_ with an equivalent amount of DMSO was used as a control. After 10−30 min of incubation, cells were pelleted and the supernatants kept aside. The cells were washed with PBS, centrifuged, and the washings combined with the previous supernatants. The cells were suspended in growth medium or PBS for further experiments. Viability was assessed using the trypan blue dye exclusion method. Radioactivity in re-suspended cells and combined supernatants was measured in a gamma-counter. Cell-labeling efficiency (LE[%]) was calculated as follows.

$$LE\left(\%\right)=\frac{\text{activity}\;\text{of}\;\text{cell}\;\text{fraction}}{\text{activity}\;\text{of}\;\text{cell}\;\text{fraction}\;+\;\text{activity}\;\text{of}\;\text{combined}\;\text{supernatants}}$$

For radiotracer retention and cell proliferation studies, radiolabeled (or vehicle-treated) γδ-T cells were cultured as described above, and they were analyzed at various time points for viability (using trypan blue), determination of cell-associated radioactivity (by γ-counting), and cell death (by flow cytometry using propidium iodide [PI]; Thermo Scientific). Further details are provided in the Supplemental Materials and Methods.

### Cancer Cell-Killing Assay

MDA-MB-231.hNIS-GFP cells seeded in a 96-well plate at 10^4^ cells/well and incubated overnight were treated with 3 μM zoledronate or vehicle for 24 hr. The cells were washed and the medium was replaced with γδ-T cells in growth medium. As a control for radiolabeled γδ-T cells, an equal amount of ^89^Zr in medium was added to some wells. After 48 hr, γδ-T cells were removed by washing with PBS, and cancer cell viability was evaluated using the alamarBlue assay (Thermo Scientific), reading plates in a GloMax (Promega) reader (530 nm excitation and 590 nm emission filters).

### Determination of DNA Double-Strand Breaks

Radiolabeled γδ-T cells in medium were seeded onto poly-l-lysine-coated coverslips and incubated for 1 hr. After centrifugation and gentle rinsing with PBS, the cells were fixed and permeabilized with 3.7% formalin, 0.5% Triton X-100, and 0.5% IGEPAL CA-630 in PBS, then blocked with 2% BSA and 1% goat serum. γH2AX foci were detected with an anti-γH2AX (Ser139) mouse monoclonal antibody (mAb) (1:1,600; JBW301, Merck 05-636) and goat anti-mouse AF488-immunoglobulin G (IgG) (1:500; Jackson ImmunoResearch Laboratories 115-545-062). Nuclei were detected with Hoechst 33342. Images were acquired on a TCS SP5 II confocal microscope (Leica) with a 100×/1.40 HCX PL Apochromat objective (Leica) and Leica Application Suite Advanced Fluorescence (LAS-AF) control software. Ten sections (0.4-μm thickness) were imaged. At least 30 nuclei/slide were imaged (2 slides/treatment). Maximal intensity projections of z stacks were made using ImageJ v.1.51p (https://imagej.nih.gov/ij/). Nuclei and γH2AX foci were counted using CellProfiler v.2.2.0 (http://cellprofiler.org), calculating average numbers of γH2AX foci per nucleus in each image. Full details are provided in the Supplemental Materials and Methods.

### Animals, Tumor Model, and Tumor Sensitization with Liposomal Alendronate

Approximately 1.5 × 10^6^ MDA-MB-231.hNIS-GFP cells were injected subcutaneously in the mammary fat pad between the fourth and fifth nipples in the left flank; tumors were grown over 3 weeks. Animals were randomly assigned to experimental groups, and investigators were not blinded to cohort allocation when assessing outcomes. Cohort sizes were chosen based on prior experience,^44^, ^50^ in compliance with local regulations concerning animal experiments. Liposomal formulations were prepared at Shaare Zedek MC as previously described.^13^ Alendronate-loaded liposomes (PLA) contained 1.5−5.4 mg/mL alendronate and 36−40 μmol/mL phospholipids. Placebo liposomes contained 20−50 μmol/mL phospholipids. PLA was co-injected with placebo liposomes for a total dose of 5 mg/kg alendronate and 4 μmol phospholipids per mouse in PLA-treated animals. Placebo-treated animals received empty liposomes corresponding to 4 μmol phospholipids per mouse. Another control group received 5 mg/kg alendronate (ALD). Formulations were injected intravenously (i.v.) 4 days before the administration of radiolabeled γδ-T cells.

### *In Vivo* PET and SPECT Imaging of γδ-T Cells, Tumors, and PLA

^89^Zr-radiolabeled γδ-T cells (10^7^ cells/animal in 100 μL, 0.3−3 MBq ^89^Zr, single γδ-T donor per experiment) were injected i.v. at t = 0 hr and imaged by PET/CT within 30 min. PET/CT imaging was performed for 30−240 min (as indicated) on a nanoScan PET-CT scanner (Mediso). For tumor imaging, 100 μL ^99m^TcO_4_^−^ (15−25 MBq) in saline was injected i.v., and SPECT-CT was performed 40 min thereafter in a NanoSPECT/CT scanner (Mediso; 1-mm collimators, 30-min scan). PET-CT and SPECT-CT were repeated at t = 48 and 168 hr. For PLA imaging by SPECT-CT, PLA was radiolabeled with [^111^In]In(oxinate)_3_ and administered i.v. (7 MBq ^111^In/mouse) to NSG mice. PET- and SPECT-CT datasets were reconstructed using a Monte Carlo-based full-3D iterative algorithm (Tera-Tomo, Mediso). Images were co-registered and analyzed using VivoQuant v.2.50 (Invicro). Regions of interest (ROIs) were delineated for PET activity quantification in specific organs. Uptake in each ROI was expressed as a percentage of injected dose per volume (% ID/mL).

### *Ex Vivo* Bio-distribution Studies

Mice from imaging studies were used for bio-distribution studies on day 2 or 7. After culling, organs were dissected, weighed, and γ-counted together with standards prepared from a sample of injected material. The percentage of injected dose per gram (% ID/g) of tissue was calculated. Organs were cryopreserved in optimal cutting temperature (OCT) compound (VWR) for autoradiography and/or formalin fixed and paraffin embedded (FFPE) for histologic analysis.

### Autoradiography

Cryopreserved tissues were cut (50 μm), mounted on poly-l-lysine-coated slides (VWR), fixed in 4% paraformaldehyde (PFA), mounted in Mowiol, and exposed to a storage phosphor screen for 20 min at 3 hr post-dissection to obtain the ^99m^Tc signal, then for 48 hr at 4 days post-dissection to obtain the ^89^Zr signal. The storage phosphor screen was read using a Cyclone Plus imager (PerkinElmer), and images were processed with ImageJ.

### Immunohistochemistry

Briefly, FFPE organ blocks were sliced and stained using a Discovery XT system (Ventana Medical Systems) using the DAB Map detection kit (Ventana 760-124). For pre-treatment, CC1 (Ventana 950-124) was used. Sections were stained with anti-GFP (1/1,000; Abcam ab290, UK) or anti-CD3 (LN10, Leica CD3-565-L-CE) primary antibodies, followed by biotinylated anti-rabbit or anti-mouse IgG (1/200; Dako) secondary antibodies, as appropriate. Full details are provided in the Supplemental Materials and Methods.

### Statistics

Independent experiments were performed on different days with γδ-T cell batches from different donors. Data were plotted using Prism v.7.01 (GraphPad). Differences between 2 groups were evaluated by Student’s two-tailed t test. To account for repeated measurements in a same animal or cell batch and multiple treatments tested on a same cell batch, analysis was performed using 2-way repeated-measures ANOVA in GraphPad Prism or a repeated-measures Mixed Model (MM)^51^ in InVivoStat v.3.7 (http://invivostat.co.uk/), as indicated. Dunnett’s post hoc test was applied for comparisons back to a control group, or Bonferroni correction for multiple pairwise comparisons, unless otherwise specified. Exact significance values are reported in each figure.

## Author Contributions

Conceptualization, F.M., L.L., G.O.F., and R.T.M.d.R.; Methodology, F.M., L.L., A.G., G.O.F., and R.T.M.d.R.; Investigation, F.M., L.L., A.V., B.D., A.C.P.-P., and R.T.M.d.R.; Writing – Original Draft, F.M.; Writing – Review and Editing, F.M., L.L., A.G., J.M., P.J.B., G.O.F., and R.T.M.d.R.; Funding Acquisition, G.O.F., R.T.M.d.R., P.J.B., and J.M.; Resources, F.M., L.L., A.G., H.S., and R.T.M.d.R.; Supervision, R.T.M.d.R., G.O.F., and P.J.B.

## Conflicts of Interest

J.M. is chief scientific officer of Leucid Bio, a company dedicated to the commercial development of CAR-T cells for solid tumors. The authors declare no other potential conflicts of interest.

## Supplemental Information

Document S1. Figures S1–S8, Tables S1–S3, and Supplemental Materials and Methods

Document S2. Article plus Supplemental Information
