# Supplementary material for: In Vivo PET Tracking of 89Zr-Labeled Vγ9Vδ2 T Cells to Mouse Xenograft Breast Tumors Activated with Liposomal Alendronate
Source: Mol Ther. 2018 Oct 16;27(1):219–29. doi: 10.1016/j.ymthe.2018.10.006 (PMC6318719; doi:10.1016/j.ymthe.2018.10.006)
Supplement: Document S1. Figures S1–S8, Tables S1–S3, and Supplemental Materials and Methods [file mmc1.pdf]

YMTHE, Volume *27*

Supplemental Information

In Vivo PET Tracking of ^{89}Zr-Labeled Vγ9Vδ2 T Cells to Mouse Xenograft Breast Tumors Activated with Liposomal Alendronate

Francis Man, Lindsay Lim, Alessia Volpe, Alberto Gabizon, Hilary Shmeeda, Benjamin Draper, Ana C. Parente-Pereira, John Maher, Philip J. Blower, Gilbert O. Fruhwirth, and Rafael T.M. de Rosales

Supplemental Figures

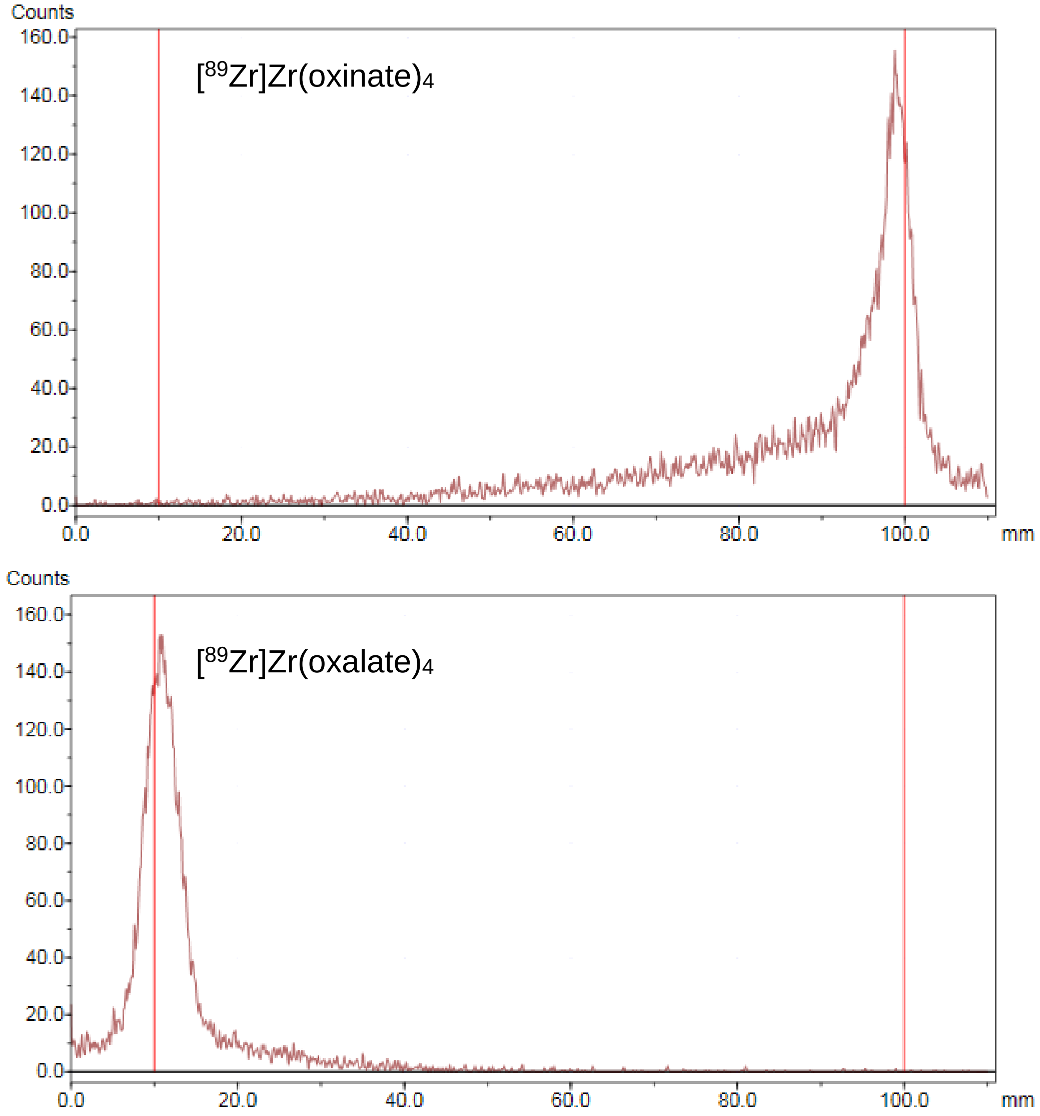

Figure S1. Representative radioTLC chromatograms of [^{89}Zr]Zr(oxinate)$_4$ (top, $R_F \approx 1$) and [^{89}Zr]Zr(oxalate)$_4$ (bottom, $R_F \approx 0$).

A solution containing $^{89}Zr^{4+}$ in 1 M oxalic acid was diluted with H_2O, neutralized with 1 M Na_2CO_3 and an aliquot spotted onto an ITLC-SG plate, dried and run in 100 % EtOAc. To the neutralized solution, 8-hydroxyquinoline (oxine) in $CHCl_3$ was added and the mixture was vortexed. The organic layer was extracted and dried at 60 °C. The residue was dissolved in DMSO, spotted onto an ITLC-SG plate, dried and run in 100 % EtOAc. The ITLC plates were read on a linear radioTLC scanner equipped with a β^+ probe.

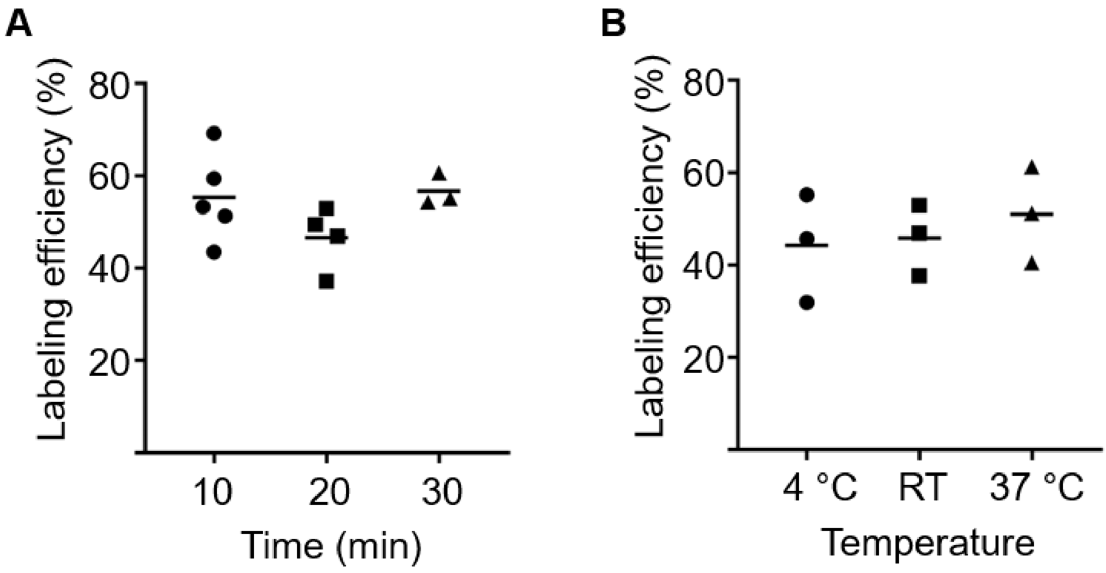

Figure S2. *In vitro* labeling of γδ-T cells with [^{89}Zr]Zr(oxinate)$_4$.

Labeling efficiencies of γδ-T cells incubated with [^{89}Zr]Zr(oxinate)$_4$ (69.0 ± 7.9 mBq/cell) at room temperature for varying amounts of time (**A**), or for 20 min at varying temperatures (**B**). Bars represent the average of N = 3-5 individual experiments, each dot within a group representing cells from a different donor.

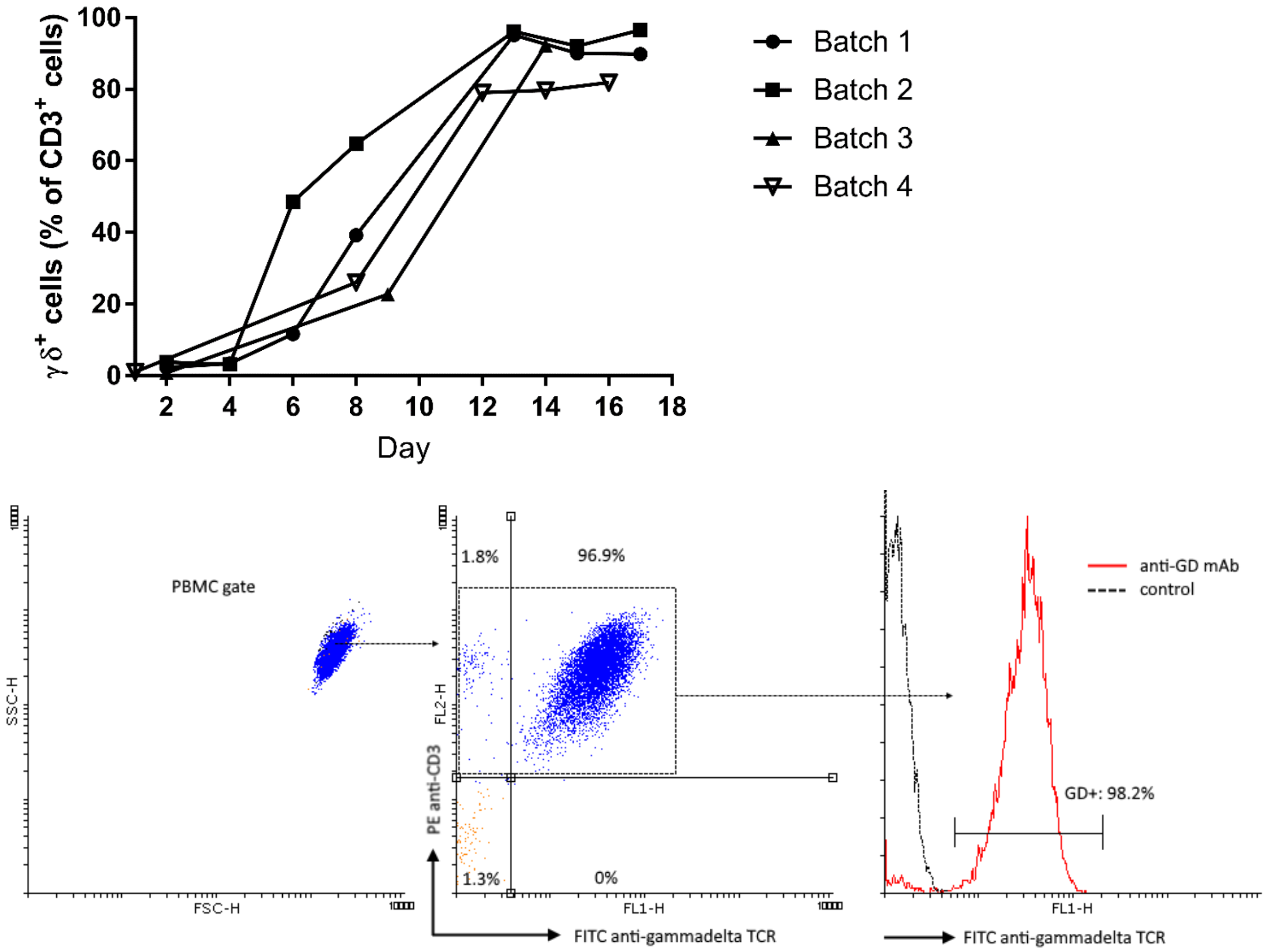

Figure S3. Population purity of γδ-T cells during *in vitro* expansion and flow cytometry analysis.

Top: expansion of the γδ-T cell population from PBMCs isolated from 4 different donors. *Bottom:* gating strategy and example flow cytometry plot of *in vitro* expanded human γδ-T cells on day 14 after isolation. Human PBMCs were isolated by density centrifugation, resuspended in RPMI + 10 % human serum and treated with 3.7 µM zoledronate on the first day and with 100 IU IL-2 every 2-3 days. Aliquots of cultured cells were stained with a FITC-conjugated anti-pan-γδ TCR mAb (IMMU510) and a PE-conjugated anti-CD3 antibody (OKT3) and analyzed by flow cytometry. PBMCs were gated by forward/side scatter and the percentage of γδ-positive events amongst $CD3^+$ events was calculated.

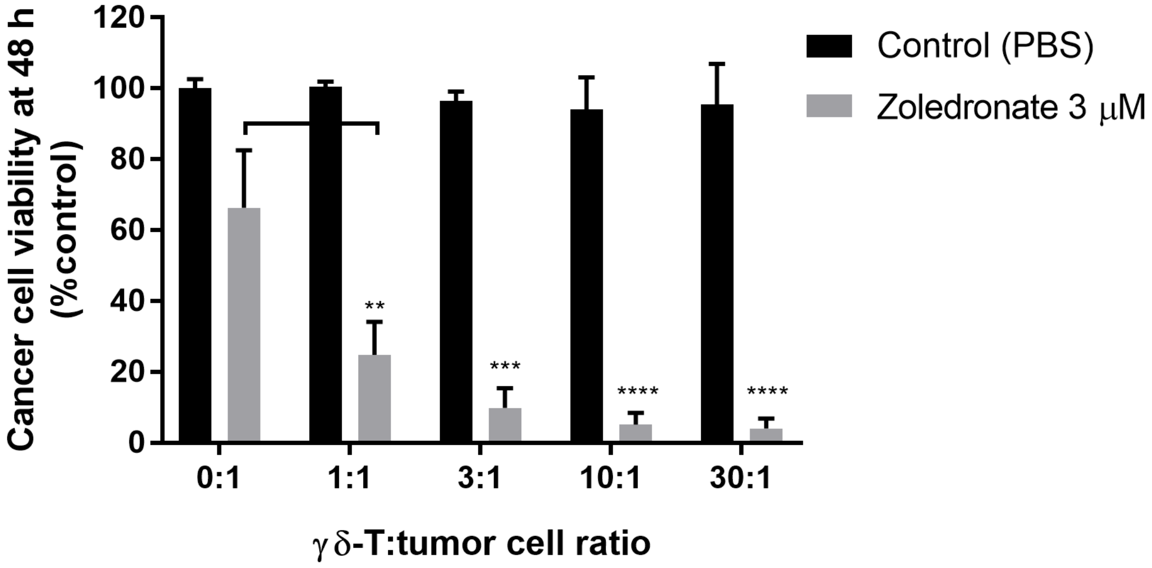

Figure S4. γδ-T cytotoxicity with or without treatment of cancer cells with zoledronate.

MDA-MD-231.hNIS-GFP breast cancer cells were grown to confluence in a 96-well plate (10^4 cells seeded per well) and treated with 3 μM zoledronate or vehicle for 24 h. The medium was then replaced and increasing amounts of γδ-T cells were added for 48 h. Cell viability was measured using the alamarBlue™ assay. Viability is expressed as the percentage of control (cancer cells treated with PBS only, without γδ-T cells). Mean ± SEM of $N = 4$ independent experiments with measurements performed in triplicate. **$P = 0.0025$, ***$P = 0.0002$, ****$P < 0.0001$ *vs*. "Zoledronate 3 μM 0:1" (2-way repeated-measures ANOVA with Dunnett's correction for multiple comparisons).

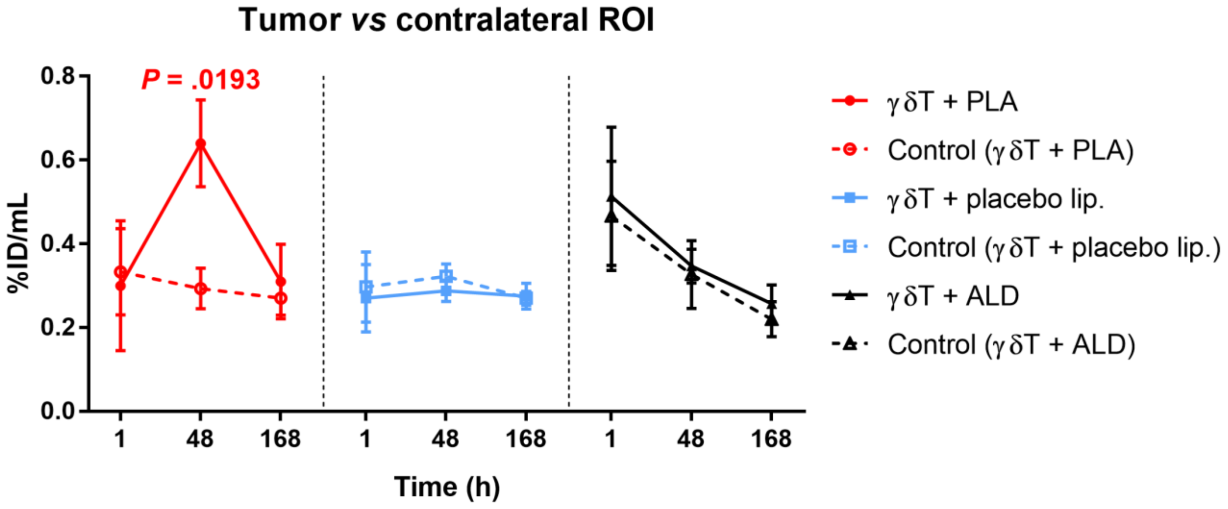

Figure S5. Image-based quantification control**.**

To determine background values of the PET signal in PET-CT imaging studies, a region-of-interest of similar size to that of the tumor was identified by CT imaging and drawn in a contralateral area of each animal, and the amount of ^{89}Zr, expressed as %ID/mL, was determined. Mean ± SEM of N = 3-4 animals per group. Solid lines represent the amounts of ^{89}Zr in the tumors, dashed lines represent the amount of ^{89}Zr in contralateral regions. Repeated-measures MM analysis within PLA group, only significant P values ($P < .05$ *vs*. control group) are reported.

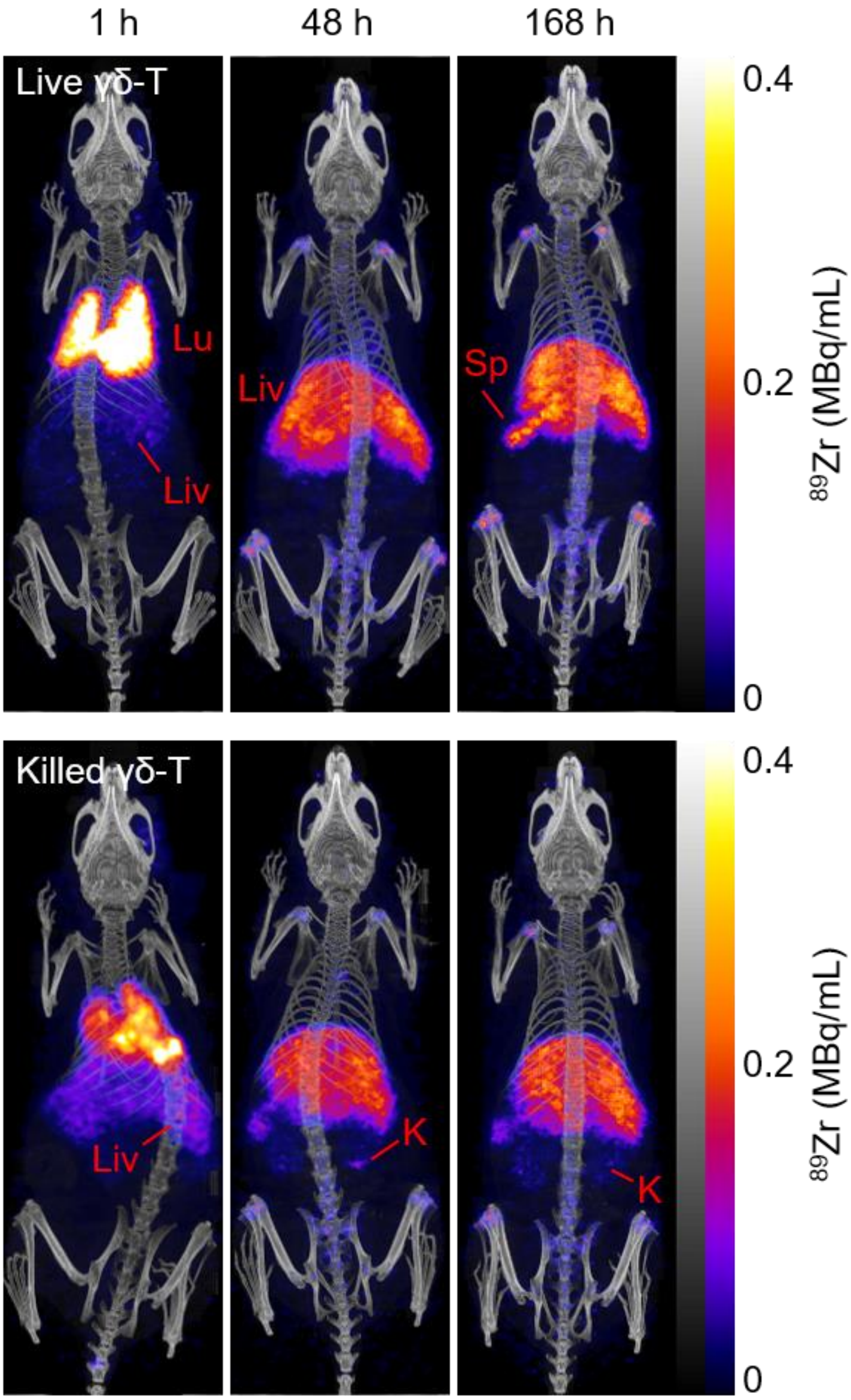

Figure S6. PET/CT imaging of live vs killed ^{89}Zr-labeled γδ-T cells.

Representative maximal intensity projections of PET/CT images of live (top) vs killed (bottom) γδ-T cells radiolabeled with [^{89}Zr]Zr(oxinate)$_4$ (30 mBq/cell) at 1 h, 48 h and 168 h post-injection. Killed γδ-T cells were obtained by subjecting them (after radiolabeling) to 2 freeze-thaw cycles (30 min at −20 °C) before i.v. injection. Lu: lungs; Liv: liver; Sp: spleen; K: kidney.

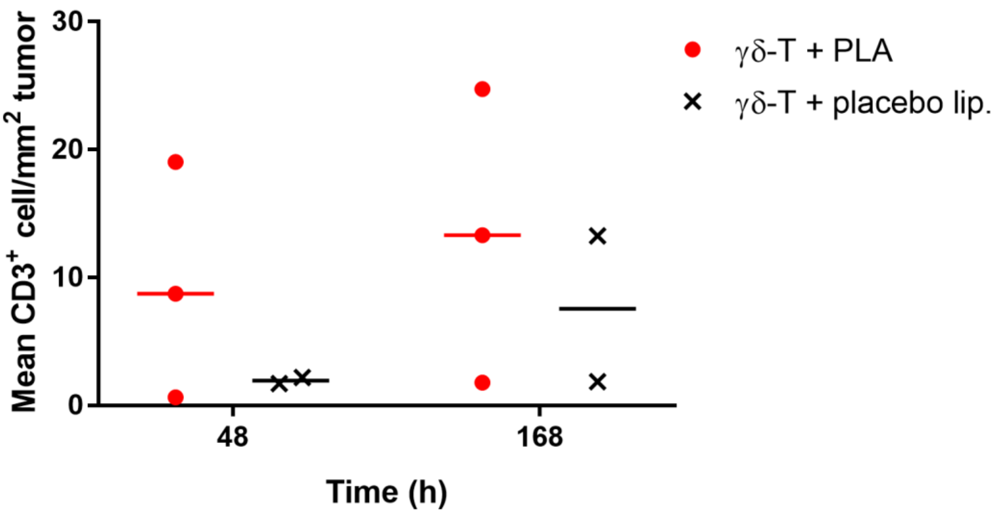

Figure S7. Histology of γδ-T cells (quantification) in tumors.

CD3-positive cells were counted in paraffin-embedded tumor sections from animals (1 tumor/animal) treated with PLA or placebo liposomes, 48 h or 168 h after intravenous administration of ^{89}Zr-labeled γδ-T cells. The number of cells in each section was divided by the total area of the section. Each point represents the average of 3 sections from the same tumor, bars represent the median of 2 or 3 tumors per group.

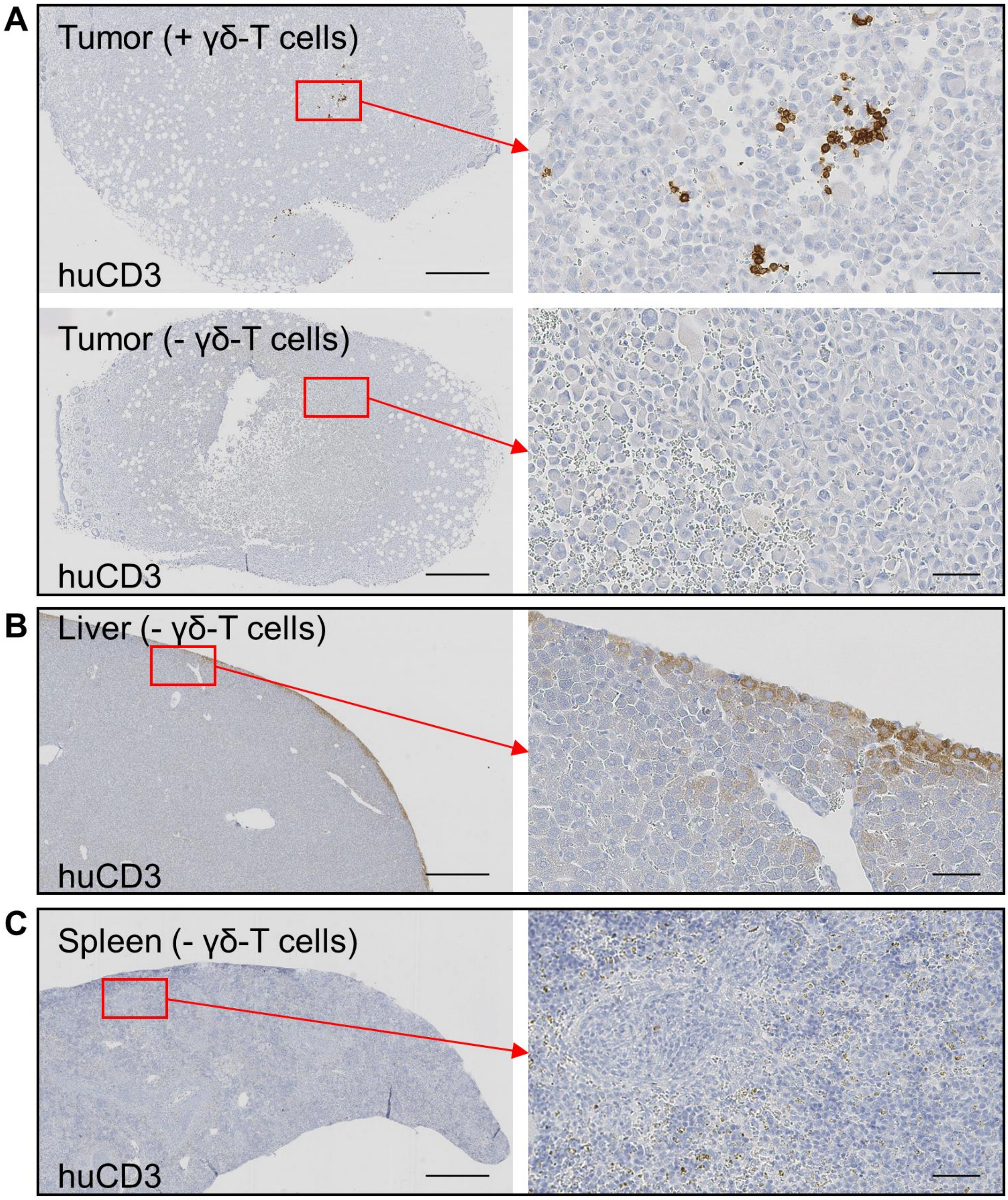

Figure S8. Histology of γδ-T cells (controls).

A Representative slices of MDA-MB-231.hNIS-GFP tumors grown on the same SCID/beige mouse (not administered human γδ-T cells). The animal was euthanized and the tumors surgically removed. The tumor on the top was then injected *ex vivo* with human γδ-T cells, and both tumors were formalin-fixed and embedded in paraffin. **B,C** Representative slices of liver (**B**) and spleen (**C**) sections from control SCID/beige mice (not administered human γδ-T cells). Sections were stained for human CD3. 4/30× magnification; scale bars = 500 µm (left) or 50 µm (right).

Supplemental Tables

Table S1. Image-based quantification of ^{111}In uptake in the tumor.

Female NSG mice bearing MDA-MB-231.hNIS-GFP tumors were administered ^{111}In-labeled PEGylated liposomal alendronate intravenously and imaged by SPECT/CT after 24 h and 72 h.

**Time after ^{111}In-PLA administration**	**24 h**	**72 h**
	**^{111}In uptake in tumor, mean ± SD**	
**% injected dose (%ID)**	4.7 ± 1.7	10.9 ± 2.7
**% injected dose per unit volume (%ID/mL)**	8.7 ± 1.2	12.9 ± 0.8

Table S2. Image-based PET quantification data of ^{89}Zr-labeled γδ-T cells.

Uptake of ^{89}Zr at the tumor site was higher in PLA-treated animals 48 h after administration of radiolabeled γδ-T cells.

Treatment	γδ-T + PLA			γδ-T + placebo liposomes			γδ-T + ALD		
Number of animals	3			3			3		
**Day post-injection**	**1**	**3**	**7**	**1**	**3**	**7**	**1**	**3**	**7**
**Organ**	**%ID/mL (mean ± SD)**								
Stomach	3.74 ± 0.90	11.27 ± 1.90	8.85 ± 3.32	7.44 ± 8.04	11.18 ± 1.25	11.37 ± 4.27	7.77 ± 4.75	12.50 ± 0.14	9.94 ± 3.14
**Tumor**	0.30 ± 0.27	**0.64 ± 0.18**	0.31 ± 0.16	0.27 ± 0.14	**0.29 ± 0.05**	0.27 ± 0.06	0.51 ± 0.29	**0.35 ± 0.07**	0.26 ± 0.08
Thyroid + salivary glands	0.59 ± 0.26	0.76 ± 0.04	0.91 ± 0.50	0.76 ± 0.30	0.94 ± 0.23	0.94 ± 0.26	0.91 ± 0.10	0.81 ± 0.28	0.91 ± 0.20
Kidney	3.18 ± 1.54	3.45 ± 0.49	3.91 ± 0.37	3.34 ± 1.45	4.38 ± 0.21	5.39 ± 2.41	5.49 ± 0.76	4.29 ± 0.34	4.86 ± 0.64
Bone	1.38 ± 0.99	5.75 ± 0.87	8.83 ± 0.90	2.51 ± 1.46	8.23 ± 0.88	11.32 ± 2.29	3.07 ± 1.60	7.80 ± 0.71	11.17 ± 3.34
Muscle	0.25 ± 0.16	0.41 ± 0.03	0.37 ± 0.11	0.32 ± 0.12	0.51 ± 0.05	0.39 ± 0.26	0.42 ± 0.10	0.33 ± 0.05	0.38 ± 0.08
Lung	42.33 ± 22.84	5.22 ± 1.07	5.21 ± 0.41	33.33 ± 26.88	5.30 ± 1.05	6.04 ± 0.45	29.64 ± 19.01	5.06 ± 0.50	5.48 ± 0.49
Liver	22.87 ± 10.88	40.66 ± 3.82	44.75 ± 0.62	29.10 ± 10.00	41.19 ± 5.47	48.37 ± 4.12	40.41 ± 3.55	43.48 ± 5.04	48.00 ± 4.25
Spleen	14.45 ± 8.42	30.08 ± 5.28	34.51 ± 4.49	16.42 ± 7.42	30.59 ± 1.32	40.54 ± 3.91	24.90 ± 6.97	32.17 ± 6.20	42.07 ± 12.22
Tumor-contralateral ROI	0.33 ± 0.18	0.29 ± 0.08	0.27 ± 0.07	0.30 ± 0.14	0.32 ± 0.06	0.27 ± 0.03	0.47 ± 0.23	0.33 ± 0.14	0.22 ± 0.07

Table S3. *Ex vivo* bio-distribution data of ^{89}Zr after administration of ^{89}Zr-labeled γδ-T cells.

Uptake of ^{89}Zr at the tumor site was higher in PLA-treated animals 7 days after administration of radiolabeled γδ-T cells.

Group	**A**	**B**	**C**	**D**	**E**	**F**
Treatment	γδ-T + PLA	γδ-T + PLA	γδ-T only	γδ-T + placebo liposomes	γδ-T + ALD	γδ-T (killed)
^{89}Zr per cell (mBq)	30	300	30	300	300	30
Number of animals	6	3	5	4	3	3
**Organ**	**%ID/g (mean ± SD) 7 days post-injection**					
Blood	0.87 ± 0.83	2.07 ± 2.02	0.55 ± 0.38	1.66 ± 0.75	1.91 ± 0.87	0.24 ± 0.04
Bone	5.45 ± 1.95	8.66 ± 0.95	10.72 ± 5.42	9.85 ± 1.20	9.12 ± 3.92	4.55 ± 2.73
Heart	1.19 ± 0.41	1.61 ± 0.17	1.14 ± 0.52	1.53 ± 0.15	2.6 ± 1.54	0.50 ± 0.29
Intestines	0.31 ± 0.04	0.30 ± 0.02	0.29 ± 0.07	0.31 ± 0.02	0.28 ± 0.01	0.27 ± 0.08
Kidney	5.19 ± 1.51	3.22 ± 0.12	7.26 ± 1.64	3.94 ± 0.57	3.81 ± 0.24	11.91 ± 1.50[a]
Liver	51.65 ± 17.71	64.17 ± 5.73	54.62 ± 4.72	59.85 ± 7.15	58.96 ± 4.49	67.71 ± 8.99
Lung	8.37 ± 5.24	9.89 ± 2.65	10.36 ± 5.87	10.41 ± 0.87	8.34 ± 3.51	4.53 ± 2.61
Muscle	0.30 ± 0.07	0.96 ± 0.37	0.23 ± 0.05	0.73 ± 0.25	0.60 ± 0.37	0.16 ± 0.03
Thyroid + salivary glands	0.92 ± 0.23	1.12 ± 0.07	0.69 ± 0.07	1.30 ± 0.18	1.15 ± 0.17	0.55 ± 0.05
Skin + fur	1.01 ± 0.37	1.78 ± 0.61	0.60 ± 0.18	1.34 ± 0.31	1.43 ± 0.41	0.52 ± 0.14
Spleen	92.80 ± 50.96	226.09 ± 33.98	115.56 ± 84.27	217.92 ± 35.08	278.07 ± 77.85	55.40 ± 15.67
Stomach	0.42 ± 0.17	0.51 ± 0.28	0.41 ± 0.13	0.72 ± 0.11	0.81 ± 0.40	0.41 ± 0.21
**Tumor**	**1.99 ± 0.61**	**2.32 ± 1.28**	1.11 ± 0.39	1.19 ± 0.31	1.27 ± 0.13	0.98 ± 0.18

[a]$p < 0.001$ *vs*. all other groups (1-way ANOVA with Tukey's test for multiple comparisons).

Supplemental Methods

Synthesis of [^{89}Zr]Zr(oxinate)$_4$

No-carrier-added ^{89}Zr (produced at the BV Cyclotron, VU Amsterdam, NL) was purchased from PerkinElmer as H_4[^{89}Zr(oxalate)$_4$] in 1 M oxalic acid. The acidic ^{89}Zr solution (5−100 MBq) was transferred to a 1.5 mL plastic vial, diluted to 150 µL with Chelex®-treated water and gradually adjusted to pH 7.5−8 (measured with pH strips) with 1 M sodium carbonate. The volume was then adjusted to 450 µL with Chelex®-treated water and 50 µL of a 10 mg/mL solution of 8-hydroxyquinoline in chloroform was added. The mixture was vortexed for 5 min, a further 450 µL of chloroform were added and the mixture vortexed for 10 min. The organic phase was extracted into a conical glass vial and dried at 60 °C under a flow of nitrogen gas. The residue was dissolved in aqueous dimethyl sulfoxide (DMSO, 30 ± 10 % in water) for further use. Radiochemical yield was defined by the amount of radioactivity present in the dried organic extract divided by the starting amount of radioactivity. Radioactivity in samples was measured with a CRC-25R dose calibrator (Capintec). Product formation was confirmed by radioTLC on instant thin-layer chromatography (ITLC)-SG paper (Macherey-Nagel) using 100 % ethyl acetate as the mobile phase. ITLC plates were read using a Mini-Scan™ radioTLC linear scanner (LabLogic Systems) equipped with a β^+ probe (LabLogic B-FC-3600). Radiochemical purity of the final product was determined as the radioactivity associated with the [^{89}Zr]Zr(oxinate)$_4$ peak divided by the total detected radioactivity on the chromatogram.

Isolation and *in vitro e*xpansion of Vγ9Vδ2 T cells

Peripheral blood was collected from healthy, male and female donors aged 22-45. Citrate-anticoagulated blood (20−30 mL) was layered over 15 mL of Ficoll-Paque Plus (GE Healthcare) and centrifuged for 30 min at 750 rcf. The buffy coat containing peripheral blood mononuclear cells (PBMCs) was extracted, diluted with PBS and centrifuged for 10 min at 200 rcf. The supernatant was discarded and the pellet re-suspended in PBS, then centrifuged for 10 min at 550 rcf. PBMCs were then re-suspended at 3×10^6/mL in growth medium (RPMI-1640 supplemented with 10 % human AB serum, 100 U/mL penicillin, 0.1 mg/mL streptomycin and 2 mM L-alanyl-L-glutamine (Glutamax; Gibco)). On the first day, 3.7 µM zoledronic acid (Novartis) and 100 IU/mL IL-2 (Novartis) were added, and the cells incubated at 37 °C in a 5 % CO_2 atmosphere. IL-2 (100 IU/mL) and fresh medium were then added every 2-3 days. γδ-T cells were used on day 13-14 after isolation. Population purity was assessed by flow cytometry (BD FACSCalibur), using a FITC-conjugated pan-γδ TCR monoclonal antibody (mAb) (clone IMMU510; Beckman Coulter) and a PE-conjugated anti-CD3 mAb (clone OKT3; BioLegend). PBMCs were gated by forward/side scatter and a minimum of 10,000 events in the PBMC gate were analyzed.

Radiotracer retention and cell proliferation

Approximately 2.5×10^6 radiolabeled (or vehicle-treated) γδ-T cells were re-suspended in growth medium, seeded in 6-well plates and cultured as described above, with fresh medium and IL-2 added every 2 days. At t = 0, 24, 48, 96, 144 and 192 h, the cells were gently re-suspended and 200−300 µL aliquots were removed for analysis. The number of live cells was counted using Trypan Blue. Cells were pelleted and washed with PBS for γ-counting as described above to determine the percentage of cell-associated radioactivity at each time point. Washed cells then stained with a FITC-conjugated pan-γδ TCR mAb (IMMU510) and propidium iodide (PI; Thermo Scientific) and analyzed by flow cytometry as described above to determine the percentage of dead cells (PI-positive) in the $\gamma\delta^+$ population.

Determination of DNA double-strand breaks

γδ-T cells radiolabeled as described in the manuscript and suspended in RPMI were seeded in duplicate onto poly-L-lysine-coated glass coverslips, at 2.5×10^5 cells per coverslip. After 1 h incubation at 37 °C, the plate was centrifuged, the supernatant was removed and the cells were fixed and permeabilized with 3.7 % formalin, 0.5 % Triton X-100 and 0.5 % IGEPAL® CA-630 in PBS. The cells were blocked with 2 % bovine serum albumin and 1 % goat serum in PBS. The cells were then incubated with an anti-phospho-histone H2A.X (Ser139) mouse mAb (1:1600; clone JBW301, Merck #05-636) overnight at 4 °C, then with an Alexa Fluor™ 488-conjugated goat anti-mouse IgG (H+L) secondary antibody (1:500; Jackson ImmunoResearch Laboratories #115-545-062) for 2 h at RT, and Hoechst 33342 for 1 min at RT. After mounting onto glass slides, images of cell nuclei and γH2AX foci were acquired on a TCS SP5 II confocal microscope (Leica) equipped with a 100×/1.40 HCX PL Apochromat oil immersion objective (Leica) and the Leica LAS-AF control software. Scanning was performed using 405 nm (blue diode) and 488 nm (argon) laser lines with a pinhole of 153 µm. Ten contiguous, non-overlapping optical sections of approximately 0.4 µm thickness through the cell nuclei were imaged. At least 30 nuclei per slide were imaged, with 2 slides per treatment. Illumination and acquisition parameters were kept constant within an experiment. Maximal intensity projections of *z*-stacks in

each channel were made using ImageJ v1.51p (http://imagej.nih.gov/ij). Nuclei and γH2AX foci were then counted using CellProfiler (http://cellprofiler.org) v2.2.0[1]. Nuclei (blue channel) were detected using a shape-based method (size filter: 30-80 pixels) to create mask images. Nuclei touching the edge of the images or too clumped to be distinguished by the software were excluded. A speckle-enhancing algorithm was applied to γH2AX images (green channel), followed by the mask image to remove out-of-nucleus signal. γH2AX foci were then detected on an intensity-based method with a size filter of 3-9 pixels, assigned to the corresponding nuclei and counted. The average number of γH2AX foci per nucleus was calculated for each image.

Immunohistochemistry

FFPE organs were processed by UCL IQPath (London, UK) for histologic analysis. FFPE organ blocks were sliced and stained with hematoxylin & eosin. Immunohistochemistry was performed with a Discovery XT system (Ventana Medical Systems) using the DAB Map detection kit (Ventana #760-124). For pre-treatment, CC1 (Ventana #950-124) was used. Sections were stained with anti-GFP (for tumor cell detection; rabbit polyclonal, 1/1000, Abcam #ab290, UK), anti-gamma/delta TCR (for γδ-T cell detection; clone 5A6.E9, ThermoFisher #TCR1061; clone B1.1, eBioscience #16-9959-81) or anti-CD3 (for human T cell detection; clone LN10, Leica #CD3-565-L-CE) primary antibodies, followed by biotinylated anti-rabbit or anti-mouse IgG (1/200; Dako) secondary antibodies, as appropriate. To the best of our knowledge, the only monoclonal antibodies that detect human γδ-TCR in paraffin-embedded samples are clones γ3.20, B1 and 5A6.E9[2–4], of which clone γ3.20 is no longer commercially available. We tested both B1 and 5A6.E9 and were unable to distinguish between control tumors devoid of γδ-T cells and tumors directly injected with γδ-T cells. Human CD3 was therefore used as a surrogate marker for γδ-T cells (Figure 5 and Figure S8).

Supplemental References

1. Carpenter, A.E., Jones, T.R., Lamprecht, M.R., Clarke, C., Kang, I., Friman, O., et al. (2006). CellProfiler: image analysis software for identifying and quantifying cell phenotypes. Genome Biol. *7*, R100.

2. Roullet, M., Gheith, S.M.F., Mauger, J., Junkins-Hopkins, J.M. and Choi, J.K. (2009). Percentage of γδ T cells in panniculitis by paraffin immunohistochemical analysis. Am. J. Clin. Pathol. *131*, 820–826.

3. Pollinger, B., Junt, T., Metzler, B., Walker, U.A., Tyndall, A., Allard, C., et al. (2011). Th17 cells, not IL-17+ T cells, drive arthritic bone destruction in mice and humans. J. Immunol. *186*, 2602–2612.

4. Tanaka, T., Yamamoto, H., Elsayed, A.A., Satou, A., Asano, N., Kohno, K., et al. (2016). Clinicopathologic spectrum of gastrointestinal T-cell lymphoma. Am. J. Surg. Pathol. *40*, 777–785.
