# Supplementary material for: In Vivo PET Tracking of 89Zr-Labeled Vγ9Vδ2 T Cells to Mouse Xenograft Breast Tumors Activated with Liposomal Alendronate
Source: Mol Ther. 2018 Oct 16;27(1):219–29. doi: 10.1016/j.ymthe.2018.10.006 (PMC6318719; doi:10.1016/j.ymthe.2018.10.006)
Supplement: Document S2. Article plus Supplemental Information [file mmc2.pdf]

Molecular Therapy
Original Article

In Vivo PET Tracking of ^{89}Zr-Labeled Vγ9Vδ2 T Cells to Mouse Xenograft Breast Tumors Activated with Liposomal Alendronate

Francis Man,[1] Lindsay Lim,[1] Alessia Volpe,[1] Alberto Gabizon,[2] Hilary Shmeeda,[2] Benjamin Draper,[3] Ana C. Parente-Pereira,[3] John Maher,[3] Philip J. Blower,[1] Gilbert O. Fruhwirth,[1] and Rafael T.M. de Rosales[1]

[1]School of Biomedical Engineering & Imaging Sciences, King's College London, St Thomas' Hospital, London SE1 7EH, UK; [2]Oncology Institute, Shaare Zedek Medical Center and Hebrew University—School of Medicine, Jerusalem 9103102, Israel; [3]School of Cancer and Pharmaceutical Sciences, King's College London, Guy's Hospital, London SE1 9RT, UK

Gammadelta T (γδ-T) cells are strong candidates for adoptive immunotherapy in oncology due to their cytotoxicity, ease of expansion, and favorable safety profile. The development of γδ-T cell therapies would benefit from non-invasive cell-tracking methods and increased targeting to tumor sites. Here we report the use of [^{89}Zr]Zr(oxinate)$_4$ to track Vγ9Vδ2 T cells *in vivo* by positron emission tomography (PET). *In vitro*, we showed that ^{89}Zr-labeled Vγ9Vδ2 T cells retained their viability, proliferative capacity, and anti-cancer cytotoxicity with minimal DNA damage for amounts of ^{89}Zr ≤20 mBq/cell. Using a mouse xenograft model of human breast cancer, ^{89}Zr-labeled γδ-T cells were tracked by PET imaging over 1 week. To increase tumor antigen expression, the mice were pre-treated with PEGylated liposomal alendronate. Liposomal alendronate, but not placebo liposomes or non-liposomal alendronate, significantly increased the ^{89}Zr signal in the tumors, suggesting increased homing of γδ-T cells to the tumors. γδ-T cell trafficking to tumors occurred within 48 hr of administration. The presence of γδ-T cells in tumors, liver, and spleen was confirmed by histology. Our results demonstrate the suitability of [^{89}Zr]Zr(oxinate)$_4$ as a cell-labeling agent for therapeutic T cells and the potential benefits of liposomal bisphosphonate treatment before γδ-T cell administration.

INTRODUCTION

Adoptive transfer of therapeutic T cells is a growing field in immuno-oncology, with spectacular clinical results against melanoma and hematological cancers.[1–3] Gammadelta-T (γδ-T) cell therapy is one type of T cell therapy being explored, with recent data showing intra-tumoral γδ-T cells are the single most favorable prognostic immune cell infiltrate.[4] γδ-T cells perform roles belonging to both adaptive and innate immunity, playing a significant role in anti-infectious and anti-tumor immune surveillance.[5] Activated γδ-T cells are highly cytotoxic, enhance the function of other immune cells, and act as antigen-presenting cells.[6] In humans, the Vγ9Vδ2 subtype of γδ-T cells represents 1%–5% of circulating CD3$^+$ T cells.[6] Their potent cytotoxicity and high proliferative capacity have made them candidates of choice for cancer immunotherapy.[7]

The unique activation of Vγ9Vδ2 cells by phosphoantigens such as isopentenyl pyrophosphate (IPP)[8] allows them to discriminate between normal and metabolically disordered cells based on IPP expression levels.[9] The activation and targeting of γδ-T cells to tumor tissue could, therefore, be improved by selectively increasing the presentation of phosphoantigens in cancer cells, for example, by using liposome- or nanocarrier-based formulations of aminobisphosphonate drugs (NBPs).[10] NBPs (e.g., pamidronate, alendronate, and zoledronate),[11] which increase the expression of IPP in target cells by inhibiting farnesyl diphosphate synthase, are hydrophilic molecules that accumulate in bone, but not in other tissues, and are rapidly cleared from the circulation. Encapsulating alendronate in liposomes has been shown to increase the therapeutic efficacy of γδ-T cells in preclinical models.[12,13]

Clinical studies of γδ-T cell immunotherapy have shown a good safety profile and efficacy comparable to second-line anticancer therapies, but they have also highlighted the need for improvements.[14,15] Unknown aspects of adoptive γδ-T cell therapy include their *in vivo* distribution and kinetics of arrival at the tumor site. Whole-body imaging is highly useful in this context by enabling *in vivo* tracking of administered cells. Many techniques exist for non-invasive cell tracking;[16–18] however, only nuclear imaging, and particularly positron emission tomography (PET), provides sensitive and quantitative, whole-body information with adequate spatiotemporal resolution. Hence, methods to radiolabel and track therapeutic cells using positron-emitting radionuclides are likely to become important tools for cell immunotherapy.[19]

Received 5 June 2018; accepted 8 October 2018;
https://doi.org/10.1016/j.ymthe.2018.10.006.

Correspondence: Rafael T.M. de Rosales, PhD, School of Biomedical Engineering & Imaging Sciences, King's College London, St Thomas' Hospital, Westminster Bridge Road, London SE1 7EH, UK.
E-mail: rafael.torres@kcl.ac.uk

This is an open access article under the CC BY license (http://creativecommons.org/licenses/by/4.0/).

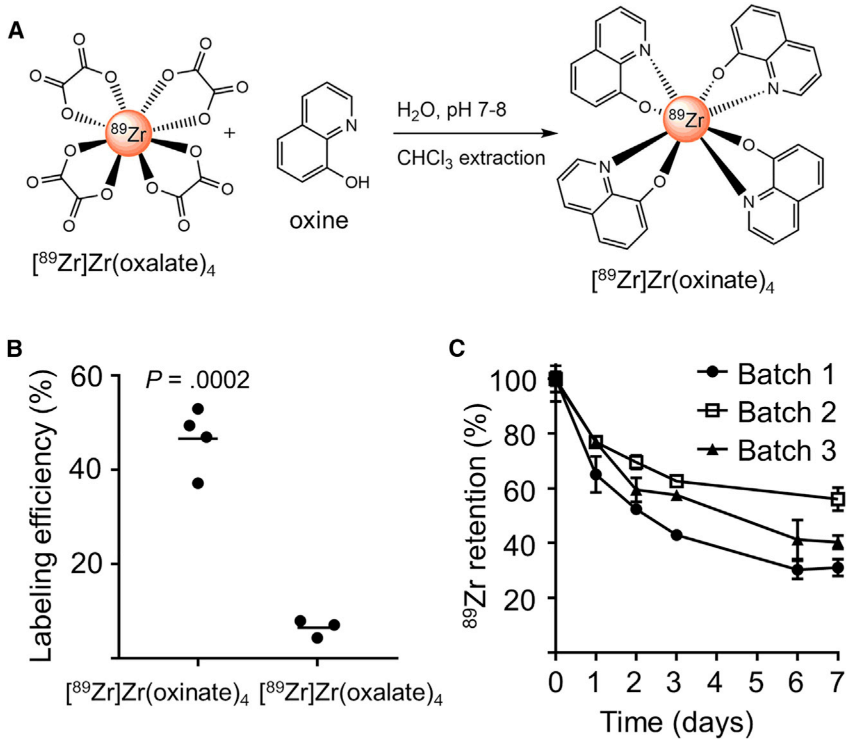

Figure 1. Radiotracer Synthesis and γδ-T Cell Radiolabeling

(A) [^{89}Zr]Zr(oxinate)$_4$ synthesis. (B) Labeling efficiencies of γδ-T cells incubated with ^{89}Zr-based tracers (63.2 ± 7.9 mBq/cell) 20 min at RT. Mean of N = 3–4 individual experiments (unpaired t test). (C) ^{89}Zr retention by γδ-T cells over 7 days after labeling with [^{89}Zr]Zr(oxinate)$_4$ (average incorporated activity: 34.3 ± 6.0 mBq/cell). Mean ± SEM of triplicate measures for 3 cell batches.

PET tracking of T cells has been performed with radiolabeled antibodies, antibody fragments, or lipophilic small molecules[20,21] and by reporter-gene imaging.[22] When genetic engineering is not required, e.g., for γδ-T cells, a clinically applicable alternative to reporter-gene imaging is direct cell labeling with PET radionuclides. Immune cells have long been imaged clinically by single-photon emission computed tomography (SPECT) in this manner, for example, using [^{111}In]In(oxinate)$_3$ and [^{99m}Tc]Tc-exametazime.[19] In this regard, the clinically approved 8-hydroxyquinoline (oxine) has been recently shown to be an excellent ionophore for cell labeling with ^{89}Zr ($t_{1/2}$ = 78.4 hr, β^+ = 22.3%).[23–25] However, to the best of our knowledge, no study has evaluated its use for tracking γδ-T cells.

Here we report the first use of [^{89}Zr]Zr(oxinate)$_4$ for *in vitro* radiolabeling and *in vivo* tracking of human γδ-T cells, including the effects of radiolabeling on γδ-T cell functionality, proliferation, and DNA integrity. We applied this strategy in a xenograft model of breast cancer with an engineered cancer cell line that allows multimodal imaging to track tumor cells. A liposomal aminobisphosphonate was administered to increase T cell trafficking to the tumor.

RESULTS

Radiotracer Labeling Efficiency and Retention in γδ-T Cells

[^{89}Zr]Zr(oxinate)$_4$ was obtained by mixing neutralized [^{89}Zr]Zr(oxalate)$_4$ with 8-hydroxyquinoline dissolved in chloroform (Figure 1A). The radiochemical yield was 77.6% ± 11.8% (mean ± SD, N = 21), and radiochemical purity established by thin-layer radiochromatography was >95% (Figure S1). γδ-T cell labeling efficiency with [^{89}Zr]Zr(oxinate)$_4$ (46.6% ± 3.4%, N = 4) was significantly higher than with [^{89}Zr]Zr(oxalate)$_4$ (6.5% ± 1.1%, N = 3; Figure 1B). To optimize radiolabeling conditions, cells were incubated with [^{89}Zr]Zr(oxinate)$_4$ (6–600 mBq/cell) for 10, 20, or 30 min at 4°C, room temperature (RT), or 37°C. We found no significant difference between incubation times and temperatures (Figure S2).

To study long-term tracer retention, radiolabeled γδ-T cells (25–40 mBq/cell) were cultured at 0.83 × 10^6 cells/mL. After 24 hr, the percentage of cell-associated ^{89}Zr was 72.9% ± 6.8% of the original activity, and 42.4% ± 12.6% after 1 week (N = 3; Figure 1C).

In Vitro Assays of ^{89}Zr-Radiolabeled γδ-T Cells

The purity of *in vitro*-expanded γδ-T cells plateaued 13–15 days post-isolation (Figure S3), at which point they were radiolabeled. Cells labeled with 6–20 mBq/cell proliferated similarly to unlabeled cells ($p \geq 0.05$; Figure 2A), while cells labeled with more than 50 mBq/cell ceased to proliferate *in vitro*, indicating a dose-dependent effect of ^{89}Zr on γδ-T cell proliferation. A similar dose dependency was observed on γδ-T cell death (Figure 2B) and DNA damage, evaluated by the formation of γH2AX foci[26] 1 hr after labeling (Figures 2C and 2D).

To evaluate the cytotoxic ability of radiolabeled γδ-T cells, we quantified the survival of MDA-MB-231.hNIS-GFP cancer cell monolayers. γδ-T cells labeled with up to 600 mBq/cell showed no significant difference in cancer cell killing compared to unlabeled γδ-T cells (Figure 2E). As a control, adding ^{89}Zr up to 3 Bq/cancer cell in the medium was not toxic to cancer cells in the absence of γδ-T cells. Even in 30-fold excess, γδ-T cells showed no toxicity toward cancer cells in the absence of aminobisphosphonate (Figure S4).

In Vivo PET Tracking of ^{89}Zr-Radiolabeled γδ-T Cells

^{89}Zr-radiolabeled γδ-T cells were administered intravenously in a mouse xenograft model of breast cancer followed by PET imaging

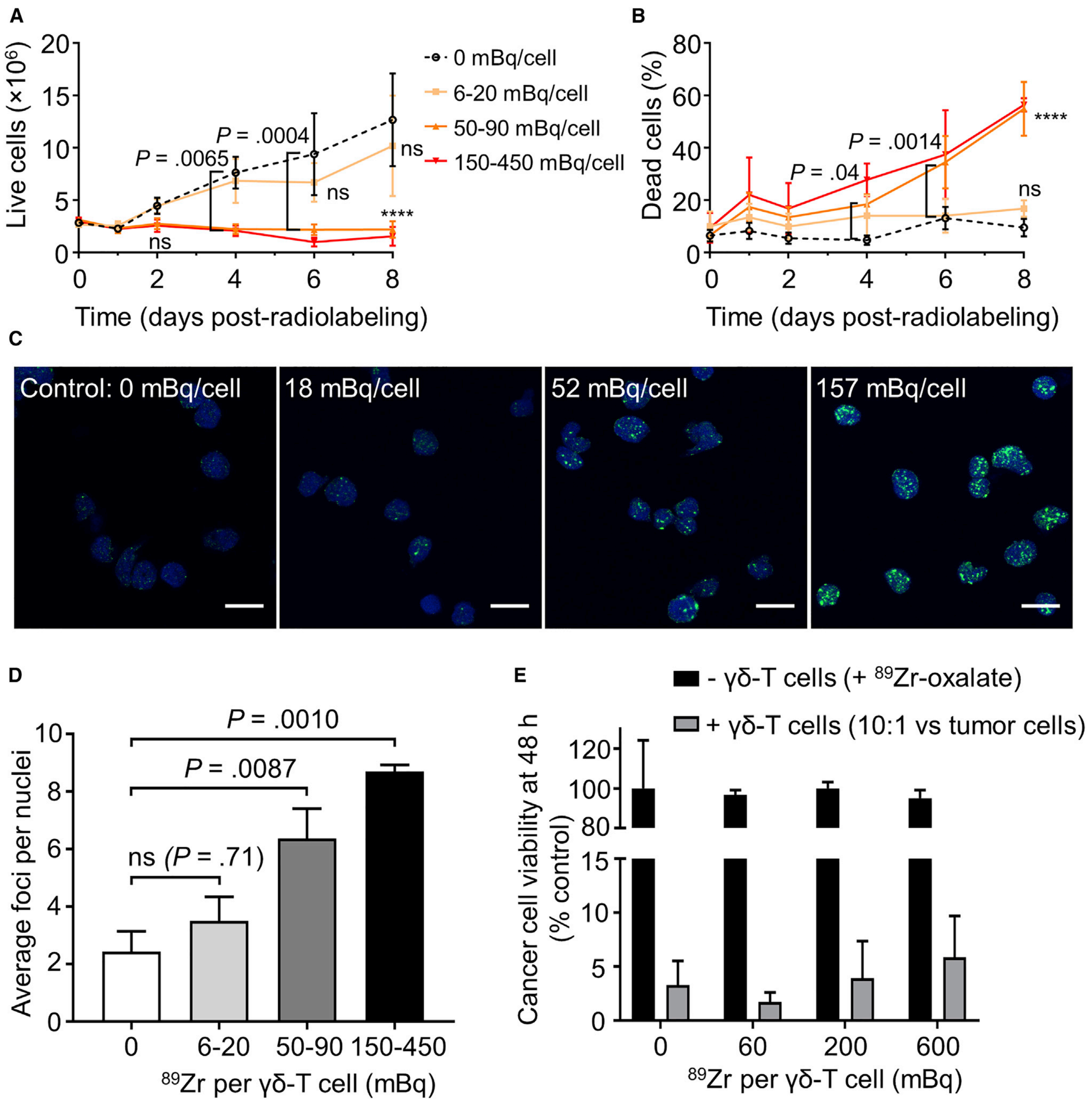

Figure 2. Assays of ^{89}Zr-Radiolabeled γδ-T Cells

(A and B) *In vitro* growth (A) and mortality (B) of radiolabeled γδ-T cells. Mean ± SEM of N = 4 independent experiments (except 150–450 mBq group, N = 2, not included in statistical analysis). ns: $p > 0.05$; ****$p < 0.0001$ versus unlabeled cells (2-way repeated-measures ANOVA, Dunnett's correction for multiple comparisons). (C) Representative images of γ-H2AX foci (green) and nuclei (blue) in radiolabeled γδ-T cells (scale bars, 10 μm). (D) Average number of γ-H2AX foci per nuclei after radiolabeling. Mean ± SEM of N = 6, 5, 6, and 3 independent experiments (1-way ANOVA, Dunnett's correction). (E) MDA-MB-231.hNIS-GFP tumor cell viability 48 hr after adding γδ-T cells or unchelated ^{89}Zr, expressed as a percentage of control (tumor cells without γδ-T cells and ^{89}Zr). Mean ± SEM of N = 3 independent experiments (2-way repeated-measures ANOVA, Dunnett's correction).

at 1 hr, 48 hr, and 7 days after injection. We imaged the hNIS-expressing cancer cells by SPECT using $^{99m}TcO_4^-$.[27] We also evaluated the effect of PLA on γδ-T cell homing to tumor sites. The study schedule is provided in Figure 3A.

The PLA dosing schedule was established using ^{111}In-labeled PLA, showing significant PLA tumor accumulation within 24–72 hr of administration (Figure 3B; Table S1). The experimental group (PLA treated) received radiolabeled γδ-T cells + PLA (5 mg/kg alendronate).

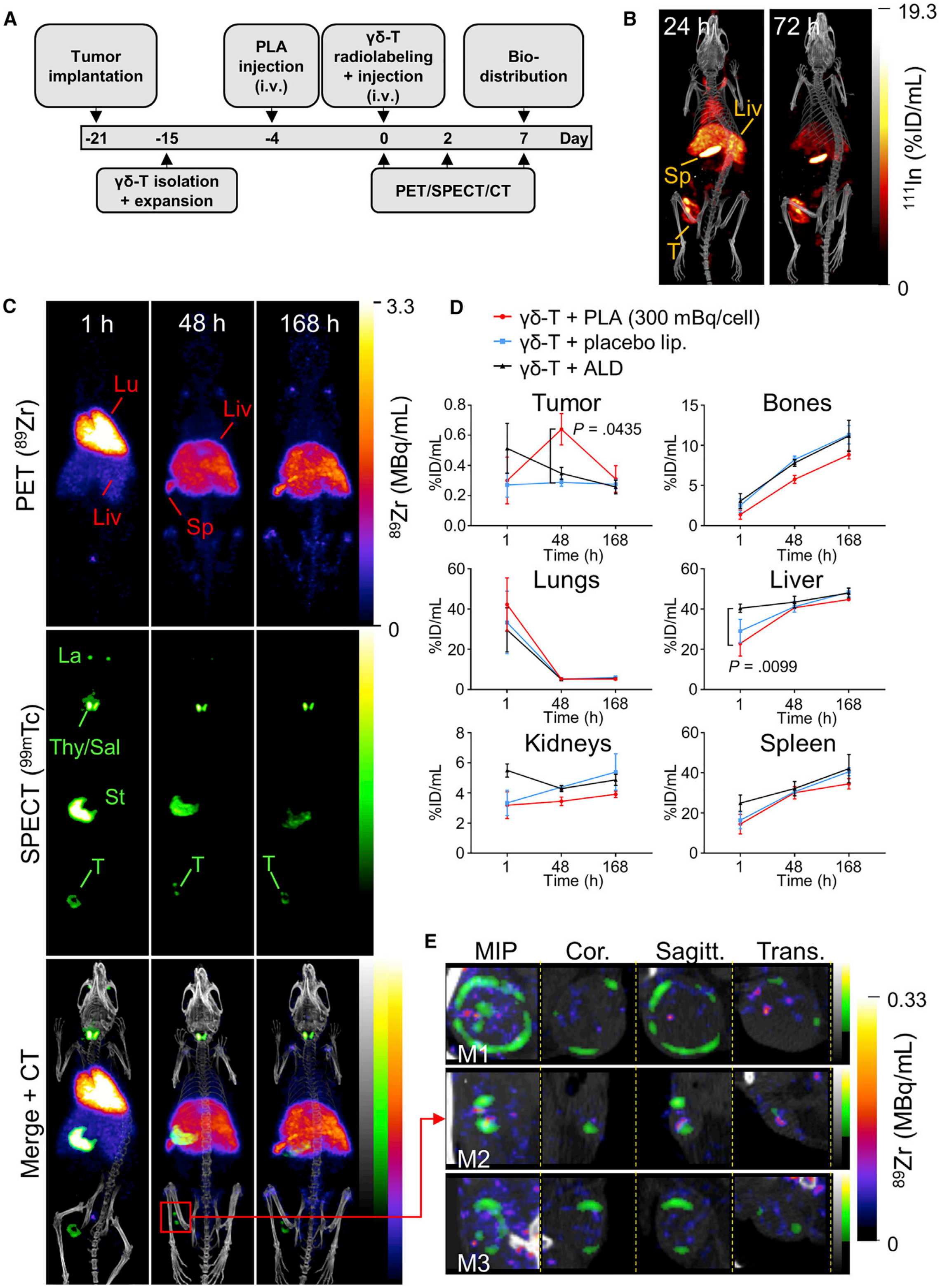

(legend on next page)

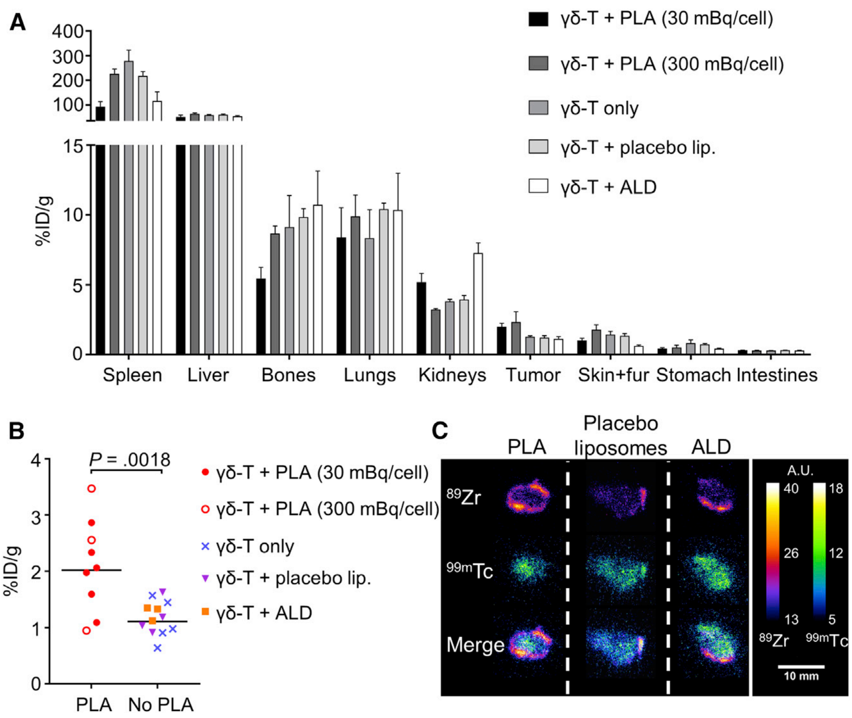

Figure 4. PLA Treatment Increases the Accumulation of γδ-T Cells in Tumors

(A) *Ex vivo* bio-distribution of radiolabeled γδ-T cells, 7 days after γδ-T cell administration. Mean ± SEM of ^{89}Zr uptake after PLA (N = 6 and 3, respectively), placebo liposomes (N = 4), non-liposomal alendronate (ALD; N = 3), or vehicle (N = 5) treatment. Data are from 3 pooled independent experiments (total N = 21). (B) Comparison of ^{89}Zr accumulation in the tumor between PLA (N = 9) and non-PLA (N = 12) treatments (unpaired t test). (C) Artificially colored autoradiographs of tumor sections after PLA, placebo liposomes or non-liposomal alendronate (ALD) treatment. Images are representative of N = 3, 4, and 3 animals per group (scale bar, 10 mm).

Control groups (non-PLA treated) received radiolabeled γδ-T cells with placebo liposomes, non-liposomal alendronate, or saline. An additional control group received γδ-T cells killed by freeze-thawing to compare bio-distributions of viable and non-viable cells.

SPECT showed uptake of $^{99m}TcO_4^-$ in tumors and endogenous NIS-expressing organs (thyroid, salivary, and lacrimal glands and stomach; Figure 3C). At 1 hr after intravenous administration of ^{89}Zr-radiolabeled γδ-T cells, PET revealed high amounts of radioactivity in the lungs in all groups, with signal also observed in the liver and spleen (Figures 3C and 3D). There was significantly higher uptake in the liver in the ALD group versus the PLA group. At tumor sites, the ^{89}Zr signal was close to background (Figure S5). After 48 hr, ^{89}Zr activity increased in the liver, spleen, and bones in all groups and decreased in the lungs. Uptake of ^{89}Zr was observed at the tumor site only in the PLA group (Figure S5), suggesting the presence of radiolabeled γδ-T cells. Importantly, this was significantly higher in PLA-treated animals compared to control animals treated with non-liposomal alendronate (Figure 3D). Enlarged tumor views showed heterogeneity in tumor tissue, with live tissue, expressing a functional hNIS protein[18,27] and represented by a donut of ^{99m}Tc signal surrounding a core of non-viable tumor cells (Figure 3E). ^{89}Zr signal in tumors was heterogeneous, with some co-localizing with ^{99m}Tc at the edges and foci of ^{89}Zr signal inside the tumor. After 7 days, ^{89}Zr activity remained high in the liver; increased in the spleen, bones, and kidneys; and was indistinguishable from background in tumors. Uptake values are provided in Table S2. Compared to other treatment groups, PET images of killed γδ-T cells showed a higher accumulation in the liver immediately after injection and increased uptake of ^{89}Zr in the kidneys at later time points (Figure S6).

Ex Vivo Bio-distribution of ^{89}Zr-Radiolabeled γδ-T Cells

Ex vivo γ-counting 7 days post-administration of radiolabeled cells revealed a high concentration of ^{89}Zr in the spleen (153.5% ± 88.8% injected dose [ID]/g averaged across all groups, N = 24) and liver (58.1% ± 10.6% ID/g, N = 24) in all groups, followed by lung and bone tissue (Figure 4A). Uptake of ^{89}Zr in tumors from PLA-treated groups (2.1% ± 0.8% ID/g) was significantly higher than in non-PLA groups (1.2% ± 0.3% ID/g; Figure 4B), suggesting higher γδ-T cell numbers in PLA-treated tumors. Bone uptake of ^{89}Zr in PLA-treated groups (6.5% ± 0.8% ID/g, N = 9) was significantly lower than in other groups (10.0% ± 1.1% ID/g, N = 12; p = 0.0238). Uptake in kidneys was significantly higher with killed γδ-T cells than in other treatment groups (Table S3). Uptake in other organs showed no major differences between treatment groups.

Tumor section autoradiographs showed a strong signal originating from hNIS-accumulated $^{99m}TcO_4^-$. Autoradiography was repeated after 4 days to allow for the decay of ^{99m}Tc and the capture ^{89}Zr signal.

Figure 3. *In Vivo* Tracking of Radiolabeled γδ-T Cells

(A) Experiment schedule. (B) Representative SPECT-CT images of MDA-MB-231.hNIS-GFP xenograft NSG mice 24 and 72 hr after ^{111}In-labeled PLA administration. (C) Representative PET, SPECT, and CT (merged) scans of a PLA-treated SCID/beige mouse at 1, 48, and 168 hr post-injection of γδ-T cells. Liv, liver; Lu, lungs; Sp, spleen; T, tumor. Endogenous murine NIS expression also results in radiotracer uptake, giving rise to the following signals: La, lacrimal glands; St, stomach; and Thy/Sal, thyroid/salivary glands. (D) Time-activity curves from image-based quantification of ^{89}Zr in selected organs. Mean ± SEM of N = 3–4 animals (repeated-measures MM analysis, Bonferroni correction for multiple comparisons). (E) Enlarged maximum intensity projection (MIP), coronal, sagittal, and transversal tumor views (merged PET- and SPECT-CT) in three PLA-treated mice (M1, M2, and M3), 48 hr after γδ-T cell injection.

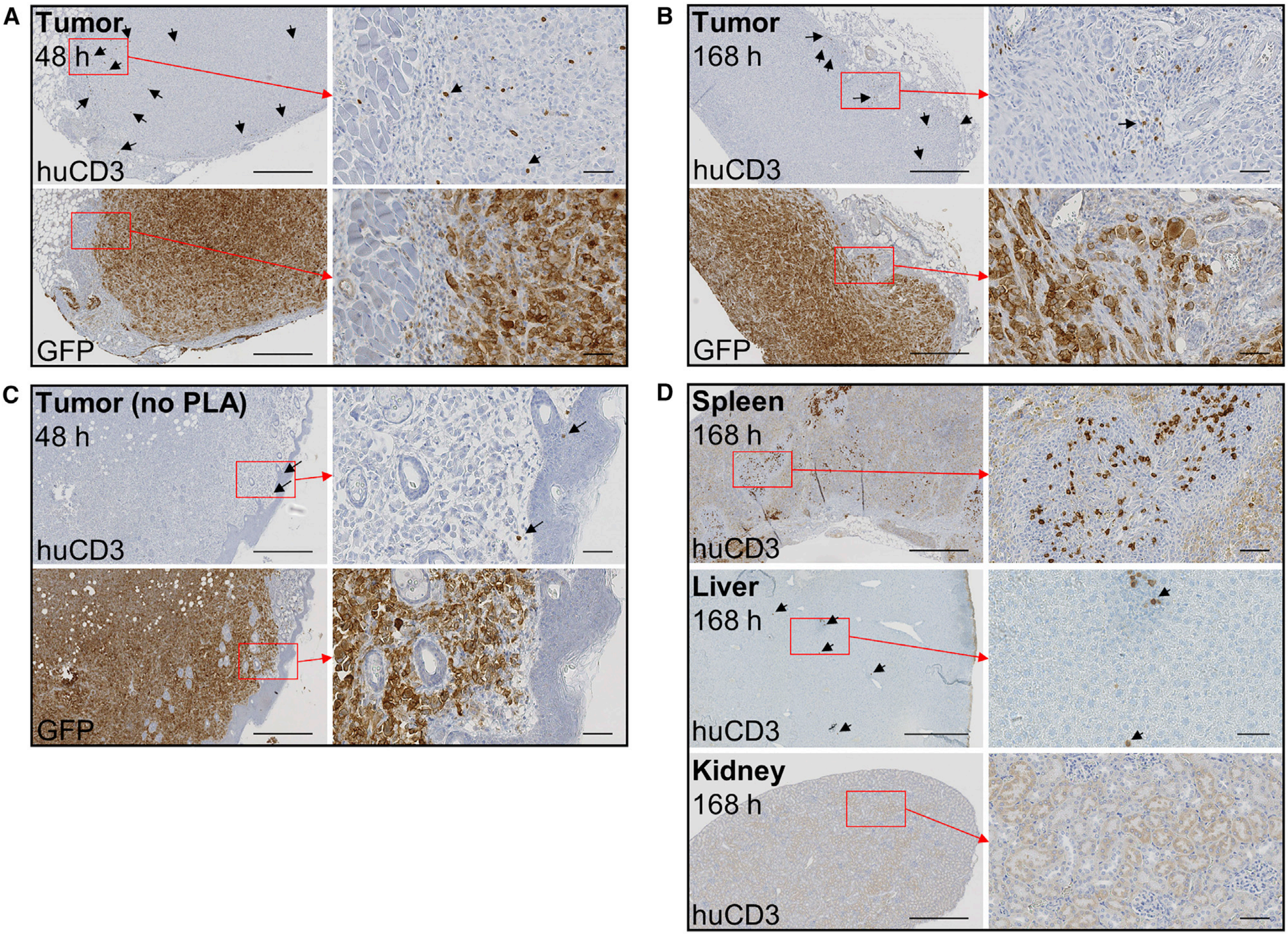

Figure 5. Histology of γδ-T Cells

(A–C) Tumor sections 48 hr (A and C) or 7 days (B) after the injection of ^{89}Zr-radiolabeled γδ-T cells into mice treated with PLA (A and B) or without PLA (C), stained for human CD3 (γδ-T cells) or GFP (tumor cells). Arrows indicate representative CD3^{+} cells. (D) Spleen, liver, and kidney sections 7 days after the administration of ^{89}Zr-radiolabeled γδ-T cells. Sections are representative of N = 2–3 animals per time point. 6× (left) and 30× (right) magnification; scale bars, 500 μm (left) and 50 μm (right).

Sections from PLA-treated animals showed increased ^{89}Zr signal compared to non-PLA-treated animals. The ^{89}Zr signal was higher in the tumor periphery, whereas the ^{99m}Tc signal was uniformly distributed (Figure 4C). γδ-T cell presence in tumors was demonstrated by immunohistochemistry. Human CD3-positive cells (>95% γδ-T cell receptor [TCR]$^{+}$ at the time of administration; Figure S3) were visible in tumors 48 hr and 7 days after injection, both in the periphery and deeper regions (Figures 5A–5C; Figure S7). These cells were also visible in the spleen and liver after 7 days, but not in kidney sections (Figure 5D) or in control tissues of mice not administered γδ-T cells (Figure S8).

DISCUSSION

[^{89}Zr]Zr(oxinate)$_4$ synthesis has been reported previously by our group[23] and others.[24,25] The temperature-independent labeling efficiency of γδ-T cells with [^{89}Zr]Zr(oxinate)$_4$ suggests this is a passive process, in line with results from Sato et al.[24] Sufficient radiotracer retention within cells is important to ensure that the imaging signal reflects labeled cells rather than free radiotracer bio-distribution. We observed an efflux of approximately half of the incorporated ^{89}Zr over 1 week *in vitro*, which we believe does not interfere with *in vivo* imaging within this time frame. Uptake of ^{89}Zr in the bone can be used to estimate the amount of tracer that leaked from the cells.[25,28] Retention of [^{89}Zr]Zr(oxinate)$_4$ is dependent on cell type, and our results are comparable to those observed with dendritic, bone marrow, and chimeric antigen receptor (CAR)-T cells.[23–25] Comparable levels of tracer efflux have been observed from lymphocytes labeled with [^{111}In]In(oxinate)$_3$,[29,30] the current gold standard for cell tracking by nuclear imaging.

A radiotracer for cell tracking must not significantly alter the phenotype, survival, proliferation capacity, and functionality of labeled cells. We demonstrated that the effects of [^{89}Zr]Zr(oxinate)$_4$ on γδ-T cell survival, proliferation capacity, and DNA damage were kept minimal for doses up to 20 mBq/cell but were significant at doses ≥50 mBq/cell. The cytotoxicity of radiolabeled γδ-T cells

against the same tumor cells used for *in vivo* experiments was not affected by amounts of $[^{89}Zr]Zr(oxinate)_4$ of up to 600 mBq/cell, at least within 48 hr of radiolabeling. Cancer cell death was due to the combination of bisphosphonate treatment and γδ-T cells and not to the presence of ^{89}Zr. Preserved cytotoxicity after radiolabeling, also recently observed in CAR-T cells by Weist et al.,[25] is encouraging for the use of $[^{89}Zr]Zr(oxinate)_4$ as a T cell-tracking agent. However, the therapeutic efficacy of γδ-T cells presumably also relies on their *in vivo* proliferation ability; hence, we suggest that radiolabeling γδ-T cells with $[^{89}Zr]Zr(oxinate)_4$ should ideally not exceed 20 mBq/cell. This could lead to sensitivity issues on conventional PET scanners. Indeed, our experiments show that *ex vivo* gamma-counting tumors could reveal amounts of ^{89}Zr indistinguishable from background in our PET imaging system at day 7. Assuming that a human cell-tracking study would require 37 MBq ^{89}Zr[31] and 10^9 γδ-T cells,[14] this would equate to an average of 37 mBq/cell, which we have shown not to be excessively damaging to γδ-T cells. Upcoming developments in PET technology, such as total-body PET,[32] should reduce the required ^{89}Zr activity per cell (by a factor of 40) and overcome these sensitivity issues.

For *in vivo* studies, a xenograft model of human breast cancer in immunocompromised mice[12,33] was chosen, as mice do not possess a subset of T cells functionally equivalent to human Vγ9Vδ2 T cells.[34] We tracked γδ-T cells radiolabeled with $[^{89}Zr]Zr(oxinate)_4$ (30–300 mBq/cell) by PET 1 hr, 48 hr, and 7 days after intravenous injection. We simultaneously used $^{99m}TcO_4^-$ to visualize hNIS-expressing tumors by SPECT. The *in vivo* distribution of ^{89}Zr-labeled γδ-T cells over time was similar to that observed in studies of adoptively transferred γδ-T[35,36] and other T cells.[25,37,38] ^{89}Zr uptake was significantly increased in PLA-treated tumors, suggesting that PLA increases homing of these cells to the tumor site. Accumulation of γδ-T cells at the tumor site 48 hr after administration was also observed by others.[35] Uptake values for the spleen and tumor determined by image-based quantification are lower than those determined by *ex vivo* bio-distribution. This can be explained by the small size of this organ and significant partial volume effect (spleen) and the liquid or necrotic tumor core that leaked upon dissection.

For instrument sensitivity reasons, some imaging studies were performed with higher doses of ^{89}Zr than recommended above. However, the distinctly different distribution pattern observed with killed γδ-T cells suggests that radiolabeling with up to 300 mBq/cell, which preserved cytotoxic functionality *in vitro* over 48 hr, did not impair γδ-T cell trafficking and allowed us to track live cells. Furthermore, previous studies have shown that $[^{89}Zr]Zr(oxalate)_4$,[28,39] $[^{89}Zr]Zr(oxinate)_4$, and lysates from $[^{89}Zr]Zr(oxinate)_4$-labeled cells[23] have distinct distribution patterns from intact cells labeled with $[^{89}Zr]Zr(oxinate)_4$. Cell concentrations during labeling and *in vitro* assays were in the range of $1-5 \times 10^6$/mL. In comparison, using *in vitro* ^{89}Zr retention values, cell concentrations extrapolated from PET-computed tomography (CT) images in the organs showing the strongest ^{89}Zr signal (spleen, liver, and lungs) would be in the range of $0.5-5 \times 10^6$ cells/mL. We therefore expect the DNA damage sustained by γδ-T cells, due to both self-irradiation and crossfire, after *in vivo* administration to be comparable to that observed *in vitro*. Considering the strong affinity of the $^{89}Zr^{4+}$ ion for bone,[28] the relatively low bone accumulation of ^{89}Zr indicates limited efflux of weakly chelated ^{89}Zr, and it suggests that ^{89}Zr is mostly retained by γδ-T cells after injection. The lower accumulation of ^{89}Zr in the bones of PLA-treated animals compared to other groups also suggests reduced efflux of ^{89}Zr from γδ-T cells after PLA treatment.

Histology confirmed the presence of γδ-T cells in the tumors, spleen, and liver, using the CD3 marker.[40] Immunohistochemistry and autoradiography suggest that γδ-T cells accumulated mostly at the periphery of the tumor. The small number of cells observed by immunohistochemistry precludes statistical comparison. Furthermore, these techniques can only image the solid portion of the tumor. PET imaging not only allowed visualization of the whole, intact tumors but additionally revealed heterogeneous distributions of ^{89}Zr in tumors, which would be challenging to observe by histology. Combined with the non-invasive nature of PET imaging, this further highlights the value of using PET tracers such as $[^{89}Zr]Zr(oxinate)_4$ for cell tracking. The high uptake of ^{89}Zr in the liver and spleen was mirrored by the large numbers of human $CD3^+$ cells observed in these tissues, consistent with the bio-distribution of radiolabeled γδ-T cells. In contrast, the apparent absence of $CD3^+$ cells in the kidneys, despite higher ^{89}Zr uptake than in the tumor, and the fact that the kidney uptake of ^{89}Zr was significantly higher in animals administered killed γδ-T cells than in other groups both suggest that the radioactivity detected in the kidneys corresponds to ^{89}Zr progressively released from γδ-T cells in other organs. A limitation of directly labeling cells is that the radionuclide can leak out over time and be taken up by adjacent tissue. Although immunohistochemistry demonstrates the presence of the administered γδ-T cells in the tumors, this technique cannot determine whether the ^{89}Zr signal originates from the γδ-T cells or from *in situ*-labeled bystander cells.

A critical aspect of this type of cellular immunotherapy is that the therapeutic cells must be activated at the target site and reach the tumor in sufficient numbers. γδ-T cell toxicity toward cancer cells is greatly amplified by bisphosphonates, suggesting a role for γδ-T cells in the anti-cancer properties of bisphosphonates.[41] Here we sought to increase phosphoantigen expression in tumors by administering PLA, which delivers alendronate to the tumors in an untargeted fashion by virtue of the enhanced permeability and retention (EPR) effect.[42] Liposomal alendronate proved safer than other bisphosphonates and effective in potentiating γδ-T cell therapy.[12,43] We have previously shown that the tumor-to-background uptake ratio of PLA increases over time and is significant after 3 days.[44] Here we observed that PLA administered 4 days in advance significantly increased the amount of ^{89}Zr reaching the tumor within 48 hr of radiolabeled γδ-T cell administration. Our results suggest that γδ-T cells home to the tumor within 2 days and remain there for at least 5 days. This was not observed in any other treatment group, demonstrating the importance of encapsulating the aminobisphosphonate in a tumor-targeting vehicle.

Clinical imaging studies of therapeutic T cells with [^{111}In]In (oxinate)$_3$ have been performed by radiolabeling only a fraction of the total administered T cells,[45–47] although evidence exists that distributing the total activity over a larger number of cells better preserves their proliferative abilities.[48] Our results suggest that radiolabeling the entire batch of γδ-T cells with [^{89}Zr]Zr(oxinate)$_4$ might be the preferable option to avoid imaging excessively damaged cells. In two notable studies, γ-scintigraphy revealed T cell uptake in tumors using only 1−3 mBq ^{111}In per cell.[46,49] Considering the increased sensitivity of PET over SPECT and expected improvements in PET technology, clinical imaging of T cell therapies using [^{89}Zr]Zr(oxinate)$_4$ is a credible prospect.

Conclusions

This study demonstrates the suitability of [^{89}Zr]Zr(oxinate)$_4$ as a PET tracer to track γδ-T cells *in vivo*, while previous work has shown the therapeutic efficacy of γδ-T cells in combination with PLA.[12,43] These objectives achieved, [^{89}Zr]Zr(oxinate)$_4$ can now be applied to answer fundamental questions in the preclinical and clinical development of γδ-T cell therapies, e.g., whether the accumulation of γδ-T cells at the tumor site or their distribution within the tumor correlates with therapeutic efficacy. Due to numerous molecular and cellular differences, the distribution of human γδ-T cells in an immunocompromised mouse model cannot fully predict their behavior in a human host. However, the results of this proof-of-principle study can be used to design a clinical trial that will answer the question of the distribution of γδ-T cells in humans after adoptive transfer.

Our results have implications for clinical translation, and they suggest using liposomal aminobisphosphonates as adjuncts to γδ-T cell therapy. In the context of clinical protocols involving repeated infusions of γδ-T cells,[15] one can envisage the use of ^{89}Zr-labeled cells for the first infusion, followed by PET imaging 24–72 hr later. The number of cells trafficking to the tumor sites would then be used to decide whether to pursue with additional treatment cycles. Cell radiolabeling with [^{89}Zr]Zr(oxinate)$_4$ is clinically translatable without significant methodological modifications, and the high similarity of [^{89}Zr]Zr(oxinate)$_4$ to the well-established [^{111}In]In(oxinate)$_3$ should facilitate regulatory approval. Our results support that T cell labeling with [^{89}Zr]Zr(oxinate)$_4$ is a realistic option for human studies and will benefit the development of cellular immunotherapy.

MATERIALS AND METHODS

Experiment Approval

Animals experiments were approved by the UK Home Office under The Animals (Scientific Procedures) Act (1986), PPL reference 7008879 (Protocol 6), with local approval from King's College London Research Ethics Committee (KCL-REC). Experiments using human T cells received approval from KCL-REC (Study Reference HR-16/17-3746). All donors provided written, informed consent.

Reagents, Animals, and Cells

Unless otherwise indicated, reagents were purchased from Sigma-Aldrich and Merck. Female SCID/beige (CB17.Cg-Prkdc^{scid}Lyst^{bg-J}/Crl) and Nod scid gamma (NSG) (NOD.Cg-Prkdc^{scid} Il2rg^{tm1Wjl}/SzJ) mice (18−25 g, 10−20 weeks old) were obtained from Charles River (UK). γδ-T cells were obtained as described previously,[12] using zoledronate (Novartis) and interleukin-2 (IL-2) (Novartis). Full details are provided in the Supplemental Materials and Methods. Population purity was assessed by flow cytometry (BD FACSCalibur), using pan-γδ TCR (IMMU510, Beckman Coulter B49175) and anti-CD3 (OKT3, BioLegend 317307) monoclonal antibodies. Data were analyzed using Flowing version (v.)2.5.1 (http://flowingsoftware.btk.fi). Only batches with ≥80% γδ-positive CD3$^+$ cells were used for further experiments (≥95% for *in vivo* experiments). MDA-MB-231.hNIS-GFP cells[27] were grown in DMEM supplemented with 10% fetal bovine serum (FBS), penicillin, streptomycin, and L-glutamine (2 mM), and they were tested for mycoplasma contamination (e-Myco PCR detection kit, Bulldog Bio).

PET Tracer Synthesis

[^{89}Zr]Zr(oxinate)$_4$ was synthesized as previously described.[23] Full details are provided in the Supplemental Materials and Methods.

Cell Labeling

γδ-T cells expanded *in vitro*[12] were washed with PBS (Ca^{2+}/Mg^{2+} free) and re-suspended at 5 × 10^6/mL in PBS at RT. [^{89}Zr]Zr (oxinate)$_4$ (6−600 mBq/cell) in aqueous DMSO was added to the cell suspension, keeping DMSO concentrations ≤0.7%. Neutralized [^{89}Zr]Zr(oxalate)$_4$ with an equivalent amount of DMSO was used as a control. After 10−30 min of incubation, cells were pelleted and the supernatants kept aside. The cells were washed with PBS, centrifuged, and the washings combined with the previous supernatants. The cells were suspended in growth medium or PBS for further experiments. Viability was assessed using the trypan blue dye exclusion method. Radioactivity in re-suspended cells and combined supernatants was measured in a gamma-counter. Cell-labeling efficiency (LE[%]) was calculated as follows.

$$LE(\%)=\frac{\text{activity of cell fraction}}{\text{activity of cell fraction + activity of combined supernatants}}$$

For radiotracer retention and cell proliferation studies, radiolabeled (or vehicle-treated) γδ-T cells were cultured as described above, and they were analyzed at various time points for viability (using trypan blue), determination of cell-associated radioactivity (by γ-counting), and cell death (by flow cytometry using propidium iodide [PI]; Thermo Scientific). Further details are provided in the Supplemental Materials and Methods.

Cancer Cell-Killing Assay

MDA-MB-231.hNIS-GFP cells seeded in a 96-well plate at 10^4 cells/well and incubated overnight were treated with 3 μM zoledronate or vehicle for 24 hr. The cells were washed and the medium was replaced with γδ-T cells in growth medium. As a control for radiolabeled γδ-T cells, an equal amount of ^{89}Zr in medium was added to some wells. After 48 hr, γδ-T cells were removed by washing with PBS, and cancer cell viability was evaluated using the alamarBlue assay (Thermo

Scientific), reading plates in a GloMax (Promega) reader (530 nm excitation and 590 nm emission filters).

Determination of DNA Double-Strand Breaks

Radiolabeled γδ-T cells in medium were seeded onto poly-L-lysine-coated coverslips and incubated for 1 hr. After centrifugation and gentle rinsing with PBS, the cells were fixed and permeabilized with 3.7% formalin, 0.5% Triton X-100, and 0.5% IGEPAL CA-630 in PBS, then blocked with 2% BSA and 1% goat serum. γH2AX foci were detected with an anti-γH2AX (Ser139) mouse monoclonal antibody (mAb) (1:1,600; JBW301, Merck 05-636) and goat anti-mouse AF488-immunoglobulin G (IgG) (1:500; Jackson ImmunoResearch Laboratories 115-545-062). Nuclei were detected with Hoechst 33342. Images were acquired on a TCS SP5 II confocal microscope (Leica) with a 100×/1.40 HCX PL Apochromat objective (Leica) and Leica Application Suite Advanced Fluorescence (LAS-AF) control software. Ten sections (0.4-μm thickness) were imaged. At least 30 nuclei/slide were imaged (2 slides/treatment). Maximal intensity projections of z stacks were made using ImageJ v.1.51p (https://imagej.nih.gov/ij/). Nuclei and γH2AX foci were counted using CellProfiler v.2.2.0 (http://cellprofiler.org), calculating average numbers of γH2AX foci per nucleus in each image. Full details are provided in the Supplemental Materials and Methods.

Animals, Tumor Model, and Tumor Sensitization with Liposomal Alendronate

Approximately 1.5×10^6 MDA-MB-231.hNIS-GFP cells were injected subcutaneously in the mammary fat pad between the fourth and fifth nipples in the left flank; tumors were grown over 3 weeks. Animals were randomly assigned to experimental groups, and investigators were not blinded to cohort allocation when assessing outcomes. Cohort sizes were chosen based on prior experience,[44,50] in compliance with local regulations concerning animal experiments. Liposomal formulations were prepared at Shaare Zedek MC as previously described.[13] Alendronate-loaded liposomes (PLA) contained 1.5−5.4 mg/mL alendronate and 36−40 μmol/mL phospholipids. Placebo liposomes contained 20−50 μmol/mL phospholipids. PLA was co-injected with placebo liposomes for a total dose of 5 mg/kg alendronate and 4 μmol phospholipids per mouse in PLA-treated animals. Placebo-treated animals received empty liposomes corresponding to 4 μmol phospholipids per mouse. Another control group received 5 mg/kg alendronate (ALD). Formulations were injected intravenously (i.v.) 4 days before the administration of radiolabeled γδ-T cells.

In Vivo PET and SPECT Imaging of γδ-T Cells, Tumors, and PLA

^{89}Zr-radiolabeled γδ-T cells (10^7 cells/animal in 100 μL, 0.3−3 MBq ^{89}Zr, single γδ-T donor per experiment) were injected i.v. at t = 0 hr and imaged by PET/CT within 30 min. PET/CT imaging was performed for 30−240 min (as indicated) on a nanoScan PET-CT scanner (Mediso). For tumor imaging, 100 μL $^{99m}TcO_4^-$ (15−25 MBq) in saline was injected i.v., and SPECT-CT was performed 40 min thereafter in a NanoSPECT/CT scanner (Mediso; 1-mm collimators, 30-min scan). PET-CT and SPECT-CT were repeated at t = 48 and 168 hr. For PLA imaging by SPECT-CT, PLA was radiolabeled with [^{111}In]In(oxinate)$_3$ and administered i.v. (7 MBq ^{111}In/mouse) to NSG mice. PET- and SPECT-CT datasets were reconstructed using a Monte Carlo-based full-3D iterative algorithm (Tera-Tomo, Mediso). Images were co-registered and analyzed using VivoQuant v.2.50 (Invicro). Regions of interest (ROIs) were delineated for PET activity quantification in specific organs. Uptake in each ROI was expressed as a percentage of injected dose per volume (% ID/mL).

Ex Vivo Bio-distribution Studies

Mice from imaging studies were used for bio-distribution studies on day 2 or 7. After culling, organs were dissected, weighed, and γ-counted together with standards prepared from a sample of injected material. The percentage of injected dose per gram (% ID/g) of tissue was calculated. Organs were cryopreserved in optimal cutting temperature (OCT) compound (VWR) for autoradiography and/or formalin fixed and paraffin embedded (FFPE) for histologic analysis.

Autoradiography

Cryopreserved tissues were cut (50 μm), mounted on poly-L-lysine-coated slides (VWR), fixed in 4% paraformaldehyde (PFA), mounted in Mowiol, and exposed to a storage phosphor screen for 20 min at 3 hr post-dissection to obtain the ^{99m}Tc signal, then for 48 hr at 4 days post-dissection to obtain the ^{89}Zr signal. The storage phosphor screen was read using a Cyclone Plus imager (PerkinElmer), and images were processed with ImageJ.

Immunohistochemistry

Briefly, FFPE organ blocks were sliced and stained using a Discovery XT system (Ventana Medical Systems) using the DAB Map detection kit (Ventana 760-124). For pre-treatment, CC1 (Ventana 950-124) was used. Sections were stained with anti-GFP (1/1,000; Abcam ab290, UK) or anti-CD3 (LN10, Leica CD3-565-L-CE) primary antibodies, followed by biotinylated anti-rabbit or anti-mouse IgG (1/200; Dako) secondary antibodies, as appropriate. Full details are provided in the Supplemental Materials and Methods.

Statistics

Independent experiments were performed on different days with γδ-T cell batches from different donors. Data were plotted using Prism v.7.01 (GraphPad). Differences between 2 groups were evaluated by Student's two-tailed t test. To account for repeated measurements in a same animal or cell batch and multiple treatments tested on a same cell batch, analysis was performed using 2-way repeated-measures ANOVA in GraphPad Prism or a repeated-measures Mixed Model (MM)[51] in InVivoStat v.3.7 (http://invivostat.co.uk/), as indicated. Dunnett's post hoc test was applied for comparisons back to a control group, or Bonferroni correction for multiple pairwise comparisons, unless otherwise specified. Exact significance values are reported in each figure.

SUPPLEMENTAL INFORMATION

Supplemental Information includes eight figures, three tables, and Supplemental Materials and Methods and can be found with this article online at https://doi.org/10.1016/j.ymthe.2018.10.006.

AUTHOR CONTRIBUTIONS

Conceptualization, F.M., L.L., G.O.F., and R.T.M.d.R.; Methodology, F.M., L.L., A.G., G.O.F., and R.T.M.d.R.; Investigation, F.M., L.L., A.V., B.D., A.C.P.-P., and R.T.M.d.R.; Writing – Original Draft, F.M.; Writing – Review and Editing, F.M., L.L., A.G., J.M., P.J.B., G.O.F., and R.T.M.d.R.; Funding Acquisition, G.O.F., R.T.M.d.R., P.J.B., and J.M.; Resources, F.M., L.L., A.G., H.S., and R.T.M.d.R.; Supervision, R.T.M.d.R., G.O.F., and P.J.B.

CONFLICTS OF INTEREST

J.M. is chief scientific officer of Leucid Bio, a company dedicated to the commercial development of CAR-T cells for solid tumors. The authors declare no other potential conflicts of interest.

ACKNOWLEDGMENTS

The authors thank J. Bartnicka, Dr. J. Bordoloi, A. Dzhatdoeva, P. Gawne, I. Hungnes, M. Iafrate, and G. Keeling for technical assistance. This work was supported by a Cancer Research UK (CRUK) Multidisciplinary Project Award (grant C48390/A21153), the KCL/UCL Comprehensive Cancer Imaging Centre funded by CRUK and EPSRC in association with the MRC and DoH (England), the Wellcome EPSRC Centre for Medical Engineering at KCL (grant WT 203148/Z/16/Z), the Medical Research Council Confidence in Concepts scheme, the Experimental Cancer Medicine Centre at KCL, the KHP/KCL CRUK Cancer Centre and the National Institute for Health Research (NIHR) Biomedical Research Centre based at Guy's and St Thomas' NHS Foundation Trust and KCL (grant IS-BRC-1215-20006). The views expressed are those of the authors and not necessarily those of the NHS, the NIHR, or the Department of Health.

REFERENCES

1. Turtle, C.J., Hay, K.A., Hanafi, L.-A., Li, D., Cherian, S., Chen, X., Wood, B., Lozanski, A., Byrd, J.C., Heimfeld, S., et al. (2017). Durable molecular remissions in chronic lymphocytic leukemia treated with CD19-specific chimeric antigen receptor–modified T cells after failure of ibrutinib. J. Clin. Oncol. *35*, 3010–3020.

2. Chandran, S.S., Somerville, R.P.T., Yang, J.C., Sherry, R.M., Klebanoff, C.A., Goff, S.L., Wunderlich, J.R., Danforth, D.N., Zlott, D., Paria, B.C., et al. (2017). Treatment of metastatic uveal melanoma with adoptive transfer of tumour-infiltrating lymphocytes: a single-centre, two-stage, single-arm, phase 2 study. Lancet Oncol. *18*, 792–802.

3. Rosenberg, S.A., and Restifo, N.P. (2015). Adoptive cell transfer as personalized immunotherapy for human cancer. Science *348*, 62–68.

4. Gentles, A.J., Newman, A.M., Liu, C.L., Bratman, S.V., Feng, W., Kim, D., Nair, V.S., Xu, Y., Khuong, A., Hoang, C.D., et al. (2015). The prognostic landscape of genes and infiltrating immune cells across human cancers. Nat. Med. *21*, 938–945.

5. Tyler, C.J., Doherty, D.G., Moser, B., and Eberl, M. (2015). Human Vγ9/Vδ2 T cells: Innate adaptors of the immune system. Cell. Immunol. *296*, 10–21.

6. Vantourout, P., and Hayday, A. (2013). Six-of-the-best: unique contributions of γδ T cells to immunology. Nat. Rev. Immunol. *13*, 88–100.

7. Bouet-Toussaint, F., Cabillic, F., Toutirais, O., Le Gallo, M., Thomas de la Pintière, C., Daniel, P., Genetet, N., Meunier, B., Dupont-Bierre, E., Boudjema, K., and Catros, V. (2008). Vgamma9Vdelta2 T cell-mediated recognition of human solid tumors. Potential for immunotherapy of hepatocellular and colorectal carcinomas. Cancer Immunol. Immunother. *57*, 531–539.

8. Gu, S., Sachleben, J.R., Boughter, C.T., Nawrocka, W.I., Borowska, M.T., Tarrasch, J.T., Skiniotis, G., Roux, B., and Adams, E.J. (2017). Phosphoantigen-induced conformational change of butyrophilin 3A1 (BTN3A1) and its implication on Vγ9Vδ2 T cell activation. Proc. Natl. Acad. Sci. USA *114*, E7311–E7320.

9. Vavassori, S., Kumar, A., Wan, G.S., Ramanjaneyulu, G.S., Cavallari, M., El Daker, S., Beddoe, T., Theodossis, A., Williams, N.K., Gostick, E., et al. (2013). Butyrophilin 3A1 binds phosphorylated antigens and stimulates human γδ T cells. Nat. Immunol. *14*, 908–916.

10. De Rosa, G., Misso, G., Salzano, G., and Caraglia, M. (2013). Bisphosphonates and cancer: what opportunities from nanotechnology? J. Drug Deliv. *2013*, 637976.

11. Kunzmann, V., Bauer, E., Feurle, J., Weissinger, F., Tony, H.P., and Wilhelm, M. (2000). Stimulation of gammadelta T cells by aminobisphosphonates and induction of antiplasma cell activity in multiple myeloma. Blood *96*, 384–392.

12. Parente-Pereira, A.C., Shmeeda, H., Whilding, L.M., Zambirinis, C.P., Foster, J., van der Stegen, S.J.C., Beatson, R., Zabinski, T., Brewig, N., Sosabowski, J.K., et al. (2014). Adoptive immunotherapy of epithelial ovarian cancer with Vγ9Vδ2 T cells, potentiated by liposomal alendronic acid. J. Immunol. *193*, 5557–5566.

13. Shmeeda, H., Amitay, Y., Gorin, J., Tzemach, D., Mak, L., Stern, S.T., Barenholz, Y., and Gabizon, A. (2016). Coencapsulation of alendronate and doxorubicin in pegylated liposomes: a novel formulation for chemoimmunotherapy of cancer. J. Drug Target. *24*, 878–889.

14. Kobayashi, H., and Tanaka, Y. (2015). γδ T cell immunotherapy—A review. Pharmaceuticals (Basel) *8*, 40–61.

15. Fisher, J.P., Heuijerjans, J., Yan, M., Gustafsson, K., and Anderson, J. (2014). γδ T cells for cancer immunotherapy: A systematic review of clinical trials. OncoImmunology *3*, e27572.

16. James, M.L., and Gambhir, S.S. (2012). A molecular imaging primer: modalities, imaging agents, and applications. Physiol. Rev. *92*, 897–965.

17. Ponomarev, V. (2017). Advancing immune and cell-based therapies through imaging. Mol. Imaging Biol. *19*, 379–384.

18. Volpe, A., Kurtys, E., and Fruhwirth, G.O. (2018). Cousins at work: How combining medical with optical imaging enhances in vivo cell tracking. Int. J. Biochem. Cell Biol. *102*, 40–50.

19. Kircher, M.F., Gambhir, S.S., and Grimm, J. (2011). Noninvasive cell-tracking methods. Nat. Rev. Clin. Oncol. *8*, 677–688.

20. Tavaré, R., McCracken, M.N., Zettlitz, K.A., Knowles, S.M., Salazar, F.B., Olafsen, T., Witte, O.N., and Wu, A.M. (2014). Engineered antibody fragments for immuno-PET imaging of endogenous CD8+ T cells in vivo. Proc. Natl. Acad. Sci. USA *111*, 1108–1113.

21. Mall, S., Yusufi, N., Wagner, R., Klar, R., Bianchi, H., Steiger, K., Straub, M., Audehm, S., Laitinen, I., Aichler, M., et al. (2016). Immuno-PET imaging of engineered human T cells in tumors. Cancer Res. *76*, 4113–4123.

22. Jurgielewicz, P., Harmsen, S., Wei, E., Bachmann, M.H., Ting, R., and Aras, O. (2017). New imaging probes to track cell fate: reporter genes in stem cell research. Cell. Mol. Life Sci. *74*, 4455–4469.

23. Charoenphun, P., Meszaros, L.K., Chuamsaamarkkee, K., Sharif-Paghaleh, E., Ballinger, J.R., Ferris, T.J., Went, M.J., Mullen, G.E., and Blower, P.J. (2015). [$(^{89})$Zr]oxinate$_4$ for long-term in vivo cell tracking by positron emission tomography. Eur. J. Nucl. Med. Mol. Imaging *42*, 278–287.

24. Sato, N., Wu, H., Asiedu, K.O., Szajek, L.P., Griffiths, G.L., and Choyke, P.L. (2015). ^{89}Zr-oxine complex PET cell imaging in monitoring cell-based therapies. Radiology *275*, 490–500.

25. Weist, M.R., Starr, R., Aguilar, B., Chea, J., Miles, J.K., Poku, E., Gerdts, E., Yang, X., Priceman, S.J., Forman, S.J., et al. (2018). PET of adoptively transferred chimeric antigen receptor T cells with ^{89}Zr-oxine. J. Nucl. Med. *59*, 1531–1537.

26. Bonner, W.M., Redon, C.E., Dickey, J.S., Nakamura, A.J., Sedelnikova, O.A., Solier, S., and Pommier, Y. (2008). GammaH2AX and cancer. Nat. Rev. Cancer *8*, 957–967.

27. Volpe, A., Man, F., Lim, L., Khoshnevisan, A., Blower, J., Blower, P.J., and Fruhwirth, G.O. (2018). Radionuclide-fluorescence Reporter Gene Imaging to Track Tumor Progression in Rodent Tumor Models. J. Vis. Exp. (133).

28. Abou, D.S., Ku, T., and Smith-Jones, P.M. (2011). In vivo biodistribution and accumulation of ^{89}Zr in mice. Nucl. Med. Biol. *38*, 675–681.

29. ten Berge, R.J., Natarajan, A.T., Hardeman, M.R., van Royen, E.A., and Schellekens, P.T. (1983). Labeling with indium-111 has detrimental effects on human lymphocytes: concise communication. J. Nucl. Med. *24*, 615–620.

30. Kuyama, J., McCormack, A., George, A.J., Heelan, B.T., Osman, S., Batchelor, J.R., and Peters, A.M. (1997). Indium-111 labelled lymphocytes: isotope distribution and cell division. Eur. J. Nucl. Med. *24*, 488–496.

31. Jauw, Y.W.S., Menke-van der Houven van Oordt, C.W., Hoekstra, O.S., Hendrikse, N.H., Vugts, D.J., Zijlstra, J.M., Huisman, M.C., and van Dongen, G.A. (2016). Immuno-Positron Emission Tomography with Zirconium-89-Labeled Monoclonal Antibodies in Oncology: What Can We Learn from Initial Clinical Trials? Front. Pharmacol. *7*, 131.

32. Zhang, X., Zhou, J., Cherry, S.R., Badawi, R.D., and Qi, J. (2017). Quantitative image reconstruction for total-body PET imaging using the 2-meter long EXPLORER scanner. Phys. Med. Biol. *62*, 2465–2485.

33. Hodgins, N.O., Al-Jamal, W.T., Wang, J.T.-W., Klippstein, R., Costa, P.M., Sosabowski, J.K., Marshall, J.F., Maher, J., and Al-Jamal, K.T. (2017). Investigating in vitro and in vivo αvβ6 integrin receptor-targeting liposomal alendronate for combinatory γδ T cell immunotherapy. J. Control. Release *256*, 141–152.

34. Pang, D.J., Neves, J.F., Sumaria, N., and Pennington, D.J. (2012). Understanding the complexity of γδ T-cell subsets in mouse and human. Immunology *136*, 283–290.

35. Beck, B.H., Kim, H.-G., Kim, H., Samuel, S., Liu, Z., Shrestha, R., Haines, H., Zinn, K., and Lopez, R.D. (2010). Adoptively transferred ex vivo expanded gammadelta-T cells mediate in vivo antitumor activity in preclinical mouse models of breast cancer. Breast Cancer Res. Treat. *122*, 135–144.

36. Nicol, A.J., Tokuyama, H., Mattarollo, S.R., Hagi, T., Suzuki, K., Yokokawa, K., and Nieda, M. (2011). Clinical evaluation of autologous gamma delta T cell-based immunotherapy for metastatic solid tumours. Br. J. Cancer *105*, 778–786.

37. Meidenbauer, N., Marienhagen, J., Laumer, M., Vogl, S., Heymann, J., Andreesen, R., and Mackensen, A. (2003). Survival and tumor localization of adoptively transferred Melan-A-specific T cells in melanoma patients. J. Immunol. *170*, 2161–2169.

38. Bobisse, S., Rondina, M., Merlo, A., Tisato, V., Mandruzzato, S., Amendola, M., Naldini, L., Willemsen, R.A., Debets, R., Zanovello, P., and Rosato, A. (2009). Reprogramming T lymphocytes for melanoma adoptive immunotherapy by T-cell receptor gene transfer with lentiviral vectors. Cancer Res. *69*, 9385–9394.

39. Ma, M.T., Meszaros, L.K., Paterson, B.M., Berry, D.J., Cooper, M.S., Ma, Y., Hider, R.C., and Blower, P.J. (2015). Tripodal tris(hydroxypyridinone) ligands for immunoconjugate PET imaging with $^{(89)}$Zr$^{(4+)}$: comparison with desferrioxamine-B. Dalton Trans. *44*, 4884–4900.

40. Roullet, M., Gheith, S.M.F., Mauger, J., Junkins-Hopkins, J.M., and Choi, J.K. (2009). Percentage of γδ T cells in panniculitis by paraffin immunohistochemical analysis. Am. J. Clin. Pathol. *131*, 820–826.

41. Van Acker, H.H., Anguille, S., Willemen, Y., Smits, E.L., and Van Tendeloo, V.F. (2016). Bisphosphonates for cancer treatment: Mechanisms of action and lessons from clinical trials. Pharmacol. Ther. *158*, 24–40.

42. Prabhakar, U., Maeda, H., Jain, R.K., Sevick-Muraca, E.M., Zamboni, W., Farokhzad, O.C., Barry, S.T., Gabizon, A., Grodzinski, P., and Blakey, D.C. (2013). Challenges and key considerations of the enhanced permeability and retention effect for nanomedicine drug delivery in oncology. Cancer Res. *73*, 2412–2417.

43. Hodgins, N.O., Al-Jamal, W.T., Wang, J.T.-W., Parente-Pereira, A.C., Liu, M., Maher, J., and Al-Jamal, K.T. (2016). In vitro potency, in vitro and in vivo efficacy of liposomal alendronate in combination with γδ T cell immunotherapy in mice. J. Control. Release *241*, 229–241.

44. Edmonds, S., Volpe, A., Shmeeda, H., Parente-Pereira, A.C., Radia, R., Baguña-Torres, J., Szanda, I., Severin, G.W., Livieratos, L., Blower, P.J., et al. (2016). Exploiting the Metal-Chelating Properties of the Drug Cargo for In Vivo Positron Emission Tomography Imaging of Liposomal Nanomedicines. ACS Nano *10*, 10294–10307.

45. Dillman, R.O., Hurwitz, S.R., Schiltz, P.M., Barth, N.M., Beutel, L.D., Nayak, S.K., and O'Connor, A.A. (1997). Tumor localization by tumor infiltrating lymphocytes labeled with indium-111 in patients with metastatic renal cell carcinoma, melanoma, and colorectal cancer. Cancer Biother. Radiopharm. *12*, 65–71.

46. Kershaw, M.H., Westwood, J.A., Parker, L.L., Wang, G., Eshhar, Z., Mavroukakis, S.A., White, D.E., Wunderlich, J.R., Canevari, S., Rogers-Freezer, L., et al. (2006). A phase I study on adoptive immunotherapy using gene-modified T cells for ovarian cancer. Clin. Cancer Res. *12*, 6106–6115.

47. Ritchie, D.S., Neeson, P.J., Khot, A., Peinert, S., Tai, T., Tainton, K., Chen, K., Shin, M., Wall, D.M., Hönemann, D., et al. (2013). Persistence and efficacy of second generation CAR T cell against the LeY antigen in acute myeloid leukemia. Mol. Ther. *21*, 2122–2129.

48. Botti, C., Negri, D.R., Seregni, E., Ramakrishna, V., Arienti, F., Maffioli, L., Lombardo, C., Bogni, A., Pascali, C., Crippa, F., et al. (1997). Comparison of three different methods for radiolabelling human activated T lymphocytes. Eur. J. Nucl. Med. *24*, 497–504.

49. Griffith, K.D., Read, E.J., Carrasquillo, J.A., Carter, C.S., Yang, J.C., Fisher, B., Aebersold, P., Packard, B.S., Yu, M.Y., and Rosenberg, S.A. (1989). In vivo distribution of adoptively transferred indium-111-labeled tumor infiltrating lymphocytes and peripheral blood lymphocytes in patients with metastatic melanoma. J. Natl. Cancer Inst. *81*, 1709–1717.

50. Fruhwirth, G.O., Diocou, S., Blower, P.J., Ng, T., and Mullen, G.E.D. (2014). A whole-body dual-modality radionuclide optical strategy for preclinical imaging of metastasis and heterogeneous treatment response in different microenvironments. J. Nucl. Med. *55*, 686–694.

51. Wang, Z., and Goonewardene, L.A. (2004). The use of MIXED models in the analysis of animal experiments with repeated measures data. Can. J. Anim. Sci. *84*, 1–11.

YMTHE, Volume *27*

Supplemental Information

In Vivo PET Tracking of ^{89}Zr-Labeled Vγ9Vδ2 T Cells to Mouse Xenograft Breast Tumors Activated with Liposomal Alendronate

Francis Man, Lindsay Lim, Alessia Volpe, Alberto Gabizon, Hilary Shmeeda, Benjamin Draper, Ana C. Parente-Pereira, John Maher, Philip J. Blower, Gilbert O. Fruhwirth, and Rafael T.M. de Rosales

Supplemental Figures

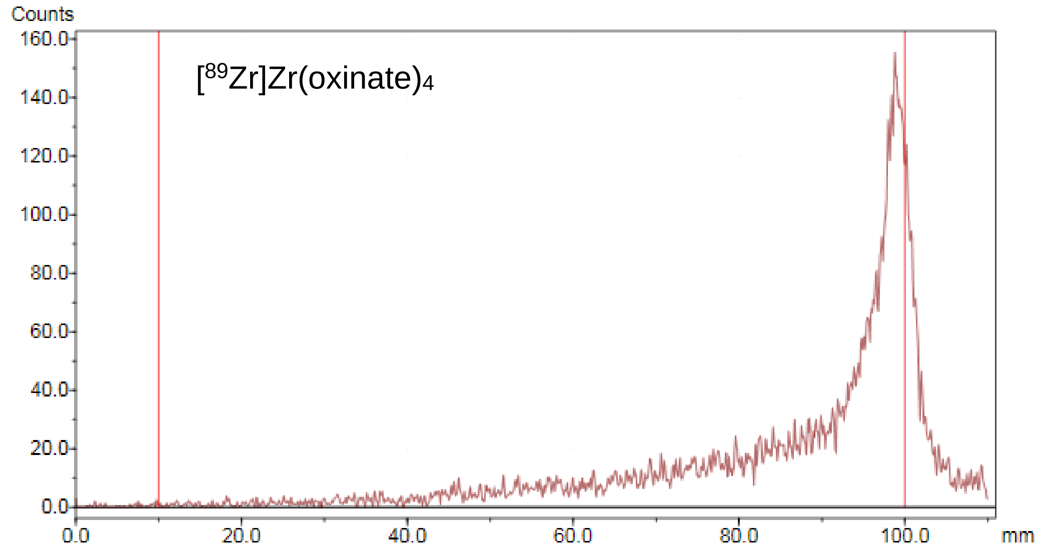

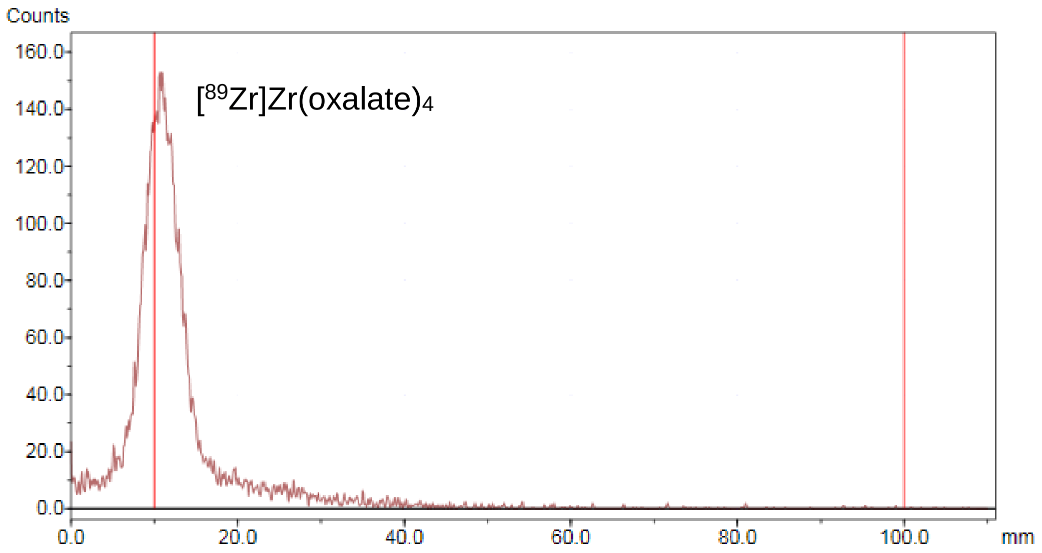

Figure S1. Representative radioTLC chromatograms of [^{89}Zr]Zr(oxinate)$_4$ (top, $R_F \approx 1$) and [^{89}Zr]Zr(oxalate)$_4$ (bottom, $R_F \approx 0$).

A solution containing $^{89}Zr^{4+}$ in 1 M oxalic acid was diluted with H_2O, neutralized with 1 M Na_2CO_3 and an aliquot spotted onto an ITLC-SG plate, dried and run in 100 % EtOAc. To the neutralized solution, 8-hydroxyquinoline (oxine) in $CHCl_3$ was added and the mixture was vortexed. The organic layer was extracted and dried at 60 °C. The residue was dissolved in DMSO, spotted onto an ITLC-SG plate, dried and run in 100 % EtOAc. The ITLC plates were read on a linear radioTLC scanner equipped with a β^+ probe.

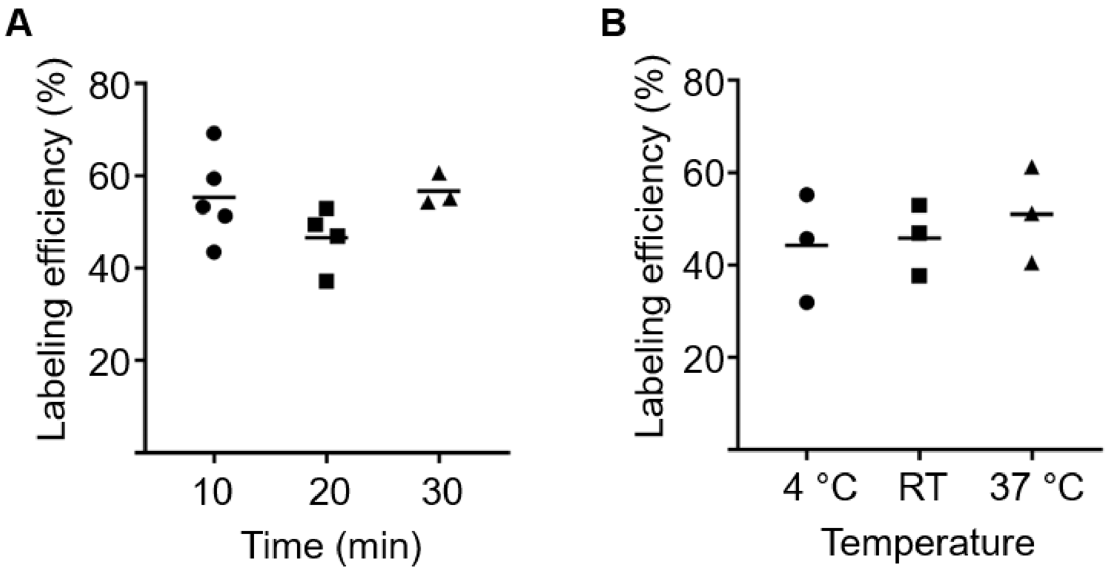

Figure S2. *In vitro* labeling of γδ-T cells with [^{89}Zr]Zr(oxinate)$_4$.

Labeling efficiencies of γδ-T cells incubated with [^{89}Zr]Zr(oxinate)$_4$ (69.0 ± 7.9 mBq/cell) at room temperature for varying amounts of time (**A**), or for 20 min at varying temperatures (**B**). Bars represent the average of N = 3-5 individual experiments, each dot within a group representing cells from a different donor.

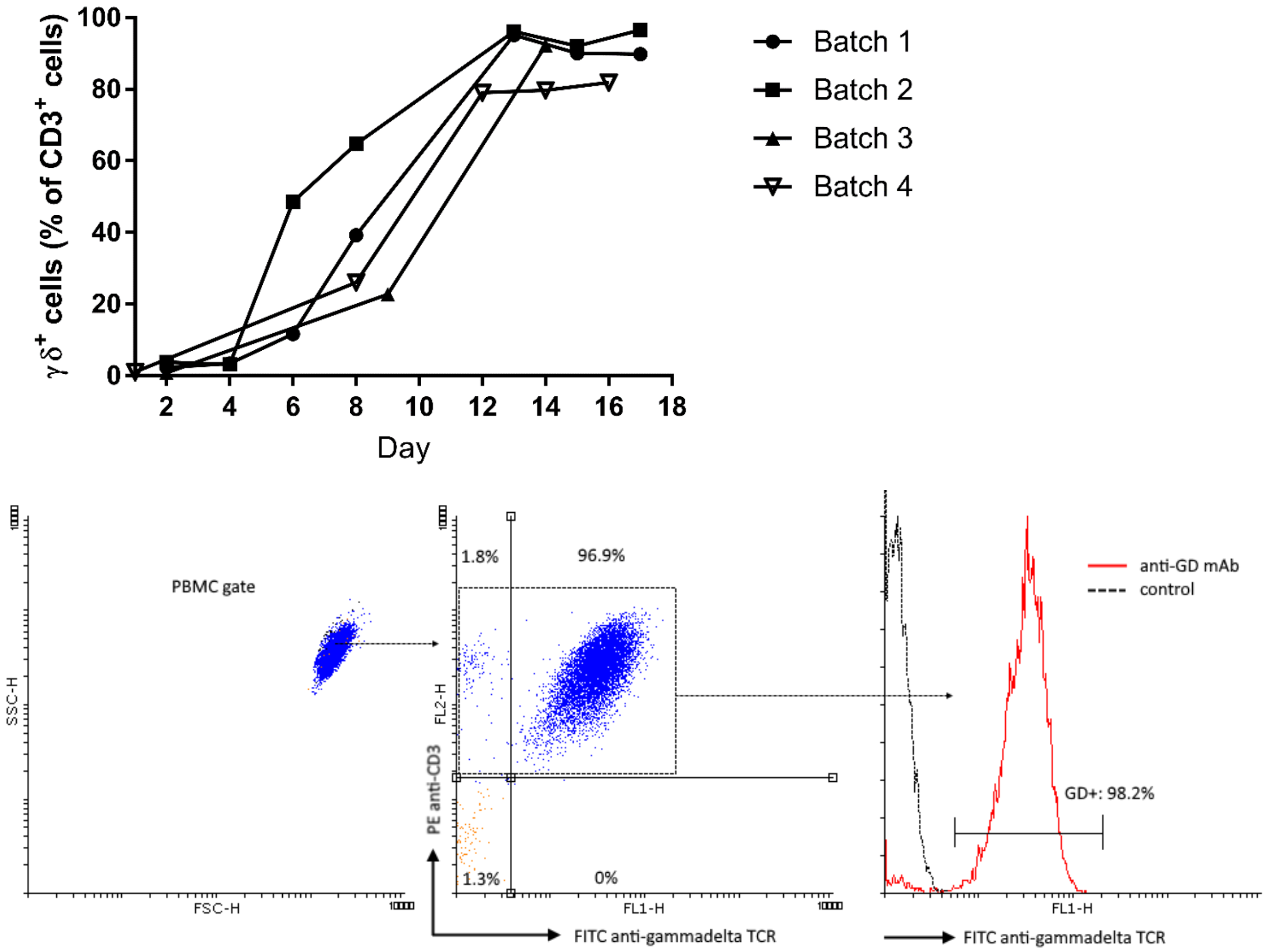

Figure S3. Population purity of γδ-T cells during *in vitro* expansion and flow cytometry analysis.

Top: expansion of the γδ-T cell population from PBMCs isolated from 4 different donors. *Bottom:* gating strategy and example flow cytometry plot of *in vitro* expanded human γδ-T cells on day 14 after isolation. Human PBMCs were isolated by density centrifugation, resuspended in RPMI + 10 % human serum and treated with 3.7 µM zoledronate on the first day and with 100 IU IL-2 every 2-3 days. Aliquots of cultured cells were stained with a FITC-conjugated anti-pan-γδ TCR mAb (IMMU510) and a PE-conjugated anti-CD3 antibody (OKT3) and analyzed by flow cytometry. PBMCs were gated by forward/side scatter and the percentage of γδ-positive events amongst $CD3^+$ events was calculated.

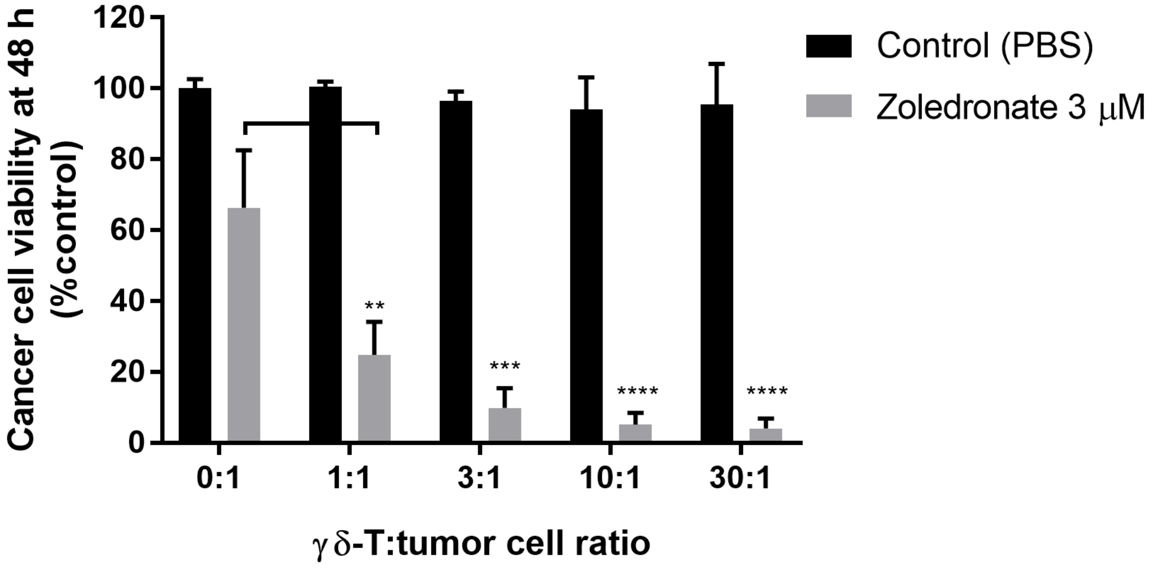

Figure S4. γδ-T cytotoxicity with or without treatment of cancer cells with zoledronate.

MDA-MD-231.hNIS-GFP breast cancer cells were grown to confluence in a 96-well plate (10^4 cells seeded per well) and treated with 3 μM zoledronate or vehicle for 24 h. The medium was then replaced and increasing amounts of γδ-T cells were added for 48 h. Cell viability was measured using the alamarBlue™ assay. Viability is expressed as the percentage of control (cancer cells treated with PBS only, without γδ-T cells). Mean ± SEM of $N = 4$ independent experiments with measurements performed in triplicate. ***P* = 0.0025, ****P* = 0.0002, *****P* < 0.0001 *vs*. “Zoledronate 3 μM 0:1” (2-way repeated-measures ANOVA with Dunnett’s correction for multiple comparisons).

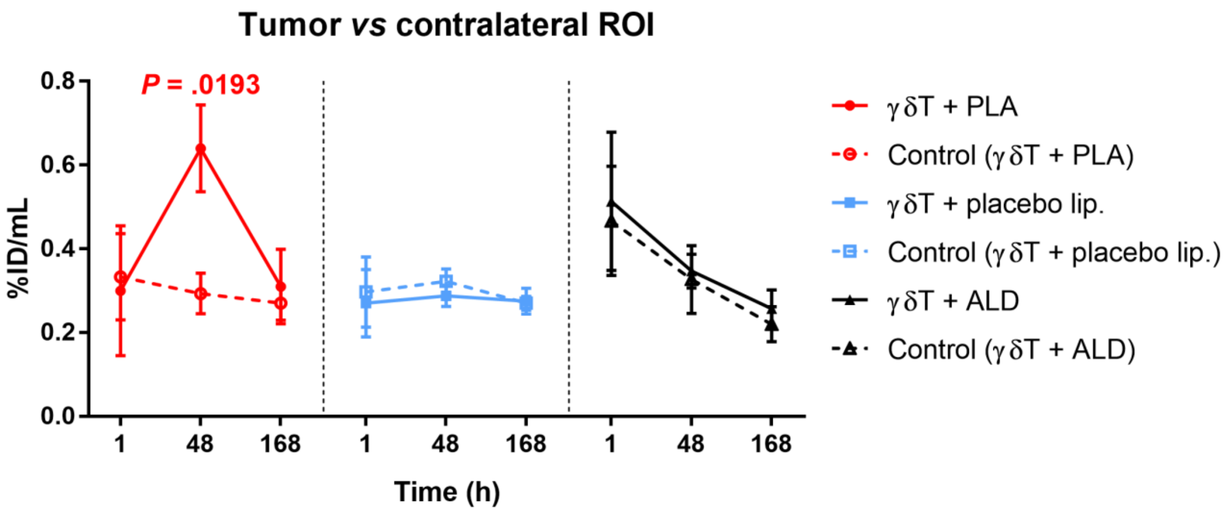

Figure S5. Image-based quantification control**.**

To determine background values of the PET signal in PET-CT imaging studies, a region-of-interest of similar size to that of the tumor was identified by CT imaging and drawn in a contralateral area of each animal, and the amount of ^{89}Zr, expressed as %ID/mL, was determined. Mean ± SEM of N = 3-4 animals per group. Solid lines represent the amounts of ^{89}Zr in the tumors, dashed lines represent the amount of ^{89}Zr in contralateral regions. Repeated-measures MM analysis within PLA group, only significant P values ($P < .05$ *vs*. control group) are reported.

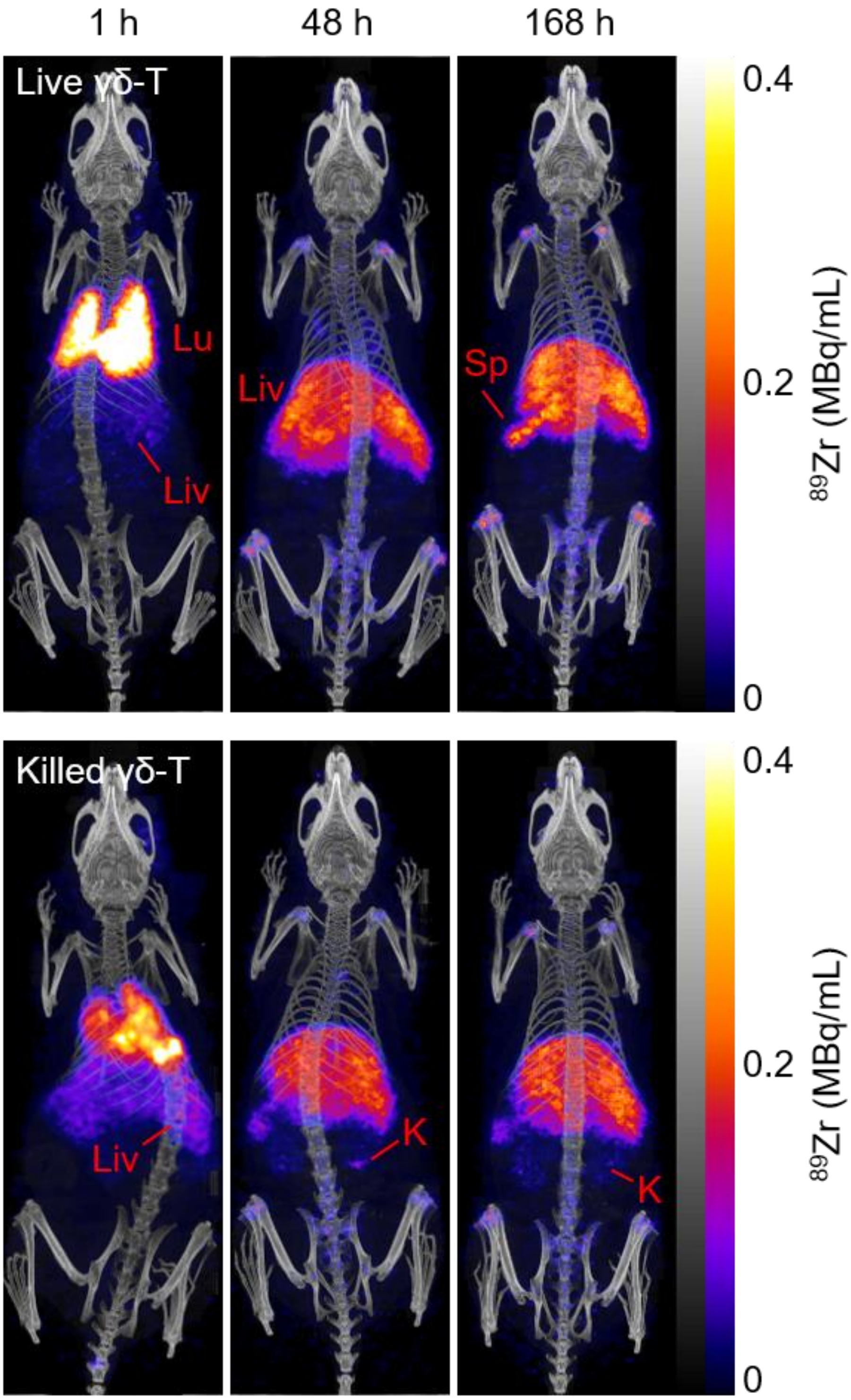

Figure S6. PET/CT imaging of live vs killed ^{89}Zr-labeled γδ-T cells.

Representative maximal intensity projections of PET/CT images of live (top) vs killed (bottom) γδ-T cells radiolabeled with [^{89}Zr]Zr(oxinate)$_4$ (30 mBq/cell) at 1 h, 48 h and 168 h post-injection. Killed γδ-T cells were obtained by subjecting them (after radiolabeling) to 2 freeze-thaw cycles (30 min at −20 °C) before i.v. injection. Lu: lungs; Liv: liver; Sp: spleen; K: kidney.

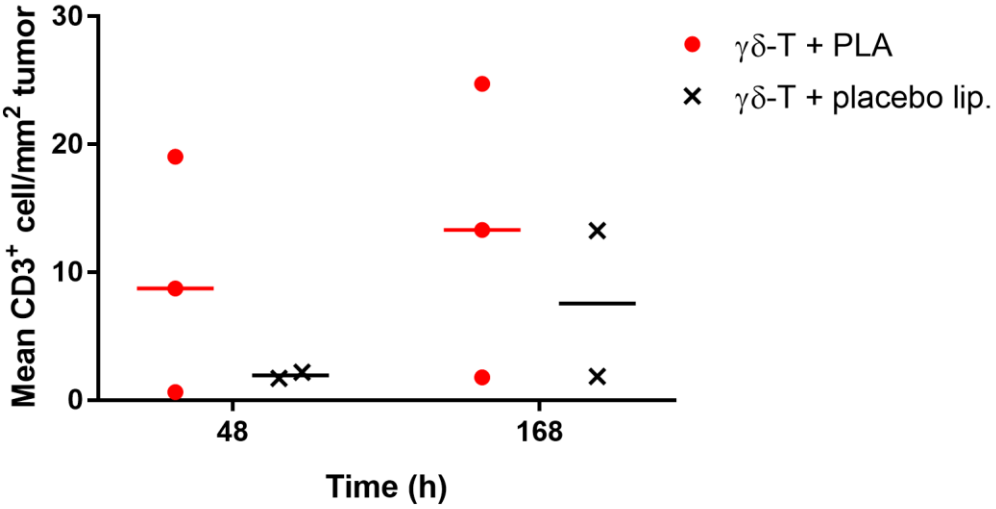

Figure S7. Histology of γδ-T cells (quantification) in tumors.

CD3-positive cells were counted in paraffin-embedded tumor sections from animals (1 tumor/animal) treated with PLA or placebo liposomes, 48 h or 168 h after intravenous administration of ^{89}Zr-labeled γδ-T cells. The number of cells in each section was divided by the total area of the section. Each point represents the average of 3 sections from the same tumor, bars represent the median of 2 or 3 tumors per group.

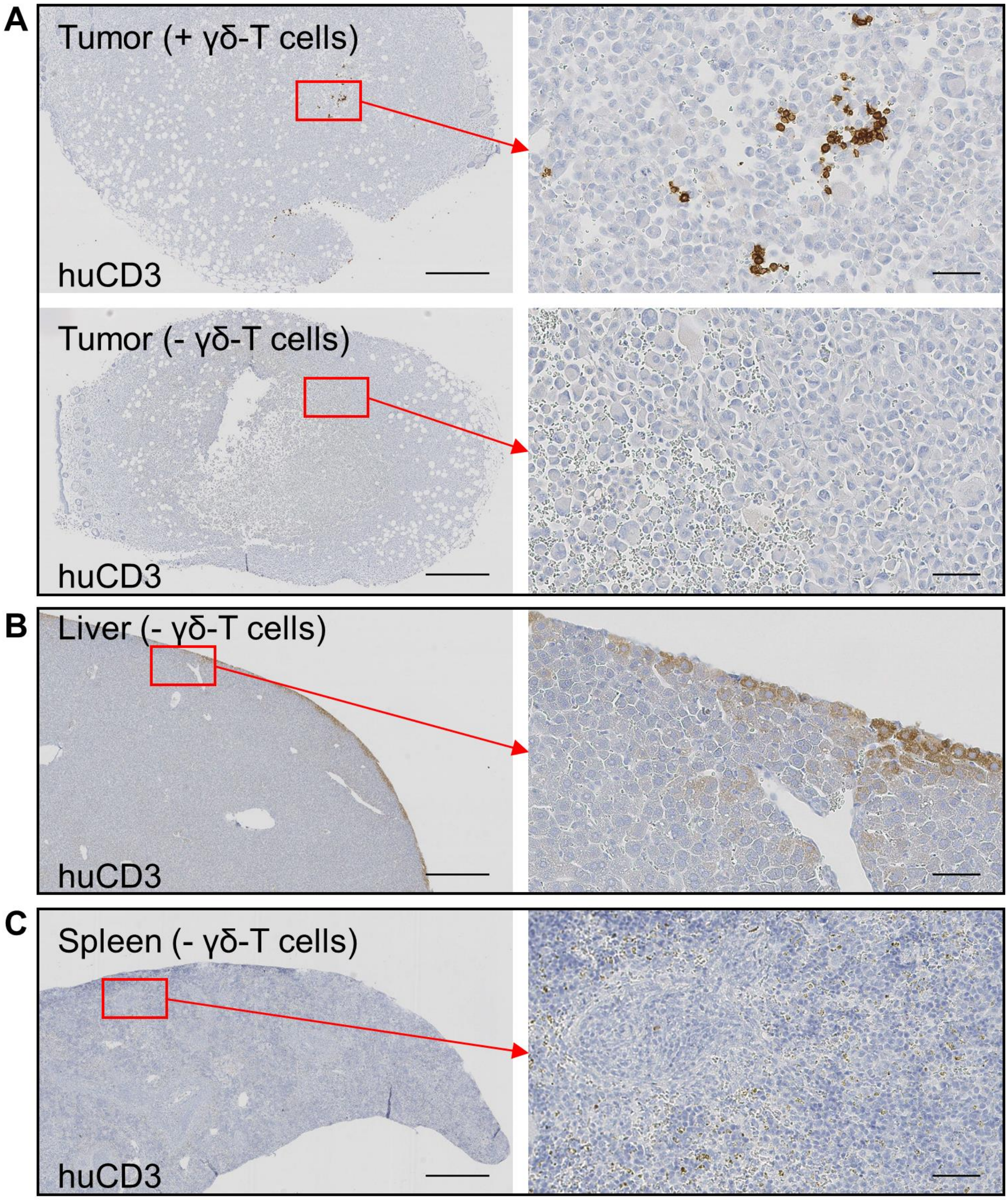

Figure S8. Histology of γδ-T cells (controls).

A Representative slices of MDA-MB-231.hNIS-GFP tumors grown on the same SCID/beige mouse (not administered human γδ-T cells). The animal was euthanized and the tumors surgically removed. The tumor on the top was then injected *ex vivo* with human γδ-T cells, and both tumors were formalin-fixed and embedded in paraffin. **B,C** Representative slices of liver (**B**) and spleen (**C**) sections from control SCID/beige mice (not administered human γδ-T cells). Sections were stained for human CD3. 4/30× magnification; scale bars = 500 µm (left) or 50 µm (right).

Supplemental Tables

Table S1. Image-based quantification of ^{111}In uptake in the tumor.

Female NSG mice bearing MDA-MB-231.hNIS-GFP tumors were administered ^{111}In-labeled PEGylated liposomal alendronate intravenously and imaged by SPECT/CT after 24 h and 72 h.

**Time after ^{111}In-PLA administration**	**24 h**	**72 h**
	**^{111}In uptake in tumor, mean ± SD**	
**% injected dose (%ID)**	4.7 ± 1.7	10.9 ± 2.7
**% injected dose per unit volume (%ID/mL)**	8.7 ± 1.2	12.9 ± 0.8

Table S2. Image-based PET quantification data of ^{89}Zr-labeled γδ-T cells.

Uptake of ^{89}Zr at the tumor site was higher in PLA-treated animals 48 h after administration of radiolabeled γδ-T cells.

Treatment	γδ-T + PLA			γδ-T + placebo liposomes			γδ-T + ALD		
Number of animals	3			3			3		
**Day post-injection**	**1**	**3**	**7**	**1**	**3**	**7**	**1**	**3**	**7**
**Organ**	**%ID/mL (mean ± SD)**								
Stomach	3.74 ± 0.90	11.27 ± 1.90	8.85 ± 3.32	7.44 ± 8.04	11.18 ± 1.25	11.37 ± 4.27	7.77 ± 4.75	12.50 ± 0.14	9.94 ± 3.14
**Tumor**	0.30 ± 0.27	**0.64 ± 0.18**	0.31 ± 0.16	0.27 ± 0.14	**0.29 ± 0.05**	0.27 ± 0.06	0.51 ± 0.29	**0.35 ± 0.07**	0.26 ± 0.08
Thyroid + salivary glands	0.59 ± 0.26	0.76 ± 0.04	0.91 ± 0.50	0.76 ± 0.30	0.94 ± 0.23	0.94 ± 0.26	0.91 ± 0.10	0.81 ± 0.28	0.91 ± 0.20
Kidney	3.18 ± 1.54	3.45 ± 0.49	3.91 ± 0.37	3.34 ± 1.45	4.38 ± 0.21	5.39 ± 2.41	5.49 ± 0.76	4.29 ± 0.34	4.86 ± 0.64
Bone	1.38 ± 0.99	5.75 ± 0.87	8.83 ± 0.90	2.51 ± 1.46	8.23 ± 0.88	11.32 ± 2.29	3.07 ± 1.60	7.80 ± 0.71	11.17 ± 3.34
Muscle	0.25 ± 0.16	0.41 ± 0.03	0.37 ± 0.11	0.32 ± 0.12	0.51 ± 0.05	0.39 ± 0.26	0.42 ± 0.10	0.33 ± 0.05	0.38 ± 0.08
Lung	42.33 ± 22.84	5.22 ± 1.07	5.21 ± 0.41	33.33 ± 26.88	5.30 ± 1.05	6.04 ± 0.45	29.64 ± 19.01	5.06 ± 0.50	5.48 ± 0.49
Liver	22.87 ± 10.88	40.66 ± 3.82	44.75 ± 0.62	29.10 ± 10.00	41.19 ± 5.47	48.37 ± 4.12	40.41 ± 3.55	43.48 ± 5.04	48.00 ± 4.25
Spleen	14.45 ± 8.42	30.08 ± 5.28	34.51 ± 4.49	16.42 ± 7.42	30.59 ± 1.32	40.54 ± 3.91	24.90 ± 6.97	32.17 ± 6.20	42.07 ± 12.22
Tumor-contralateral ROI	0.33 ± 0.18	0.29 ± 0.08	0.27 ± 0.07	0.30 ± 0.14	0.32 ± 0.06	0.27 ± 0.03	0.47 ± 0.23	0.33 ± 0.14	0.22 ± 0.07

Table S3. *Ex vivo* bio-distribution data of ^{89}Zr after administration of ^{89}Zr-labeled γδ-T cells.

Uptake of ^{89}Zr at the tumor site was higher in PLA-treated animals 7 days after administration of radiolabeled γδ-T cells.

Group	**A**	**B**	**C**	**D**	**E**	**F**
Treatment	γδ-T + PLA	γδ-T + PLA	γδ-T only	γδ-T + placebo liposomes	γδ-T + ALD	γδ-T (killed)
^{89}Zr per cell (mBq)	30	300	30	300	300	30
Number of animals	6	3	5	4	3	3
**Organ**	**%ID/g (mean ± SD) 7 days post-injection**					
Blood	0.87 ± 0.83	2.07 ± 2.02	0.55 ± 0.38	1.66 ± 0.75	1.91 ± 0.87	0.24 ± 0.04
Bone	5.45 ± 1.95	8.66 ± 0.95	10.72 ± 5.42	9.85 ± 1.20	9.12 ± 3.92	4.55 ± 2.73
Heart	1.19 ± 0.41	1.61 ± 0.17	1.14 ± 0.52	1.53 ± 0.15	2.6 ± 1.54	0.50 ± 0.29
Intestines	0.31 ± 0.04	0.30 ± 0.02	0.29 ± 0.07	0.31 ± 0.02	0.28 ± 0.01	0.27 ± 0.08
Kidney	5.19 ± 1.51	3.22 ± 0.12	7.26 ± 1.64	3.94 ± 0.57	3.81 ± 0.24	11.91 ± 1.50[a]
Liver	51.65 ± 17.71	64.17 ± 5.73	54.62 ± 4.72	59.85 ± 7.15	58.96 ± 4.49	67.71 ± 8.99
Lung	8.37 ± 5.24	9.89 ± 2.65	10.36 ± 5.87	10.41 ± 0.87	8.34 ± 3.51	4.53 ± 2.61
Muscle	0.30 ± 0.07	0.96 ± 0.37	0.23 ± 0.05	0.73 ± 0.25	0.60 ± 0.37	0.16 ± 0.03
Thyroid + salivary glands	0.92 ± 0.23	1.12 ± 0.07	0.69 ± 0.07	1.30 ± 0.18	1.15 ± 0.17	0.55 ± 0.05
Skin + fur	1.01 ± 0.37	1.78 ± 0.61	0.60 ± 0.18	1.34 ± 0.31	1.43 ± 0.41	0.52 ± 0.14
Spleen	92.80 ± 50.96	226.09 ± 33.98	115.56 ± 84.27	217.92 ± 35.08	278.07 ± 77.85	55.40 ± 15.67
Stomach	0.42 ± 0.17	0.51 ± 0.28	0.41 ± 0.13	0.72 ± 0.11	0.81 ± 0.40	0.41 ± 0.21
**Tumor**	**1.99 ± 0.61**	**2.32 ± 1.28**	1.11 ± 0.39	1.19 ± 0.31	1.27 ± 0.13	0.98 ± 0.18

[a]$p < 0.001$ *vs*. all other groups (1-way ANOVA with Tukey's test for multiple comparisons).

Supplemental Methods

Synthesis of [^{89}Zr]Zr(oxinate)$_4$

No-carrier-added ^{89}Zr (produced at the BV Cyclotron, VU Amsterdam, NL) was purchased from PerkinElmer as H_4[^{89}Zr(oxalate)$_4$] in 1 M oxalic acid. The acidic ^{89}Zr solution (5−100 MBq) was transferred to a 1.5 mL plastic vial, diluted to 150 µL with Chelex®-treated water and gradually adjusted to pH 7.5−8 (measured with pH strips) with 1 M sodium carbonate. The volume was then adjusted to 450 µL with Chelex®-treated water and 50 µL of a 10 mg/mL solution of 8-hydroxyquinoline in chloroform was added. The mixture was vortexed for 5 min, a further 450 µL of chloroform were added and the mixture vortexed for 10 min. The organic phase was extracted into a conical glass vial and dried at 60 °C under a flow of nitrogen gas. The residue was dissolved in aqueous dimethyl sulfoxide (DMSO, 30 ± 10 % in water) for further use. Radiochemical yield was defined by the amount of radioactivity present in the dried organic extract divided by the starting amount of radioactivity. Radioactivity in samples was measured with a CRC-25R dose calibrator (Capintec). Product formation was confirmed by radioTLC on instant thin-layer chromatography (ITLC)-SG paper (Macherey-Nagel) using 100 % ethyl acetate as the mobile phase. ITLC plates were read using a Mini-Scan™ radioTLC linear scanner (LabLogic Systems) equipped with a β^+ probe (LabLogic B-FC-3600). Radiochemical purity of the final product was determined as the radioactivity associated with the [^{89}Zr]Zr(oxinate)$_4$ peak divided by the total detected radioactivity on the chromatogram.

Isolation and *in vitro e*xpansion of Vγ9Vδ2 T cells

Peripheral blood was collected from healthy, male and female donors aged 22-45. Citrate-anticoagulated blood (20−30 mL) was layered over 15 mL of Ficoll-Paque Plus (GE Healthcare) and centrifuged for 30 min at 750 rcf. The buffy coat containing peripheral blood mononuclear cells (PBMCs) was extracted, diluted with PBS and centrifuged for 10 min at 200 rcf. The supernatant was discarded and the pellet re-suspended in PBS, then centrifuged for 10 min at 550 rcf. PBMCs were then re-suspended at 3×10^6/mL in growth medium (RPMI-1640 supplemented with 10 % human AB serum, 100 U/mL penicillin, 0.1 mg/mL streptomycin and 2 mM L-alanyl-L-glutamine (Glutamax; Gibco)). On the first day, 3.7 µM zoledronic acid (Novartis) and 100 IU/mL IL-2 (Novartis) were added, and the cells incubated at 37 °C in a 5 % CO_2 atmosphere. IL-2 (100 IU/mL) and fresh medium were then added every 2-3 days. γδ-T cells were used on day 13-14 after isolation. Population purity was assessed by flow cytometry (BD FACSCalibur), using a FITC-conjugated pan-γδ TCR monoclonal antibody (mAb) (clone IMMU510; Beckman Coulter) and a PE-conjugated anti-CD3 mAb (clone OKT3; BioLegend). PBMCs were gated by forward/side scatter and a minimum of 10,000 events in the PBMC gate were analyzed.

Radiotracer retention and cell proliferation

Approximately 2.5×10^6 radiolabeled (or vehicle-treated) γδ-T cells were re-suspended in growth medium, seeded in 6-well plates and cultured as described above, with fresh medium and IL-2 added every 2 days. At t = 0, 24, 48, 96, 144 and 192 h, the cells were gently re-suspended and 200−300 µL aliquots were removed for analysis. The number of live cells was counted using Trypan Blue. Cells were pelleted and washed with PBS for γ-counting as described above to determine the percentage of cell-associated radioactivity at each time point. Washed cells then stained with a FITC-conjugated pan-γδ TCR mAb (IMMU510) and propidium iodide (PI; Thermo Scientific) and analyzed by flow cytometry as described above to determine the percentage of dead cells (PI-positive) in the $\gamma\delta^+$ population.

Determination of DNA double-strand breaks

γδ-T cells radiolabeled as described in the manuscript and suspended in RPMI were seeded in duplicate onto poly-L-lysine-coated glass coverslips, at 2.5×10^5 cells per coverslip. After 1 h incubation at 37 °C, the plate was centrifuged, the supernatant was removed and the cells were fixed and permeabilized with 3.7 % formalin, 0.5 % Triton X-100 and 0.5 % IGEPAL® CA-630 in PBS. The cells were blocked with 2 % bovine serum albumin and 1 % goat serum in PBS. The cells were then incubated with an anti-phospho-histone H2A.X (Ser139) mouse mAb (1:1600; clone JBW301, Merck #05-636) overnight at 4 °C, then with an Alexa Fluor™ 488-conjugated goat anti-mouse IgG (H+L) secondary antibody (1:500; Jackson ImmunoResearch Laboratories #115-545-062) for 2 h at RT, and Hoechst 33342 for 1 min at RT. After mounting onto glass slides, images of cell nuclei and γH2AX foci were acquired on a TCS SP5 II confocal microscope (Leica) equipped with a 100×/1.40 HCX PL Apochromat oil immersion objective (Leica) and the Leica LAS-AF control software. Scanning was performed using 405 nm (blue diode) and 488 nm (argon) laser lines with a pinhole of 153 µm. Ten contiguous, non-overlapping optical sections of approximately 0.4 µm thickness through the cell nuclei were imaged. At least 30 nuclei per slide were imaged, with 2 slides per treatment. Illumination and acquisition parameters were kept constant within an experiment. Maximal intensity projections of *z*-stacks in

each channel were made using ImageJ v1.51p (http://imagej.nih.gov/ij). Nuclei and γH2AX foci were then counted using CellProfiler (http://cellprofiler.org) v2.2.0[1]. Nuclei (blue channel) were detected using a shape-based method (size filter: 30-80 pixels) to create mask images. Nuclei touching the edge of the images or too clumped to be distinguished by the software were excluded. A speckle-enhancing algorithm was applied to γH2AX images (green channel), followed by the mask image to remove out-of-nucleus signal. γH2AX foci were then detected on an intensity-based method with a size filter of 3-9 pixels, assigned to the corresponding nuclei and counted. The average number of γH2AX foci per nucleus was calculated for each image.

Immunohistochemistry

FFPE organs were processed by UCL IQPath (London, UK) for histologic analysis. FFPE organ blocks were sliced and stained with hematoxylin & eosin. Immunohistochemistry was performed with a Discovery XT system (Ventana Medical Systems) using the DAB Map detection kit (Ventana #760-124). For pre-treatment, CC1 (Ventana #950-124) was used. Sections were stained with anti-GFP (for tumor cell detection; rabbit polyclonal, 1/1000, Abcam #ab290, UK), anti-gamma/delta TCR (for γδ-T cell detection; clone 5A6.E9, ThermoFisher #TCR1061; clone B1.1, eBioscience #16-9959-81) or anti-CD3 (for human T cell detection; clone LN10, Leica #CD3-565-L-CE) primary antibodies, followed by biotinylated anti-rabbit or anti-mouse IgG (1/200; Dako) secondary antibodies, as appropriate. To the best of our knowledge, the only monoclonal antibodies that detect human γδ-TCR in paraffin-embedded samples are clones γ3.20, B1 and 5A6.E9[2–4], of which clone γ3.20 is no longer commercially available. We tested both B1 and 5A6.E9 and were unable to distinguish between control tumors devoid of γδ-T cells and tumors directly injected with γδ-T cells. Human CD3 was therefore used as a surrogate marker for γδ-T cells (Figure 5 and Figure S8).

Supplemental References

1. Carpenter, A.E., Jones, T.R., Lamprecht, M.R., Clarke, C., Kang, I., Friman, O., et al. (2006). CellProfiler: image analysis software for identifying and quantifying cell phenotypes. Genome Biol. *7*, R100.

2. Roullet, M., Gheith, S.M.F., Mauger, J., Junkins-Hopkins, J.M. and Choi, J.K. (2009). Percentage of γδ T cells in panniculitis by paraffin immunohistochemical analysis. Am. J. Clin. Pathol. *131*, 820–826.

3. Pollinger, B., Junt, T., Metzler, B., Walker, U.A., Tyndall, A., Allard, C., et al. (2011). Th17 cells, not IL-17+ T cells, drive arthritic bone destruction in mice and humans. J. Immunol. *186*, 2602–2612.

4. Tanaka, T., Yamamoto, H., Elsayed, A.A., Satou, A., Asano, N., Kohno, K., et al. (2016). Clinicopathologic spectrum of gastrointestinal T-cell lymphoma. Am. J. Surg. Pathol. *40*, 777–785.
